# Theranostic imaging and multimodal photodynamic therapy and immunotherapy using the mTOR signaling pathway

**DOI:** 10.1038/s41467-023-40826-5

**Published:** 2023-09-02

**Authors:** Huiling Zhou, Dongsheng Tang, Yingjie Yu, Lingpu Zhang, Bin Wang, Johannes Karges, Haihua Xiao

**Affiliations:** 1 Beijing National Laboratory for Molecular Sciences, Key Laboratory of Polymer Physics and Chemistry, Institute of Chemistry, Chinese Academy of Sciences, 100190 Beijing, P. R. China; 2 University of Chinese Academy of Sciences Beijing, 100049 Beijing, P. R. China; 3 State Key Laboratory of Organic-Inorganic Composites; Beijing Laboratory of Biomedical Materials, Beijing University of Chemical Technology, 100029 Beijing, P.R. China; 4 College of Life Science and Technology and State Key Laboratory of Organic-Inorganic Composites, Beijing University of Chemical Technology, 100029 Beijing, China; 5 Faculty of Chemistry and Biochemistry, Ruhr-University Bochum, Universitätsstrasse 150, Bochum, 44780 Germany

**Keywords:** Metastasis, Nanomedicine, Cancer therapy

## Abstract

Tumor metastases are considered the leading cause of cancer-associated deaths. While clinically applied drugs have demonstrated to efficiently remove the primary tumor, metastases remain poorly accessible. To overcome this limitation, herein, the development of a theranostic nanomaterial by incorporating a chromophore for imaging and a photosensitizer for treatment of metastatic tumor sites is presented. The mechanism of action reveals that the nanoparticles are able to intervene by local generation of cellular damage through photodynamic therapy as well as by systemic induction of an immune response by immunotherapy upon inhibition of the mTOR signaling pathway which is of crucial importance for tumor onset, progression and metastatic spreading. The nanomaterial is able to strongly reduce the volume of the primary tumor as well as eradicates tumor metastases in a metastatic breast cancer and a multi-drug resistant patient-derived hepatocellular carcinoma models in female mice.

## Abstract (web summary)

Approaches to combine photodynamic therapy (PDT) with immunotherapy are emerging, allowing the treatment of primary and metastatic tumors. Here the authors report the design and characterization of a nanomaterial for theranostic imaging and photodynamic therapy eliciting anti-tumor immune response in preclinical cancer models.

## Introduction

Tumor metastases are accountable for approximately 90% of all cancer-associated deaths^1,2^. Studies have shown that metastases occur when cancer cells stemming from the primary tumor invade the surrounding tissue and then enter the microvasculature of the lymph and blood system. The cancer cells can then be transported through the bloodstream to a distant site where the cells colonize and form secondary or metastatic tumors^3,4^. Over the last decades, traditional treatment modalities have been optimized to eradicate primary tumors. Despite recent improvements in chemotherapy, radiotherapy, and immunotherapy, clinical treatments of metastatic tumors remain highly challenging^5,6^. This is attributed to the diffuse spreading of the cancer cells towards various organs in the human body as well as the genetic and epigenetic modification of the cancer cells, causing drug resistance^7^. Among other approaches, increasing research interest has been devoted to the use of nanomaterials to identify or treat tumor metastases. Particles in the nanoscale are intrinsically associated with tumor-targeting properties based on their ability to accumulate in leaky, highly permeable vasculature and poor lymphatic tissue characteristics also referred to as the enhanced permeability and retention (EPR) effect^8–11^. Capitalizing on this, nanoparticle formulations of Doxorubicin (Doxil, Caelyx, and Myocet), Paclitaxel (Abraxane, Genexol PM), and Irinotecan (Onivyde) have been clinically approved for the treatment of metastatic tumors^12,13^.

As an emerging medicinal method for tumor treatment, increasing attention has been devoted to photodynamic therapy (PDT). During this therapeutic method, the patient is locally or systemically injected with a photosensitizer and after a certain incubation time the tumor site is exposed to irradiation. Upon light activation, the photosensitizer is able to photo-catalytically generate reactive oxygen species (ROS) and cause oxidative damage to the surrounding tissue, presenting a localized tumor treatment^14–19^. Despite its clinical application, the clinically approved photosensitizers, which are based on tetrapyrrolic compounds (i.e., porphyrin, chlorin, bacteriochlorin, phthalocyanine), are associated with similar drawbacks including poor cancer selectivity, poor water solubility, and poor (photo-)stability^20–23^. Capitalizing on this, research efforts have been devoted to the modification of the ligand scaffold. Among other promising compounds, dipyrrometheneboron difluoride (BODIPY) complexes have emerged based on their strong photophysical properties and high (photo-)stability in a biological environment^24–28^. Theoretical and experimental studies have indicated that the functionalization of the BODIPY complex with iodine atoms can promote the intersystem crossing process due to the heavy atom effect, yielding a higher ROS production^29^. To enhance the water solubility and provide cancer selectivity, research efforts have been devoted to the incorporation of photosensitizers into nanomaterials^30–37^.

Despite the strong therapeutic response of PDT agents upon irradiation, the treatment is localized towards the previously identified tumor site, preventing the treatment of metastatic tumors. To overcome this limitation, over the last years, the combination of PDT with immunotherapy has been proposed. During this multimodal treatment, the therapeutic agent is able to generate cytotoxic species causing localized cell damage as well as trigger a systemic immune response of the organism through immunogenic cell death (ICD) which could present useful in the treatment of tumor metastases^38–45^. Recent studies have indicated that the light activated PDT treatment is able to alter the cellular, stromal, and/or vascular properties of the tumor microenvironment, a process that is termed as photodynamic priming. These influences can make the tumorous tissue more susceptible for additional chemo- or immunotherapeutic treatments^46,47^. Based on this, research efforts have been focused on the discovery of compounds with a combined PDT and immunotherapeutic profile. Previous studies have indicated that the induction of an immune response inside a body could trigger severe side effects^38–40^. Capitalizing on this, there is a high demand for tumor-targeted therapeutic strategies.

For an effective PDT treatment, various parameters including the localization of (secondary) tumor sites, localization and concentration of the photosensitizer, necessary light dose, oxygen concentration, and heterogeneity of the tumor microenvironment need to be considered. Modern imaging techniques are able to provide this information through various methods. Alternatively, many research efforts have been invested in the development of multi-functional theranostic systems for combined cancer diagnosis and therapy. Based on the unique ability of nanomaterials to encapsulate therapeutic agents, nanoparticles have received much attention as multifunctional chromophore and drug carriers^48–52^.

**Fig. 1:**
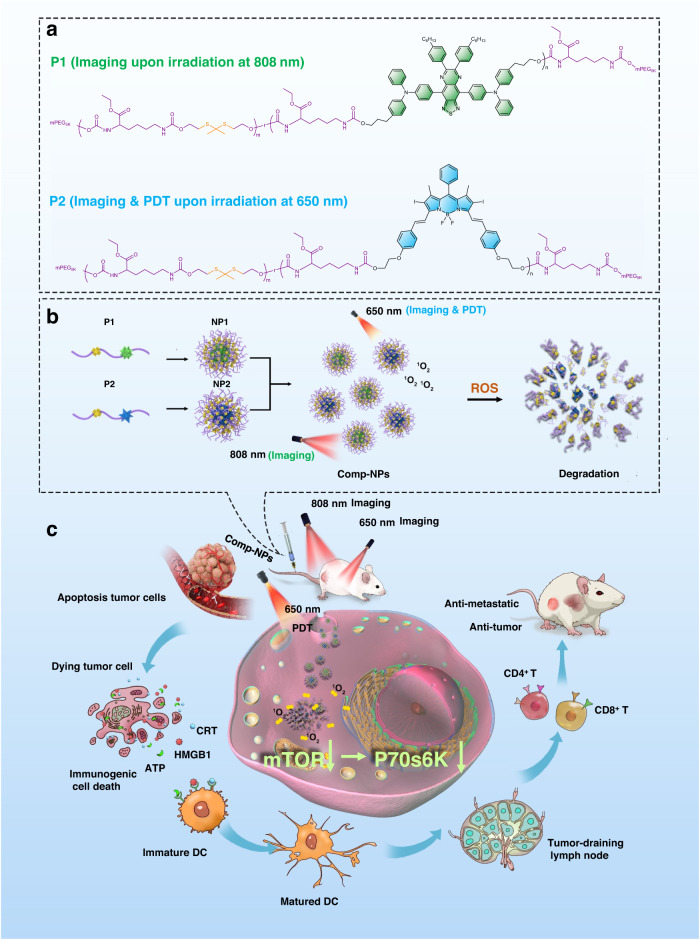
Structures and mechanism of action of Comp-NPs for the diagnosis by imaging and treatment of tumors by multimodal photodynamic therapy and immunotherapy. **a** Chemical structures of a polymer incorporating a chromophore for imaging upon irradiation at 808 nm (P1) or a photosensitizer for PDT upon irradiation at 650 nm (P2). **b** Self-assembly of the polymers into the nanoparticles NP1 and NP2. The theranostic nanoparticle formulation Comp-NPs is generated by mixing NP1 and NP2. **c** Biological mechanism of action of Comp-NPs by combined photodynamic therapy and immunotherapy.

In this work, the preparation, characterization, and in-depth biological evaluation of nanoparticles for theranostic imaging and treatment of tumors by multimodal photodynamic therapy and immunotherapy are presented. The previously reported and highly efficient chromophore 4,4′-(6,7-*bis*(4-hexylphenyl)-[1,2,5]thiadiazolo[3,4-g]quinoxaline-4,9-diyl)*bis*(*N*,*N*-diphenylaniline) (M1)^53^ and the photosensitizer 2,2′-((((1*E*,1′*E*)-(5,5-difluoro-2,8-diiodo-1,9-dimethyl-10-phenyl-5*H*-4λ^4^,5λ^4^-dipyrrolo[1,2-*c*:2′,1′-*f*][1,3,2]diazaborinine-3,7-diyl)bis(ethene-2,1-diyl))*bis*(4,1-phenylene))*bis*(oxy))bis(ethan-1-ol) (M2)^54^ are structurally modified through functionalization with hydroxy groups, allowing for the incorporation into a polymer backbone through polyurethane formation (Fig. 1a). To provide a higher therapeutic efficiency and ensure the excretion of the material after the treatment, thioacetal moieties, that readily dissociates in the presence of ROS^55^, are included in the polymer. Based on the amphiphilic nature of the polymers, these self-assemble in an aqueous solution into nanoparticles. For a theranostic application, the two nanomaterials are equimolar mixed and the composite nanoparticles (Comp-NPs) are formed (Fig. 1b). Insights into the mechanism of action reveal that the nanoparticles are able to intervene combined by the local generation of cellular damage through photodynamic therapy as well as by the systemic induction of an immune response through immunotherapy. Based on the intrinsic ability to the nanoparticles to accumulate at tumor sites as well as the necessity to be activated by light irradiation, this treatment is associated with a double selectivity. Proteomics analyses indicate that the nanoparticles reduce the mTOR signaling pathway, which is involved in tumor evolution and reoccurrence, and in the development of metastases (Fig. 1c). The nanomaterial demonstrates to reduce the volume of the primary tumor as well as to prevent the development of tumor metastases in a metastatic breast cancer mouse model in female mice.

## Results

### Preparation and characterization

**Fig. 2:**
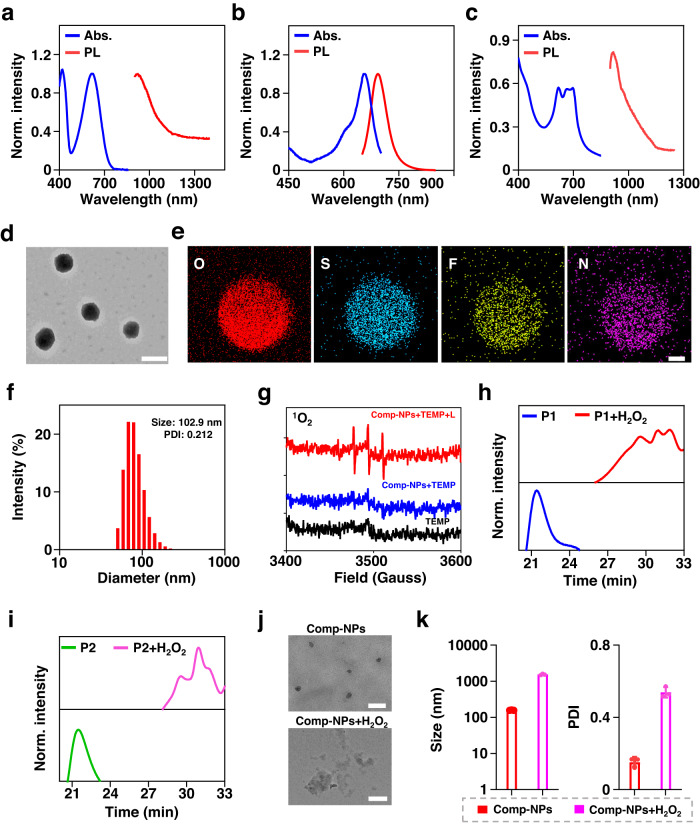
Physical and photophysical characterization of Comp-NPs. **a**–**c** Absorption and phosphorescence spectra of M1 in DCM (**a**, *λ*_ex_ = 650 nm), M2 in DCM (b, *λ*_ex_ = 808 nm) and Comp-NPs in phosphate-buffered saline (**c**, *λ*_ex_ = 808 nm) (*n* = 3 independent samples). **d** Representative transmission electron microscope image of Comp-NPs. (*n* = 3 independent samples). The experiment was repeated independently 3 times with similar results. scale bar = 200 nm. **e** Representative scanning transmission electron microscopy coupled with energy-dispersive X-ray spectroscopy images of Comp-NPs for oxygen, sulfur, fluorine, and nitrogen. scale bar = 50 nm. (*n* = 3 independent samples). **f** Size distribution of Comp-NPs in water determined by dynamic light scattering measurements. **g** Electron paramagnetic resonance spectra of Comp-NPs with or without laser irradiation (wavelength: 650 nm, power: 0.1 W cm^−2^, 60 J cm^−2^ time: 10 min) (*n* = 3 independent samples). **h**, **i** Gel permeation chromatography (GPC) measurements of P1 or P2 upon the addition of H_2_O_2_. (*n* = 3 independent samples). **j** TEM images of Comp-NPs and upon addition of H_2_O_2_. **k** Average particle size (left) and particle size distribution (right) of Comp-NPs in the presence or absence of 10 mM H_2_O_2_ measured by dynamic light scattering (*n* = 3 independent samples). scale bar = 200 nm. Error bars represent mean ± SD. Source data are provided as a Source Data file.

The phosphorescent monomer (M1) for imaging upon irradiation at 808 nm and photosensitizer monomer (M2) for imaging and photodynamic therapy upon irradiation at 650 nm were independently synthesized (synthetic scheme Supplementary Figs. 1, 2). The compounds were characterized by ^1^H-NMR and ^13^C-NMR spectroscopy as well as mass spectrometry (Supplementary Figs. 3–15). M1 was found with a maximum absorption centered at 645 nm and phosphorescence in the NIR-II region upon irradiation at 808 nm (Fig. 2a). M2 showed a maximum absorption centered at 650 nm and phosphorescence in the NIR-I region centered at 735 nm upon irradiation at 650 nm (Fig. 2b). The monomers M1/M2, l-lysine diisocyanate (LDI), and the ROS sensitive linker 2,2′-(propane-2,2-diylbis(sulfanediyl))*bis*(ethan-1-ol) (DSB) were mixed to generate a polymeric material. The hydroxy groups of M1/M2 and DSB can readily react with the isocyanate groups of LDI, resulting in a linkage of the respective moieties through LDI in the polymer. It is important to mention that M1/M2 and DSB are randomly distributed within the polymeric material. The terminal ends were functionalized with polyethylene glycol to generate the polymers P1 and P2 (Supplementary Figs. 16, 17). Using gel permeation chromatography, P1 was characterized with an average molecular weight of approximately 24,000 (*M*_*z*_/*M*_*w*_ = 1.55) and P2 of 30,000 (*M*_*z*_/*M*_*w*_ = 1.89). Using ^1^H-NMR spectroscopy, P1 was found with an approximate average of 28 M1-LDI units and 1.5 DSB-LDI units and P2 was found with an approximate average of 40 M2-LDI units and 2.0 DSB-LDI units. Based on the amphiphilic nature of the polymers, these self-assembled in an aqueous solution into the nanoparticles NP1 and NP2. Notably, the composition of NP1 and NP2 only differs in the use of a therapeutic or diagnostic agent in the lipophilic region of the polymeric chain. As the lipophilic parts of the polymers are in the center of the nanoparticles and the hydrophilic regions of the polymeric chain are found on the outer surface of the nanoparticles, it is expected that NP1 and NP2 have similar pharmacological properties. The absorption spectra of the nanoparticles showed transitions in the same range as the polymers, indicating the preservation of the photophysical properties (Supplementary Fig. 18). For theranostic applications, NP1 and NP2 were mixed in a 1:1 ratio and the theranostic nanoparticle formulation Comp-NPs was generated. It is important to highlight that Comp-NPs are not specific nanoparticles, but instead a drug formulation containing an equimolar mixture of NP1 and NP2. As expected, Comp-NPs showed absorption peaks of NP1 and NP2 and were found to be highly phosphorescent in the NIR-II region (Fig. 2c). The emissive properties of NP1 and NP2 were individually assessed and compared with Comp-NPs. The results showed that Comp-NPs had the exact additive emission properties of the individual nanoparticles, ruling out the presence of fluorescence resonance energy transfer processes associated processes. The luminescence quantum yield of Comp-NPs was determined to be 2.0% upon excitation at 808 nm. Transmission electron microscope images of NP1, NP2, and Comp-NPs demonstrated the spherical morphology of the nanomaterials (Supplementary Fig 19, Fig. 2d). Elemental mapping of the nanoparticles verified the uniform distribution of oxygen, sulfur, fluorine, and nitrogen in the nanoparticles (Fig. 2e). Using dynamic light scattering measurements, the hydrodynamic diameters of the nanoparticles NP1, NP2 and Comp-NPs were found to be in the range of 90–100 nm (Supplementary Table 1, Fig. 2f), which is considered ideal for drug delivery.

The identity of the generated ROS by Comp-NPs upon exposure to light was studied using electron paramagnetic resonance spectroscopy with 2,2,6,6-tetramethylpiperidine as a singlet oxygen (^1^O_2_) scavenger and 5,5-dimethyl-1-pyrroline-N-oxide as a ^•^OOH or ^•^OH radical scavenger. While no signal for the generation of ^•^OOH or ^•^OH radical species was observed, the characteristic ^1^O_2_-induced triplet signal for 2,2,6,6-tetramethylpiperidinyloxyl was measured (Fig. 2g). For a deeper insight into the ability of Comp-NPs to generate ^1^O_2_ upon irradiation, its production was time-dependently monitored by absorption spectroscopy using 1,3-diphenylisobenzofuran (DPBF) as a ^1^O_2_ specific probe. Upon irradiation for 10 min, the absorption of DPBF decreased from approximately 0.9 to 0.4 (Supplementary Fig 20), indicating the efficient generation of ^1^O_2_.

In order to study the ROS sensitivity, the polymers P1 and P2 were dissolved in dimethylformamide and exposed to hydrogen peroxide (H_2_O_2_) as a model for the production of ROS upon irradiation. While no changes in the average weight for P1 and P2 upon incubation in organic solvents were observed, a quick degradation was noticed upon the addition of H_2_O_2_ into a mixture of oligomers with lower molecular weights (1000–9000) (Fig. 2h, i). To study the stability of the nanoparticles, the size and morphology of NP1, NP2, and Comp-NPs were monitored by transmission electron microscopy (TEM). Upon the addition of H_2_O_2_, the degradation of the nanomaterials was observed (Fig. 2j and Supplementary Fig 19). Complementary, the dye Nile Red was encapsulated into Comp-NPs and the release of the chromophore was time-dependently monitored by phosphorescence spectroscopy. Nile Red is known to be highly luminescent in apolar environments such inside nanoparticles, but is quenched inside an aqueous solution^56^. While no changes in the spectra were observed upon incubation of Comp-NPs in the dark, quenching and therefore release of the dye from the nanomaterial was noticed upon irradiation of the solution (Supplementary Fig S21). Complementary, the decomposition of the nanoparticles upon exposure to H_2_O_2_ was further studied by dynamic light scattering measurements. In the presence of H_2_O_2_ the size distribution and the polydispersity were drastically enhanced (Fig. 2k) due to the release of small molecular therapeutic agents and the aggregation of the lipophilic hydrophobic fragments. Overall, these results indicate that Comp-NPs remains stable under physiological conditions but are quickly degraded in the presence of ROS or upon irradiation.

### Biological PDT effect in a 4T1 cancer cell model

**Fig. 3:**
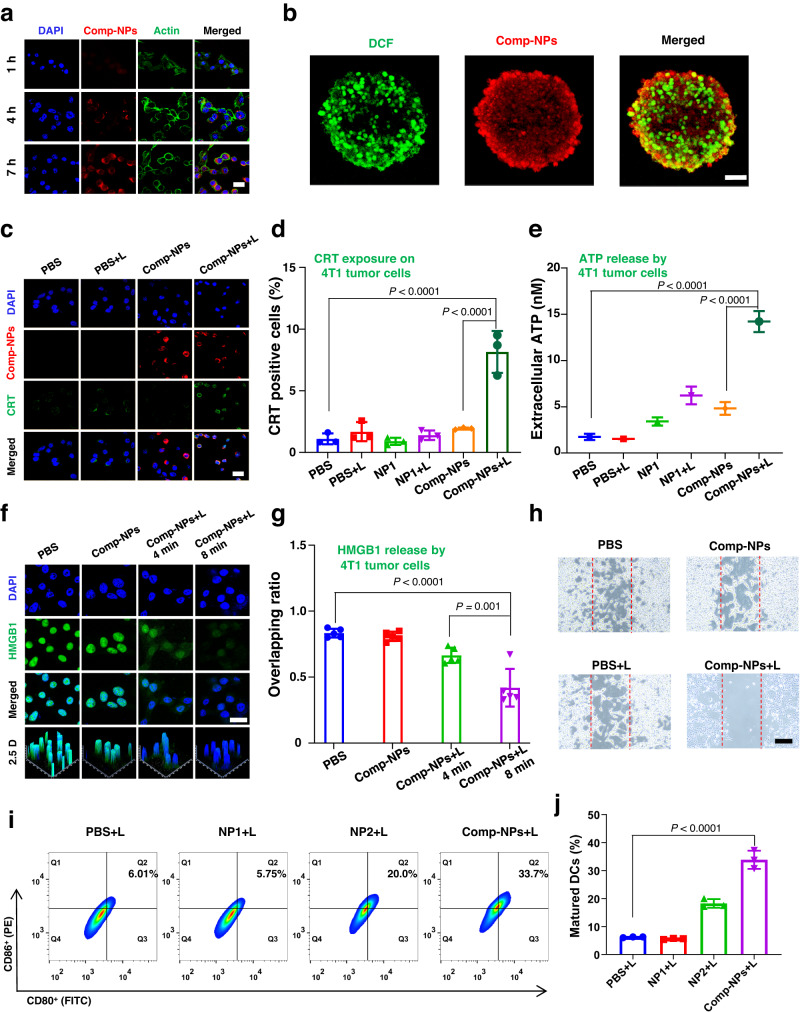
Cellular uptake, ROS generation, and cell death mechanism of Comp-NPs in a 4T1 monolayer cancer cell model or 4T1 multicellular tumor spheroids. **a** CLSM images of 4T1 cells incubated with Comp-NPs at 37°C for 1 h, 4 h and 7 h. The experiment was repeated independently 3 times with similar results. scale bar = 10 μm. **b** CLSM images of 4T1 MCTS with a diameter of ~700 μm incubated with Comp-NPs at 37 °C for 7 h and the ROS specific probe for 30 min, followed by exposure to irradiation (650 nm, 0.1 W cm^−2^, 30 J cm^−2^, 5 min). The experiment was repeated independently 3 times with similar results. scale bar = 100 μm. **c** CLSM images of 4T1 cells incubated with the CRT fluorescent probe (CRT, green) and DAPI (blue) upon various treatments in the light (650 nm, 0.1 W cm^−2^, 2 min). scale bar = 20 μm. **d**, **e** Quantification of the translocation of CRT to the cell surface of 4T1 cells and quantification of extracellular adenosine triphosphate (ATP)upon various treatments in the light (650 nm, 0.1 W cm^−2^, 2 min) by flow cytometry (*n* = 3 biologically independent samples). **f** CLSM images of 4T1 cells incubated with the HMGB1 protein fluorescent probe (HMGB1, green) and DAPI (blue) upon various treatments in the light (650 nm, 0.1 W cm^−2^, 2 min). scale bar = 20 μm. **g** Quantification of the release of the HMGB1 protein from (**f**) (*n* = 5 biologically independent cells). **h** Cell migration wound healing assay of 4T1 cells upon various treatments in the light (650 nm, 0.1 W cm^−2^, 30 J cm^−2^, 5 min). The experiment was repeated independently 3 times with similar results. scale bar = 50 μm. **i**, **j** Quantification of the maturation of DCs upon various treatments in the light (650 nm, 0.1 W cm^−2^, 30 J cm^−2^, 5 min) (*n* = 3 biologically independent samples). Statistical analysis was performed by one-way ANOVA with Tukey’s multiple comparisons test. All data are presented as mean ± SD. Source data are provided as a Source Data file.

The biological properties of Comp-NPs were in-depth studied in mouse breast cancer (4T1) cells. As a crucial property for a biological effect, the cellular uptake was investigated upon time-dependent monitoring of the phosphorescence of the nanoparticles inside the cancer cells by confocal laser scanning microscopy (CLSM). With the prolongation of the incubation time, an increasing amount of phosphorescence was detected inside the cancer cells, indicating the efficient and time-dependent cellular uptake of Comp-NPs (Fig. 3a). Using flow cytometry, the cellular uptake was verified (Supplementary Fig 22a-b). Followingly, the uptake of the nanoparticles was investigated in multicellular tumor spheroids (MCTS) as a tissue culture model for the delivery of compounds into multicellular architectures. This is in particular important as many anticancer agents have failed the translation from a monolayer cancer cell model to animal models due to compromised drug delivery^57,58^. Therefore, herein, 4T1 MCTS with a diameter of ~700 μm were used as a mimic to study the penetration of three-dimensional cellular architectures. Upon incubation of Comp-NPs for 7 h, z-stack CLSM showed a strong red phosphorescence signal at every section depth (Supplementary Fig 22c), indicative of the complete penetration of the MCTS.

The ability of the nanoparticles to generate ROS inside the MCTS was studied using the ROS specific probe 2′,7′-dichlorofluorescein diacetate. While the probe is non-luminescent in a biological milieu, in the presence of ROS it is oxidized and the fluorescent 2′,7′-dichlorofluorescein product is formed. While no green fluorescence of the probe was observed upon incubation of the MCTS with the nanoparticles in the dark, a strong green fluorescence signal upon exposure of the MCTS to light was observed, suggesting the production of ROS (Fig. 3b). Using flow cytometry, the ROS production inside the cells was verified (Supplementary Fig 23).

The cytotoxic effect of the nanoparticles NP1, NP2, and Comp-NPs towards murine breast 4T1 cancer cells, human ovarian cancer (SKOV3), cisplatin sensitive ovarian cancer (A2780), cisplatin-resistant ovarian cancer (A2780DDP) and human hepatocellular carcinoma (JHH7) cells was investigated in the dark as well as upon irradiation (650 nm, 0.1 W cm^−2^, 60 J cm^−2^, 10 min). All nanoparticle formulations were found to be non-toxic in the dark, which is an important requirement for an imaging and PDT agent (IC_50,dark_ > 6 μg/mL BODIPY). While imaging upon irradiation at 808 nm nanoparticles NP1 did not show a cytotoxic effect upon exposure to light, a strong phototoxic effect for NP2 (IC_50,light_ = 0.46 ± 0.13 μg/mL) and Comp-NPs (IC_50,light_ = 0.47 ± 0.03 μg/mL BODIPY, Supplementary Table 2, Supplementary Fig 24) was observed. Remarkably, Comp-NPs showed to eradicate 80% of the cancer cell population at a concentration of 0.5 μg/mL BODIPY upon exposure to light. For a deeper insight into the biological properties, nanoformulations of Comp-NPs with various ratios of NP1 and NP2 (1:2, 1:3, 2:1, 3:1) were prepared and their cytotoxicity against 4T1 cells was assessed. Using an equal amount of the photosensitizer BODIPY, a cytotoxic effect inside the same range was observed (IC_50,light_ = 0.38 – 0.76 μg/mL BODIPY) (Supplementary Table 3, Supplementary Fig 25). These findings indicate that the ratio between NP1 and NP2 could be fine-tuned in future studies to generate only the desired therapeutic response.

For a deeper understanding of the phototoxic effect, the cell death mechanism of the nanoparticles was studied. The ability to trigger apoptotic cell death was investigated by flow cytometry using the dual probes FITC-Annexin V/Propidium Iodide. While no cellular death upon treatment of Comp-NPs in the dark was observed, the majority of the cancer cell population showed apoptotic cell death upon exposure of the cells to light (Supplementary Fig 26). Western Blot analysis indicated that the treatment with Comp-NPs and exposure to light triggered the activation of the expression of caspase-3 (Supplementary Fig 27), indicative that cell death is induced by apoptosis using the caspase-3 pathway. Using light microscopy, the shrinkage of the cancer cells and the fragmentation into membrane-bound apoptotic bodies is observed upon treatment with Comp-NPs and exposure to light as this is typically observed for apoptotic death cell (Supplementary Fig 28). Recent studies have indicated that apoptotic dying cells could release damage-associated molecular patterns (DAMPs) which are able to trigger an immune response inside the organism^59,60^. Capitalizing on this, the cancer cells were analyzed upon treatment towards specific hallmarks for immunogenic cell death (ICD). Using CLSM, the translocation of the endoplasmic reticulum resident calreticulin (CRT) to the cell surface upon treatment with Comp-NPs in the light was measured (Fig. 3c), supporting the interaction of macrophages for tumor antigen presentation. This effect was further quantified using flow cytometry (Fig. 3d). The secretion of adenosine triphosphate (ATP) was studied which promotes the attraction of tumor-specific T-cells. The results showed a highly increased amount of ATP upon treatment with Comp-NPs and exposure to light (Fig. 3e). The release of the nuclear high-mobility group box 1 (HMGB1) protein, which triggers the myeloid differentiation signaling cascade required for antigen presentation to T-cells, was observed by CLSM using a specific nuclear HMGB1 protein chromophore (Fig. 3f-g). Complementary, the migration of the nuclear HMGB1 protein was observed by immunofluorescence CLSM (Supplementary Fig 29). Combined these results indicate that Comp-NPs is able to efficiently trigger cell death combined by apoptosis and ICD. The ability to trigger an immune response could allow for the treatment of distant tumor site or metastatic tumors. As a preliminary model, a cell migration wound healing assay was performed. The incubation with Comp-NPs in the light showed the least amount of cell migration (Fig. 3h and Supplementary Fig 30), indicative of the ability to inhibit cell migration and treat distant cancer cells. For a deeper insight into the activation of the immune response, 4T1 cells were treated with NP1, NP2 or Comp-NPs in the dark as well as upon irradiation and afterwards dendritic cells (DC2.4) were added. Using flow cytometry, the maturation of the DCs was assessed. While no significant immune response was observed during the treatment in the dark, strong activation of the immune system (up to ~34% of DC maturation) was monitored upon incubation with Comp-NPs and exposure to irradiation (Treatment in the dark: Supplementary Fig 31, Treatment in the light: Fig. 3i-j). Overall, these results demonstrated the ability of Comp-NPs to be efficiently taken up by cancer cells, generate ROS, and cause a strong cytotoxic effect upon irradiation by a combination of apoptosis and ICD.

### Biological imaging and PDT effect in a 4T1 tumor-bearing mouse model

**Fig. 4:**
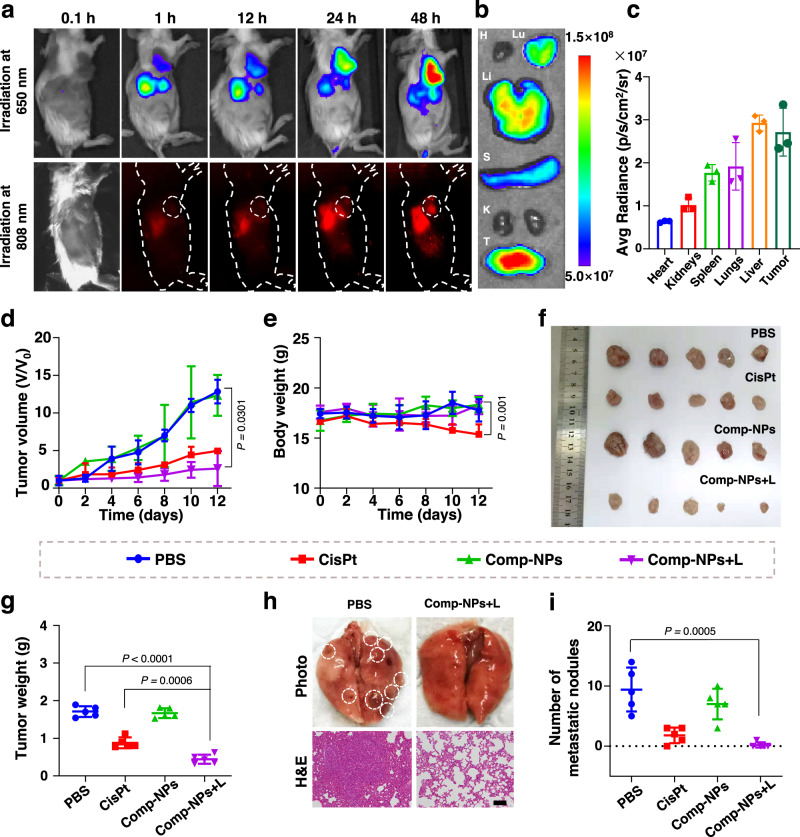
Imaging and PDT properties of Comp-NPs were evaluated in a 4T1 tumor-bearing mouse model. **a** Biodistribution of Comp-NPs inside the living animal model determined upon irradiation at 808 nm and 650 nm. **b** Phosphorescence images of the major organs and the tumor after the sacrifice of the mouse model which was 48 h before intravenously injected in the tail vein with Comp-NPs. *λ*_ex_ = 650 nm, *λ*_em_ = 735 nm. **c** Quantification of the accumulation of Comp-NPs from (**b**) (*n* = 3 mice). Error bars represent mean ± SD. **d** Tumor growth inhibition curves (*n* = 5 mice) upon treatments with Comp-NPs (6 mg BODIPY kg^−1^) or cisplatin (0.5 mg Pt kg^−1^) in the dark or upon irradiation (650 nm, 0.1 W cm^−2^, 60 J cm^−2^, 10 min). Error bars represent mean ± SD. Statistical analysis was performed by two-tailed unpaired *t* test. **e** Body weight of the mice (*n* = 5 mice) upon various treatments with Comp-NPs (6 mg BODIPY kg^−1^) or cisplatin (0.5 mg Pt kg^−1^) in the dark or upon irradiation (650 nm, 0.1 W cm^−2^, 60 J cm^−2^, 10 min). Error bars represent mean ± SD. Statistical analysis was performed by one-way ANOVA with a Tukey’s multiple comparisons test. **f** Photographs of the tumors after various treatments. (*n* = 5 mice). **g** Weight of the tumors from (**f**) (*n* = 5 mice). Error bars represent mean ± SD. Statistical analysis was performed by one-way ANOVA with Tukey’s multiple comparisons test. **h** Photographs of metastatic nodules (highlighted white circles) in the lungs and H&E stain of tissue slices of this organ. scale bar = 100 μm. **i** Quantification of the amount of metastatic lung nodules after various treatments (*n* = 5 mice). Error bars represent mean ± SD. Statistical analysis was performed by two-tailed unpaired *t* test as a Source Data file.

Based on the promising properties in a cancer cell model, the imaging and therapeutic PDT effects were further studied inside a 4T1 tumor-bearing mouse model. For an evaluation of the biosafety of the nanoparticles, Comp-NPs were intravenously injected into the tail vein of the animal model and the behavior of the mouse was monitored. As no signs of stress, discomfort, or change in weight of the animal were observed over a period of two weeks after the injection, the high biocompatibility of the nanomaterial is indicated. The biodistribution of the nanoparticles inside the living mouse model was studied upon NIR-I (*λ*_ex_ = 650 nm, *λ*_em_ = 745 nm) and NIR-II (*λ*_ex_ = 808 nm, *λ*_em_ = 950 nm) phosphorescence imaging. With the prolongation of the circulation time an increasing amount of Comp-NPs accumulated at the tumor site, suggesting tumor-targeting properties of the nanomaterial (Fig. 4a). For quantification, organs as well as the tumor were collected, and the biodistribution was determined by phosphorescence imaging of the respective tissues. The results showed that Comp-NPs were found with the highest accumulation inside the liver, as this is typically the case for nanomaterials, and the tumor (Fig. 4b-c). These findings indicate the potential application of Comp-NPs as an imaging agent for the identification of possible tumor sites.

The therapeutic PDT effect inside 4T1 tumor-bearing mice was studied upon intravenous injection of Comp-NPs (6 mg BODIPY kg^−1^) into the tail vein and monitoring of changes in the tumor volume upon treatment in the dark or exposure to irradiation (650 nm, 0.1 W cm^−2^, 60 J cm^−2^, 10 min). Promisingly, upon injection of the animal model with Comp-NPs and exposure to light, a strong tumor growth inhibition effect was observed within a single treatment. The tumor remission was found to be slightly but not statically significantly stronger than upon treatment with the anticancer drug cisplatin (Fig. 4d). While the body weight of the animals treated with the nanoparticles remained unchanged, a significant reduction of the body weight upon treatment with cisplatin was observed (Fig. 4e), indicative for severe side effects. As key parameters for the biosafety of the treatment, the levels of ALT, AST, BUN and CR inside the animal model were analyzed. While no changes in the biochemical levels of all parameters during the treatment with Comp-NPs were observed, a strong increase of the BUN and CR factor for the treatment with cisplatin was observed, verifying the toxicity of cisplatin (Supplementary Fig 32a). Complementary, the major organs of the animals treated with Comp-NPs in the dark or upon irradiation did not show any pathological alternations in a hematoxylin and eosin (H&E) stain of the respective tissues (Supplementary Fig 32b). After the respective treatments, the tumors were collected and weighted (Fig. 4f: Photographs of the tumor, Fig. 4g: Tumor weight). While the mice injected with saline showed an average tumor weight of ~1.7 g, the animals treated with Comp-NPs and exposed to light were found with an average tumor weight of ~0.4 g, highlighting the strong tumor remission. Based on the metastatic nature of 4T1 breast cancer tumors^61^, various metastases were observed in the lungs of the untreated animal model. It is important to mention that metastases are considered the leading cause of (breast) cancer-associated human deaths^62^. Strikingly, the mice treated with Comp-NPs and exposed to irradiation did not show any signs of metastases (Fig. 4h-i), indicating the ability to prevent the development of metastases. Overall, these findings suggest that Comp-NPs is able to act combined as a NIR-I and NIR-II imaging agent for identification of tumor sites, PDT agent with a strong tumor remission within a single dose and light treatment, and anti-metastatic agent to prevent the development of metastases at distant tissues in the animal model.

### Immunogenic PDT effect in a (Metastatic) 4T1 tumor-bearing mouse model

**Fig. 5:**
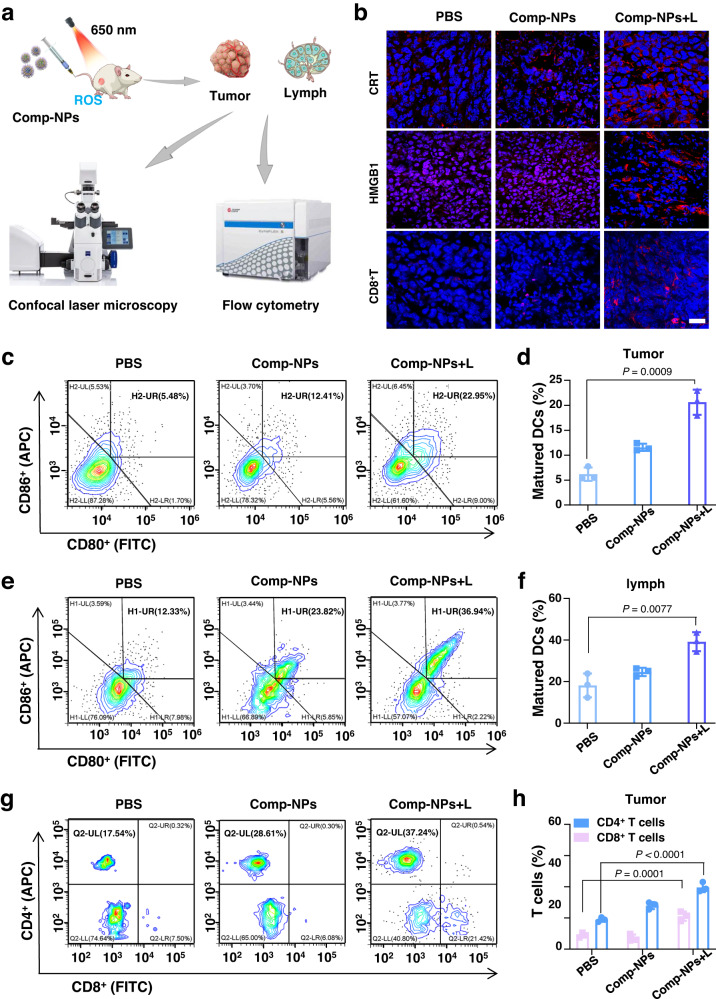
Mechanism of the immunogenic PDT response inside a 4T1 tumor-bearing mouse model upon treatment with Comp-NPs and exposure to irradiation. **a** Schematic diagram of immune response test after Comp-NPs upon the 650 nm laser. **b** CLSM images of tumor slices obtained from variously treated 4T1 mouse xenograft models incubated with DAPI (blue, *λ*_ex_ = 410 nm, *λ*_em_ = 506 nm) and top: CRT specific fluorescent probe (CRT, red, *λ*_ex_ = 488 nm, *λ*_em_ = 525 nm), middle: HMGB1 protein fluorescent probe (HMGB1, red, *λ*_ex_ = 488 nm, *λ*_em_ = 525 nm), bottom: CD8^+^ T-cells fluorescent probe (red, *λ*_ex_ = 488 nm, *λ*_em_ = 525 nm). (The images are representative of 3 mice per group). The experiment was repeated independently 3 times with similar results. scale bar = 20 μm. **c**–**d** Analysis of the levels of activated dendritic cells (CD80^+^, CD86^+^) in tumor slices obtained from variously treated 4T1 mouse models (*n* = 3 mice). Error bars represent mean ± SD. Statistical analysis was performed by two-tailed unpaired *t* test. **e**, **f** Analysis of the levels of activated dendritic cells (CD80^+^, CD86^+^) in lymph nodes slices obtained from variously treated 4T1 mouse models. (*n* = 3 mice). Error bars represent mean ± SD. Statistical analysis was performed by two-tailed unpaired *t* test. **g**, **h** Analysis of the levels of effector T-cells (activated CD8^+^ T-cells, activated CD4^+^ T-cells) in tumor slices obtained from variously treated 4T1 mouse models. (*n* = 3 mice). Error bars represent mean ± SD. Statistical analysis was performed by two-way ANOVA with Šídák’s multiple comparisons test. Source data are provided as a Source Data file.

Capitalizing on the strong antimetastatic effect of the nanoparticles, the immune response inside the animal model was evaluated (Fig. 5a). Based on the ability of the nanomaterial to trigger ICD in monolayer cancer cells, this biological mechanism was investigated inside the animal model upon biochemical assessment of tissue slices of the tumorous tissue for specific hallmarks. Using a CRT-specific probe and immunofluorescence CLSM, the migration of CRT to the cell surface was observed as indicated by the red fluorescence in the microscopy images upon treatment with Comp-NPs and exposure to light (Fig. 5b top panel). Quantification of the fluorescence signal demonstrated that the treatment with the nanoparticles caused an approximately 8.3 times higher migration of CRT than the treatment with saline (Supplementary Fig 33a). Followingly, the localization of the HMGB1 protein was investigated by CLSM using a HMGB1 protein-specific probe. While the HMGB1 protein was primarily found inside the nucleus upon treatment with saline, as indicated by the congruency with the fluorescence signal of the nucleus stain DAPI, the tumors treated with Comp-NPs and exposure to irradiation showed the migration of the HMGB1 protein (Fig. 5b middle panel). Quantification of the ratio of the fluorescence of the HMGB1 protein with the fluorescence of nuclear stain DAPI showed that the treatment with Comp-NPs and exposure to irradiation resulted in the migration of the HMGB1 protein of approximately 40% from the cell nucleus (Supplementary Fig 33b). Based on these hallmarks for an immune response of the organism, the levels of CD8^+^ T-cells were assessed by CLSM using a CD8^+^ T-cells specific fluorescent probe. While only a minimal amount of CD8^+^ T-cells were noticed in the microscopy images during the treatment with saline, a significant amount of immune CD8^+^ T-cells were observed upon treatment with Comp-NPs and exposure to light as indicated by the red fluorescence signal (Fig. 5b bottom panel). A comparison of the fluorescence signals suggested that an approximately 5 times higher amount of CD8^+^ T-cells was found in the tissue treated with Comp-NPs and exposure to light than in the control group (Supplementary Fig 33c).

The ability of the nanoparticles to stimulate and promote the systemic host immune response was studied upon the determination of the levels of T-cells and matured DC cells. During ICD, DAMPs are released which are able to promote the transmission of phagocytic signals to DC-based antigen-presenting cells and therefore enhance the immune response of the organism. The treatment with Comp-NPs and exposure to irradiation demonstrated to enhance the population of matured DC cells (CD80^+^, CD86^+^) up to approximately 21%, corresponding to an approximate 3 times augmentation of the levels found in the animal models (Fig. 5c, d). The proportion of matured DC cells in the lymph nodes was increased to approximately 39%, indicating a doubling of the amount of DC cells in this organ (Fig. 5e, f). In agreement with this finding, the proportion of T-cells (CD4^+^, CD8^+^) in the tumor tissues approximately also doubled up to 40% (Fig. 5g, h). Overall, these results demonstrate the immunogenic effect upon PDT treatment with Comp-NPs and rationalize the strong tumor remission as well as inhibition of the development of tumor metastases.

To provide a deeper understanding, the tumor growth inhibition effect as well as induction of the immune response of Comp-NPs was compared to the clinically approved ICD inducing anticancer drug oxaliplatin inside a 4T1 tumor-bearing mouse model. Comp-NPs and oxaliplatin were injected into the tail vein and the changes in the tumor volume upon treatment in the dark or exposure to irradiation (650 nm, 0.1 W cm^−2^, 60 J cm^−2^, 10 min) were monitored. The weight of the mice did not significantly change, indicative of the biocompatibility of the treatment. The tumor of the animals treated with Comp-NPs grew exponentially in a similar manner as the control group. The treatment with oxaliplatin showed a remission of the tumor. The tumor of the mice treated with Comp-NPs and exposed to irradiation was fully eradicated (Supplementary Fig 34a: Cumulative tumor growth inhibition curves, Fig S34b: Photographs of the animals after the treatment, Supplementary Fig 34c: Changes in body weight, Supplementary Fig 34d: Individual tumor growth inhibition curves). Using flow cytometry, the induction of the immune response upon treatment was quantified. The treatment with Comp-NPs in the dark was found not to elevate the levels of DCs inside the animal model. The treatment with Comp-NPs and exposure to irradiation demonstrated to enhance the population of matured DC cells (CD80^+^, CD86^+^) up to approximately 22%, corresponding to an approximately 2.2 times higher level than upon treatment with oxaliplatin (level of matured DC cells of ~10%) and approximately 4.4 times higher level than in the control group (level of matured DC cells of ~5%) (Supplementary Fig 34e, f). The proportion of T-cells (CD4^+^, CD8^+^) in the tumor tissue was found to be highly elevated upon treatment with oxaliplatin or Comp-NPs. The levels of CD4^+^ T-cells upon treatment with Comp-NPs reached approximately 49%, corresponding to an approximately 1.3 times higher level than upon treatment with oxaliplatin (level of CD4^+^ T-cells of ~39%) and approximately 2.9 times higher level than in the control group (CD4^+^ T-cells of ~17%). In agreement, the levels of CD8^+^ T-cells upon treatment with Comp-NPs reached approximately 31%, corresponding to an approximately 1.4 times higher level than upon treatment with oxaliplatin (level of CD4^+^ T-cells of ~22%) and approximately 2.8 times higher level than in the control group (CD4^+^ T-cells of ~11%) (Supplementary Fig 34g, h). The proportion of effector T lymphocyte interferon‐gamma IFN‐γ^+^CD8^+^ T cells inside the tumor was assessed. The treatment with Comp-NPs and exposure to irradiation demonstrated to enhance the population of IFN‐γ^+^CD8^+^ T cells up to approximately 16%, corresponding to an approximately 2.0 times higher level than upon treatment with oxaliplatin (level of matured DC cells of ~8%) and approximately 5.3 times higher level than in the control group (level of matured DC cells of ~3%) (Supplementary Fig 34i-j). Overall, these results suggest that the treatment with Comp-NPs showed a stronger tumor growth inhibition effect as well as stronger immune response than the treatment with the anticancer drugs oxaliplatin.

**Fig. 6:**
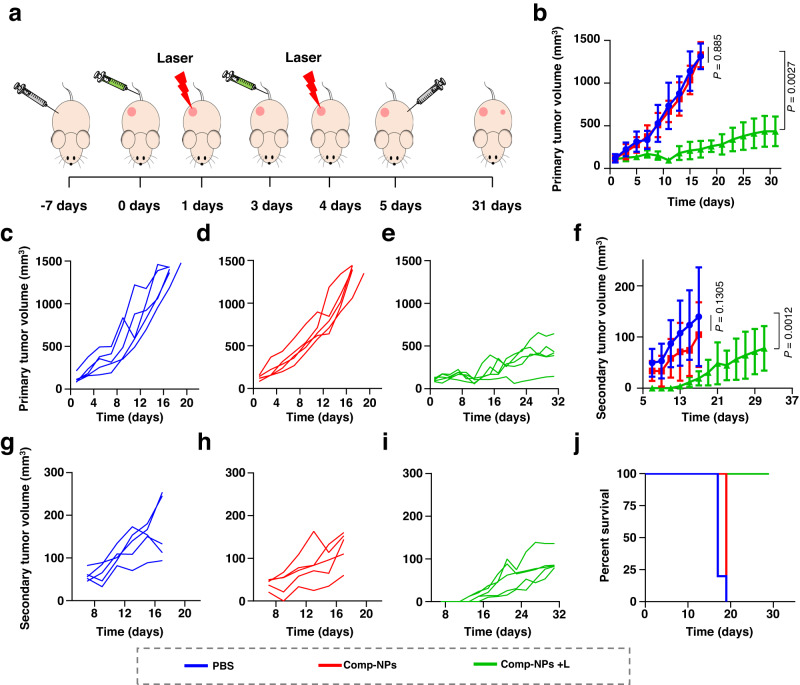
PDT properties of Comp-NPs evaluated in a mouse model bearing a primary and secondary distant tumor. **a** Schematic illustration of the establishment of a dual primary and secondary tumor mouse model, administration and treatment schedules with Comp-NPs (6 mg BODIPY kg^−1^) in the dark or upon irradiation (650 nm, 0.1 W cm^−2^, 60 J cm^−2^, 10 min). Phosphate-buffered saline (PBS) was used as a control. **b** Mean tumor growth inhibition curves (*n* = 5 mice) of the primary tumor upon treatment. Error bars represent mean ± SD. Statistical analysis was performed by two-tailed unpaired *t* test. **c**–**e** Individual tumor growth inhibition curves (*n* = 5 mice) of the primary tumor upon treatment. **f** Mean tumor growth inhibition curves (*n* = 5 mice) of the secondary tumor upon treatment. Error bars represent mean ± SD. Statistical analysis was performed by two-tailed unpaired *t* test. **g**–**i** Individual tumor growth inhibition curves of the secondary tumor upon treatment. **j** Long-term survival of the animal models (*n* = 5 mice) upon treatment. Source data are provided as a Source Data file.

The ability of the immunogenic response to treat distant tumors was studied in a mouse model with two separated 4T1 tumors. The primary tumor was grown on the right side of the animal model and the mouse twice treated by intravenous administration of Comp-NPs and exposure to light. Afterwards, cancer cells were injected on the left of the mouse to form a secondary tumor and the tumor growth monitored in both places (treatment schedule: Fig. 6a). In agreement with the previous assessment (Fig. 4), a strong primary tumor remission was observed upon administration of the nanoparticles and exposure to light (Fig. 6b–e). Strikingly, the growth of the secondary tumor was also strongly inhibited upon injection of Comp-NPs and exposure to light (Fig. 6f–i). Since the mice, which were injected with the second dose of cancerous cells, were kept in the dark and the nanoparticles were found not to influence the tumor growth in the dark, the remission of the secondary tumor is attributed to the immunogenic response in the animal model. The monitoring of the long-term survival indicated a strong enhancement of the life expectancy upon treatment. While the non-treated or in the dark treated animals died after 19–21 days, all mice models injected with Comp-NPs and exposed to irradiation were still alive after 30 days (Fig. 6j). These results strongly indicate the ability of the nanoparticles to eradicate distant/secondary tumor sites through an immunogenic response as well as enhance the survival of the animal model.

### Therapeutic PDT effect in patient-derived tumor-bearing mouse models

**Fig. 7:**
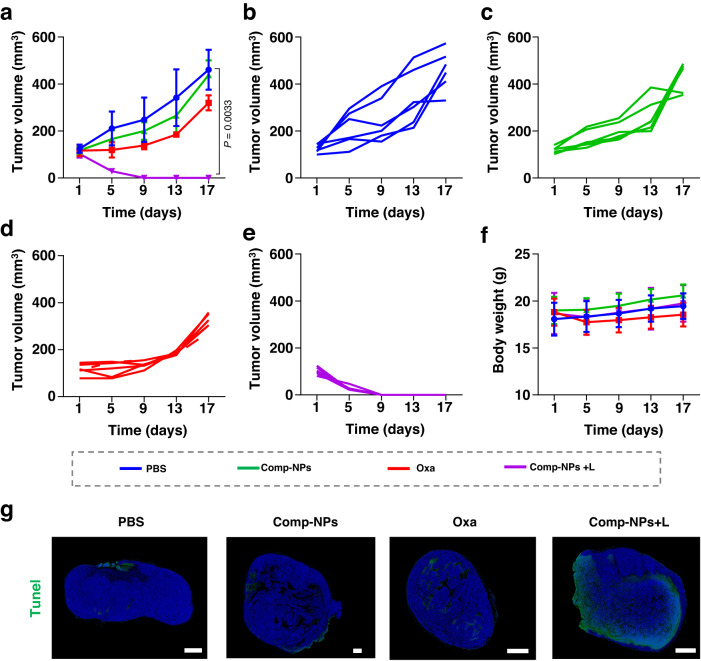
PDT properties of Comp-NPs (6 mg BODIPY kg^−1^) in comparison to clinically approved chemotherapeutic drug oxaliplatin (Oxa, 0.5 mg Pt kg^−1^) in the dark or upon irradiation (650 nm, 0.1 W cm^−2^, 60 J cm^−2^, 10 min) evaluated in a multi-drug resistant patient-derived breast carcinoma inside a mouse model. **a** Cumulative tumor growth inhibition curves (*n* = 6 mice) upon treatment. Error bars represent mean ± SD. Statistical analysis was performed by two-tailed unpaired *t* test. **b**–**e** Individual tumor growth inhibition curves (*n* = 6 mice) upon treatment. **f** Body weight of the mice (*n* = 6 mice) upon treatment. Error bars represent mean ± SD. **g** Terminal deoxynucleotidyl transferase dUTP nick end labeling (green) and DAPI (blue) stain of tumor tissue slices after various treatments (the images are representative of 6 mice per group). scale bar = 1 mm. Source data are provided as a Source Data file.

To assess the potential of the nanoparticles to treat clinically challenging tumors, the therapeutic properties of Comp-NPs towards multi-drug resistant patient-derived breast carcinoma tumors inside a mouse model were investigated. The cancer cells were obtained from a patient with reoccurring tumors despite multiple rounds of surgery and chemotherapy. The tumors of the animals treated with nanoparticles in the dark showed a similar growth as the control group. However, when the animals were injected with Comp-NPs and exposed to irradiation, the tumor was fully eradicated after a single treatment. Due to the drug resistance of the tumor to various chemotherapeutic drugs, the treatment with oxaliplatin showed only a minimal effect on the tumor remission. These findings are highly promising and suggest that Comp-NPs have the potential to effectively treat multi-drug resistant and clinically challenging tumors (Fig. 7a: Cumulative tumor growth inhibition curves, Supplementary Fig 35: Photographs of the animals after the treatment, Fig. 7b–e: Individual tumor growth inhibition curves). It is noteworthy that the animals treated with Comp-NPs did not exhibit any signs of pain, discomfort, or weight loss (Fig. 7f). For a deeper insight, the tumorous tissue was histologically analyzed. The tumor slices of the mice treated with Comp-NPs and exposed to irradiation showed cellular and tissue damage (Fig. 7g), indicative of the efficient PDT treatment.

**Fig. 8:**
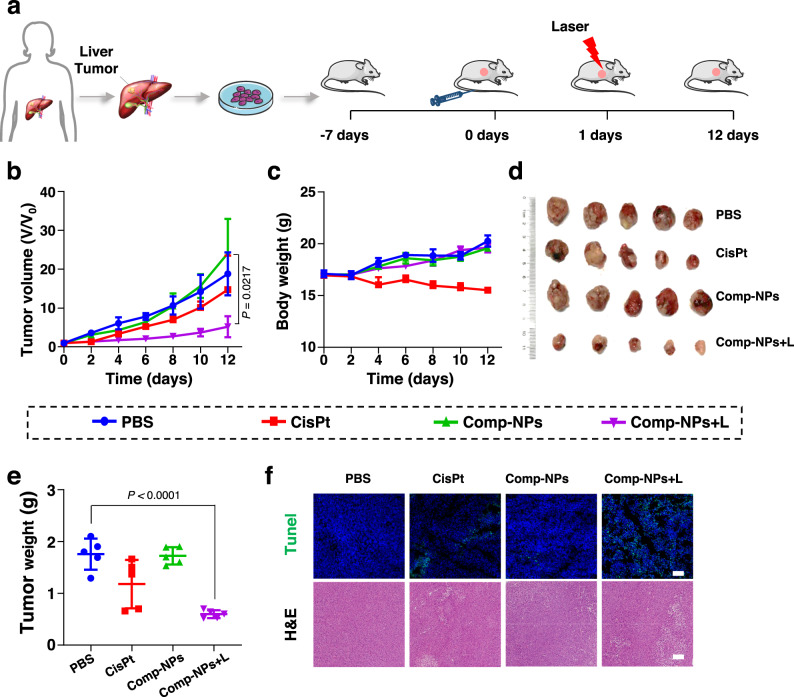
PDT properties of Comp-NPs evaluated towards a multi-drug resistant patient-derived hepatocellular carcinoma inside a mouse model. **a** Schematic illustration of the establishment of the mouse model as well as the subsequent administration and treatment schedules with Comp-NPs. Phosphate-buffered saline (PBS) and cisplatin (CisPt) were used as controls. **b** Tumor growth inhibition curves (*n* = 5 mice) upon treatments with Comp-NPs (6 mg BODIPY kg^−1^) or cisplatin (0.5 mg Pt kg^−1^) in the dark or upon irradiation (650 nm, 0.1 W cm^−2^, 60 J cm^−2^, 10 min). Error bars represent mean ± SD. Statistical analysis was performed by two-tailed unpaired *t* test. **c** Body weight of the mice (*n* = 5 mice) upon various treatments with Comp-NPs (6 mg BODIPY kg^−1^) or cisplatin (0.5 mg Pt kg^−1^) in the dark or upon irradiation (650 nm, 0.1 W cm^−2^, 60 J cm^−2^, 10 min). Error bars represent mean ± SD. **d** Photographs of the tumors after various treatments. **e** Weight of the tumors from (**d**) (*n* = 5 mice). Error bars represent mean ± SD. Statistical analysis was performed by two-tailed unpaired *t* test. **f** H&E as well as terminal deoxynucleotidyl transferase dUTP nick end labeling stain of tumor tissue slices. (The images are representative of 5 mice per group). The experiment was repeated independently 3 times with similar results. Tunel: scale bar = 50 μm. H&E: scale bar = 100 μm. Source data are provided as a Source Data file.

Hepatocellular carcinoma, also referred to as liver cancer, has one of the highest clinical incidences^63^. While there exist effective treatments in the clinics, certain parts of the liver are poorly accessible or associated with drug resistance, limiting the therapeutic options for patients. To overcome these drawbacks, there is a need for the development of therapeutic drugs and methods. To evaluate the ability of the here reported nanoparticles to treat hepatocellular carcinoma tumors, the biological properties of Comp-NPs were studied against human hepatocellular carcinoma (JHH7) cells in the dark as well as upon irradiation (650 nm, 0.1 W cm^−2^, 60 J cm^−2^, 10 min). While being non-toxic in the dark (IC_50,dark_ > 6 μg/mL BODIPY), Comp-NPs had a strong cytotoxic effect upon exposure to irradiation (IC_50,light_ = 0.45 ± 0.02 μg/mL BODIPY, Supplementary Fig 36a). The ability to induce ICD in JHH7 cells upon treatment with the nanoparticles and exposure to light was studied through monitoring for ICD specific hallmarks. The translocation of CRT to the cell surface upon treatment with Comp-NPs in the light was noticed by flow cytometry (Supplementary Fig 36b). Using immunofluorescence CLSM, the migration of HMGB1 from the nucleus into the cytoplasm was observed (Supplementary Fig 36c). The treatment with the nanoparticles triggered the release of ATP (Supplementary Fig 36d). Combined these findings indicate the induction of ICD in JHH7 cells upon treatment with Comp-NPs and exposure to irradiation. Based on these promising preliminary findings, the therapeutic properties of Comp-NPs were further studied against multi-drug resistant patient-derived hepatocellular carcinoma tumors inside a mouse model. The cancer cells were obtained from a patient with reoccurring tumors despite multiple rounds of surgery and chemotherapy. While the tumors of the animals injected with the nanoparticles in the dark grew exponentially in a similar manner as the mice injected with saline, a strong tumor reduction for the animal injected with Comp-NPs and exposed to irradiation was observed within a single drug and light treatment (Fig. 8a). Due to the drug resistance of the tumor, the treatment with cisplatin showed only a minimal effect on tumor remission. These findings are very promising and indicate the potential of Comp-NPs to treat multi-drug resistant and clinically challenging tumors (Fig. 8b). Importantly, the animals treated with Comp-NPs did not show any signs of pain or discomfort as well as did not lose body weight (Fig. 8c). Following the treatment period, their major organs analyzed. As no pathological alternations were noticed (Supplementary Fig 37), the high biocompatibility of the treatment is indicated. In addition, the tumors were collected and weighted (Fig. 8d: Photographs of the tumor, Fig. 8e: Tumor weight). While the animals injected with saline or treated with Comp-NPs in the dark showed an average tumor weight of ~1.8 g, the mice treated with Comp-NPs and exposed to irradiation showed a strongly reduced tumor weight of ~ 0.6 g, highlighting the strong tumor growth inhibition effect. For a deeper insight, the tumorous tissue was histologically analyzed. The tumor slices of the mice treated with Comp-NPs and exposed to irradiation showed cellular and tissue damage (Fig. 8f), indicative of the efficient PDT treatment. Overall, these results demonstrated the ability of Comp-NPs to cause a strong tumor reduction of a multi-drug resistant clinically challenging hepatocellular carcinoma inside the animal model, suggesting promising properties for clinical development.

### Biochemical mechanism of action in a hepatocellular carcinoma patient-derived tumor-bearing mouse model

**Fig. 9:**
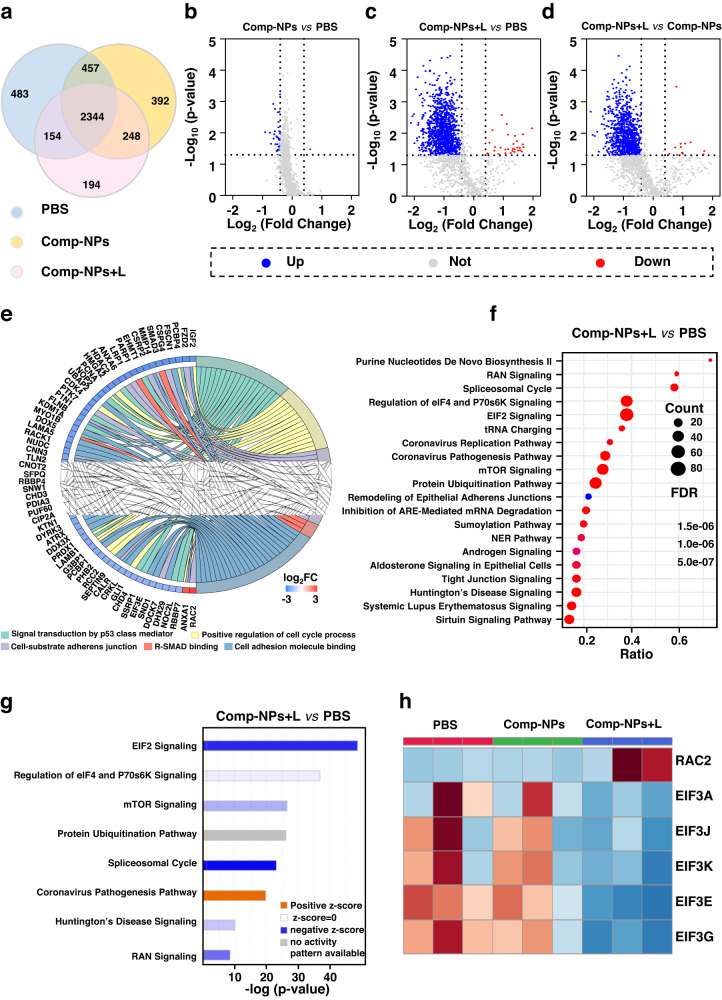
Biochemical mechanism of action of the treatment of multi-drug resistant patient-derived hepatocellular carcinoma inside a mouse model with Comp-NPs. **a** Venn diagram of the number of proteins detected upon the respective treatment (*n* = 3 mice). **b**–**d** Volcanic plot of the differences in protein expression levels inside the animal models upon the respective treatment. The up-regulated proteins are highlighted in blue, down-regulated proteins are highlighted in red, and the unchanged proteins are marked in gray (Log_2_*FC* > 0.8 and *p* < 0.05) (*n* = 3 mice). Statistical analysis was performed by two-tailed unpaired *t* test. **e** Proteins enrichment GOcircos analysis upon treatment with Comp-NPs and exposure to irradiation (*n* = 3 mice). **f** KEGG pathway enrichment analysis upon treatment with Comp-NPs and exposure to irradiation (*n* = 3 mice). Statistical analysis was performed by two-tailed unpaired *t* test. **g** Positive and negative correlation of differential pathway analysis upon treatment with Comp-NPs and exposure to irradiation (*n* = 3 mice). Statistical analysis was performed by two-tailed unpaired *t* test. **h** Expression of mTOR pathway-related proteins (*n* = 3 mice).

For a deeper insight into the mechanism of action of the nanoparticles, proteomics studies have been performed. These investigations allow for an understanding of signaling pathways, metabolism, and cellular responses to the treatment. For this purpose, hepatocellular carcinoma patient-derived tumor xenograft models were treated with phosphate-buffered saline or Comp-NPs in the dark as well as upon irradiation and the up- or down-regulation of specific proteins studies by mass spectrometry. While in total 4272 differential proteins were detected, among these 2344 proteins were found in all mice models (Fig. 9a). An analysis of the protein expression levels between the treatment with phosphate-buffered saline or Comp-NPs in the dark indicated that 33 proteins were up-regulated and 1 protein was down-regulated (Fig. 9b). Contrary, the comparison of the expression levels of the xenograft models treated with phosphate-buffered saline and Comp-NPs in the light showed that 1146 proteins were up-regulated and 31 proteins were down-regulated (Fig. 9c), highlighting the different cellular response upon exposure to light. For an understanding of the PDT treatment, the protein expression levels of the mice models treated with Comp-NPs in the dark or in the light were compared. The results demonstrated that 948 proteins were up-regulated and 12 proteins were down-regulated (Log_2_*FC* > 0.8 and *p* < 0.05, Fig. 9d).

The signaling pathways involved in the regulation of differential proteins upon treatment with Comp-NPs and exposure to light were studied using a GOcircos analysis. The results showed that the p53 mediated signal transduction, cell cycle regulation, cell-substrate adherens junction, R-SMAD binding, and cell adhesion molecule binding pathways were miss regulated (Fig. 9e). Previous studies have identified these pathways to be involved in cancer cell growth^64^, adhesion^65^, and metastasis^66^. The KEGG enrichment analysis revealed that the treatment with Comp-NPs and exposure to light strongly influenced the mTOR signaling, eIF4 and p70S6K signaling, eIF2 signaling and RNA signaling pathways (Fig. 9f). Of particular interest is the modification of the mTOR signaling pathway (Fig. 9g, Supplementary Fig 38) which is closely related to tumorigenesis^67^, metastasis development and chemotherapy resistance^68^. Further analyses revealed that the mTOR signaling pathway is influencing the expression of the EIF3 protein family and RAC2 protein (Fig. 9h). An inhibition of the mTOR signaling pathway correlates to a miss regulation of the expression of the elF4 and P70S6K proteins. Studies have shown that the EIF3 protein family is associated with the development of metastases^69^ and evolution of tumors by regulation of the cell cycle, RNA synthesis, and DNA repair^70^. RAC2 regulates cell responses, secretion processes and apoptotic cellular processes^71^. For additional verification of the involvement of the mTOR signaling pathway in the therapeutic mechanism of the nanoparticles, the expression levels of mTOR and P70S6K proteins in 4T1 cells was studied using Western Blot. The results show a strong reduction of mTOR and P70S6K proteins upon treatment with Comp-NPs and exposure to light (Supplementary Fig 39), highlighting the inhibition of the mTOR signaling pathway. As such the miss regulation of the mTOR signaling pathway could present a target for anticancer drug development. Overall, these results indicate the ability of Comp-NPs upon exposure to light to interact in various cellular processes. The influence of the mTOR signaling pathway could present beneficial for a highly efficient treatment as well as present a mechanism for therapeutic intervention.

**Fig. 10:**
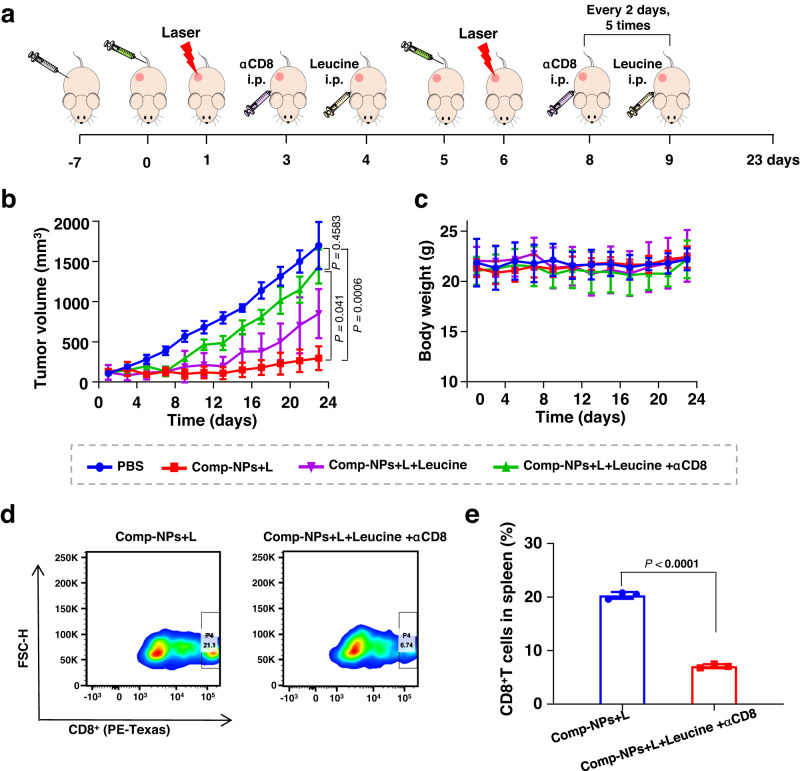
PDT properties of Comp-NPs evaluated in a mouse model bearing a 4T1 tumor. **a** Schematic illustration of the establishment of the tumor mouse model, administration and treatment schedules with Comp-NPs (6 mg BODIPY kg^−1^), Leucien, and/or αCD8 in the dark or upon irradiation (650 nm, 0.1 W cm^−2^, 60 J cm^−2^, 10 min). Phosphate-buffered saline (PBS) was used as a control. **b** Mean tumor growth inhibition curves (*n* = 6 mice) of the tumor upon treatment. Error bars represent mean ± SD. Statistical analysis was performed by one-way ANOVA with Tukey’s multiple comparisons test. **c** Body weight of the mice (*n* = 6) upon treatment. Error bars represent mean ± SD. **d**, **e** Analysis of the levels of CD8^+^ T-cells inside the spleens of the treated mouse models (*n* = 3 mice). Error bars represent mean ± SD. Statistical analysis was performed by two-tailed unpaired *t* test. Source data are provided as a Source Data file.

The proteomics results indicate that Comp-NPs could therapeutically intervene inside the mouse model upon inhibition of the mTOR signaling pathway. To verify these findings, 4T1 tumor-bearing mice models were treated with Comp-NPs, Comp-NPs and Leucine, or a combination of Comp-NPs, Leucine and αCD8 (treatment schedule: Fig. 10a). Leucine is an mTOR pathway agonist that upregulates the mTOR protein expression, thereby activating the mTOR signaling pathway and promoting tumor growth. αCD8 is a CD8^+^ T cell-specific antibody that specifically binds to CD8^+^ T cells in tumorous tissues, preventing CD8^+^ T cells from attacking tumor cells and therefore leading to immune escape of the tumor cells. Importantly, the mice behave normally with signs for pain or stress and did not lose or gain any weight, indicating the high biocompatibility of the treatment (Fig. 10c). The co-administration of Leucine and/or αCD8 with Comp-NPs demonstrated to reduce the therapeutic efficiency of the treatment (Fig. 10b). Since the co-administration with Leucine reduced the therapeutic efficacy, it is strongly suggested that Comp-NPs therapeutically intervenes through inhibition of the mTOR signaling pathway. In addition, the co-administration with αCD8 further diminished the therapeutic efficiency indicating that the part of the therapeutic effect of Comp-NPs is caused by immune activation. For further verification of reduced immunogenic effect upon co-treatment with Leucine and αCD8, the spleens of the treated animals were collected at day 23 and the levels of CD8^+^ T cells analyzed by flow cytometry. The results showed a drastic reduction of CD8^+^ T cells from 21% to 7% (Fig. 10d, e). Combined these findings suggest that Comp-NPs therapeutically interacts inside the mouse model through inhibition of the mTOR signaling pathway and activates the immune response inside the animal model.

## Discussion

In summary, the development of a theranostic multifunctional nanomaterial for applications in the diagnosis and treatment of tumors is presented. The nanoparticles were found to be highly stable under physiological conditions but quickly dissociate in the presence of ROS. The incorporation of a photosensitizer into the polymer backbone allows for the efficient treatment of breast cancer monolayer cells, multicellular tumor spheroids as well as tumor-bearing mice. The use of a chromophore irradiation at 808 nm presents the ability to detect tumorous tissue. Insights into the mechanism of action revealed that the phototoxic effect of the nanomaterial is caused by immunogenic cell death in cancer cells as well as the animal model, presenting a multimodal treatment by localized cellular damage and the inducting of an immune response of the organism. Based on this combined therapeutic effect, the nanoparticles were able to strongly reduce the volume of the primary tumor as well as eradicate tumor metastases within a single treatment. For a more realistic assessment, the therapeutic properties of the nanoparticles were investigated towards a multi-drug resistant clinically challenging hepatocellular carcinoma inside an animal model. The nanomaterial was found with a strong tumor growth inhibition effect within a single drug and light doses. Proteomics analyses revealed that the nanoparticles interact mTOR signaling pathway, which is involved in tumor evolution and reoccurrence, and in the development of metastases. We are confident that the here described approach of targeting the mTOR signaling pathway could present a promising target for anticancer agents. The ability of the nanoparticles to intervene through the localized generation of oxidative stress and the induction of an immune response presents a promising medicinal method to prevent or treat tumor metastases as well as tumor reoccurrences. The combination of diagnosis and therapy in a single nanomaterial could represent an alternative tool for theranostic drug development.

## Methods

### Ethical Approval

All samples from patients were obtained following written consent and all experiments were conducted in compliance with the ethical regulations for human samples. The experiments received approval from the People’s Liberation Army (PLA) General Hospital and the Clinical Trial Registry (ChiCTR2100047481). In our study, patient-derived material has been only used for the generation of the PDX model with informed consent from the patient. All animal experiments were conducted in compliance with the ethical regulations for animal testing and received approval from the Peking University Institutional Animal Care and Use Committee (LA2021316). The mice were euthanized in accordance with the animal ethics guidelines of animal welfare regulations when the tumor volume reached 2000 mm^3^. Mice were euthanized via CO_2_ inhalation at the end point of the study.

### Materials

Methanol, toluene, dichloromethane, ethyl acetate, tetrahydrofuran and petroleum ether were purchased from Concord (Tianjin, China). Methoxyl-poly(ethylene glycol) (mPEG_5000_) and hydrogen peroxide (H_2_O_2_) were purchased from Energy Chemical (Shanghai, China). 4,9-dibromo-6,7-*bis*(4-hexylphenyl)-[1,2,5]thiadiazolo[3,4-g]quinoxaline (DPTQ6-2Br) was purchased from Alfa (Zhengzhou, China). Cisplatin (purity 99%) was purchased from Shandong Boyuan Pharmaceutical Co., Ltd. (Shandong, China). 3-(4,5-dimethylthiazol-2-yl)-2,5-diphenyltetrazolium bromide (MTT) and sodium dodecyl sulfate (SDS) were purchased from Beyotime (Shanghai, China). Cell culture vessels were purchased from Corning (Corning, NY, USA). Roswell Park Memorial Institute -1640 (RPMI-1640) medium, fetal bovine serum (FBS), 0.25% trypsin-EDTA, and penicillin/streptomycin (P/S) were purchased from Gibco (Gran Island, NY, USA). 2-(4-Amidinophenyl)-1H-indole-6-carboxamidine (DAPI) was purchased from Sigma-Aldrich (Shanghai, China). 2’,7’-dichlorodihydrofluorescein diacetate (DCFH-DA), Annexin V-FITC/Propidium iodide (PI) apoptosis detection kit, Hoechst 33258 and Calcein-AM/PI double stain kit, ATP assay kit were purchased from Beyotime Institute of Biotechnology (Jiangsu, China). Female Balb/c mice and female Balb/c nude mice (4–6 weeks) are used in the animal experiments.

### Instrumentation and methods

^1^H- and ^13^C-NMR spectra were recorded on a 300 MHz or 400 MHz NMR spectrometer (Bruker). Chemical shifts (δ) are reported in parts per million (ppm) referenced to tetramethylsilane (δ 0.00) ppm using the residual proton solvent peaks as internal standards. Absorption spectra were measured on a spectrophotometer (TU-1901). Photofluorescence (PL) spectra were recorded on a fluorescence spectrometer (FLS980). Gel permeation chromatography (GPC) measurements were performed on a GPC-1515 instrument (USA) [Column type: Styragel HT6E^.^2, HT2, Column length: 7.8^.^ 30 mm, Detector: 2414, pump 1515, Test temperature: 40 °C, flow: 1 mL min^−1^, eluent: *N*,*N*-dimethylformamide]. Transmission electron microscopy (TEM) images were recorded on a Hitachi HT-7700 TEM. Dynamic light scattering measurements were performed on a Zetasizer (Nano ZS, UK).

### Synthesis of ethyl (*E*)-3-(4-(diphenylamino)phenyl)acrylate (2)

4-Diphenylbenzaldehyde (5.46 g, 20 mmol) and ethoxylmethyltriphenylphosphine (20 mmol) were dissolved in toluene (60 mL) and stirred at room temperature for 48 h under nitrogen atmosphere. After this time, the solvent was removed under reduced pressure. The crude product was purified by chromatography on silica gel using petroleum ether /dichloromethane (v/v = 10/1) as the eluent. The fractions containing the product were united and the solvent removed under reduced pressure. The compound was dried under high vacuum. Yield: 85%. ^1^H-NMR (400 MHz, CDCl_3_): δ = 7.61 (d, *J* = 15.9 Hz, 1H), 7.35 (d, *J* = 8.7 Hz, 2H), 7.28 – 7.24 (m, 5H),7.10 – 7.04 (m, 6H), 6.98 (dd, *J* = 9.1, 2.2 Hz, 2H), 6.27(d, *J* = 15.9 Hz,1H), 4.21(q, *J* = 7.1 Hz, 2H), 1.30 (t, *J* = 7.1 Hz, 3H) ppm. The obtained analytic data was found to be in agreement with the previous literature^72^.

### Synthesis of ethyl 3-(4-(diphenylamino)phenyl)propanoate (3)

A solution of ethyl (E)-3-(4-(diphenylamino)phenyl)acrylate (6.86 g, 20 mmol) in methanol (60 mL) was suspended with Pt/C (1 g, 5 %) under a hydrogen atmosphere for 48 h. After this time, the solution was filtered over celite and the solvent was removed under reduced pressure. The compound was dried under high vacuum. Yield: 96%. ^1^H-NMR (400 MHz, CDCl_3_), *δ* 7.24 – 7.19 (m, 4H), 7.09 – 6.95 (m, 10H),4.71 (q, *J* = 22.0 Hz, 2H) 2.93 (t, *J* = 7.8 Hz, 2H), 2.63 (t, *J* = 7.8 Hz, 2H),1.27 (t, *J* = 14.2 Hz, 3H) ppm. The obtained analytic data was found to be in agreement with the previous literature^72^.

### Synthesis of 3-(4-(diphenylamino)phenyl)propan-1-ol (4)

Ethyl 3-(4-(diphenylamino)phenyl)propanoate (6.92 g, 20 mmol) was dissolved in anhydrous tetrahydrofuran (40 mL) and the solution cooled down to 0 °C. Three portions of lithium aluminum hydride (1520 mg, 40 mmol) were slowly added over a time period of 1 h. The ice bath was removed and the mixture was stirred at room temperature for 6 h. Sodium hydroxide solution (10 %) was added dropwise to the solution until no gas formation was observed. The solution was then filtered over celite and the solvent was removed by a rotary evaporator. The crude product was purified by chromatography on silica gel using petroleum ether /dichloromethane (v/v = 10/1) as the eluent. The fractions containing the product were united and the solvent removed under reduced pressure. The compound was dried under high vacuum. Yield: 95%. ^1^H-NMR (400 MHz, CDCl_3_), *δ* 7.25 (dd, *J* = 8.3, 7.6 Hz, 4H), 7.07 - 6.95 (dd, *J* = 42.9 Hz, 9H), 3.75 (t, *J* = 21.8 Hz, 3H), 2.69 – 2.64 (m, 2H), 1.92(dt, *J* = 13.0, 19.4 Hz, 2H) ppm. The obtained analytic data was found to be in agreement with the previous literature^72^.

### Synthesis of 4-(3-((tert-butyldimethylsilyl)oxy)propyl)-*N*,*N*-diphenylaniline (5)

3-(4-(diphenylamino)phenyl)propan-1-ol (3.03 g, 10 mmol), tert-butyldimethylsilyl chloride (2.25 g, 15 mmol), and imidazole (1.17 g, 15 mmol) were dissolved dimethylformamide (40 mL) and the solution was stirred at room temperature for 3 h. After this time, deionized water (100 mL) was added and the organic phase was extracted three times with ethyl acetate (3×30 mL). The solvent was removed under reduced pressure. The crude product was purified by chromatography on silica gel using petroleum ether /ethyl acetate (v/v = 10/1) as the eluent. The fractions containing the product were united and the solvent removed under reduced pressure. The compound was dried under high vacuum. Yield: 90%. ^1^H-NMR (300 MHz, CDCl_3_), *δ* 7.23 – 7.19 (m, 4H), 7.078– 6.94 (m, 11H), 3.66 (t, *J* = 6.3 Hz,2H), 2.65 (t, *J* = 7.7 Hz, 2H), 1.87 – 1.80 (m, 2H), 0.91 (s, 9H), 0.05 (s, 6H) ppm. The obtained analytic data was found to be in agreement with the previous literature^72^.

### Synthesis of 4-bromo-*N*-(4-(3-((tert-butyldimethylsilyl)oxy)propyl)phenyl)-*N*-phenylaniline (6)

4-(3-((Tert-butyldimethylsilyl)oxy)propyl)-N,N-diphenylaniline (4.18 g, 10 mmol) was dissolved in dichloromethane (60 mL) and the solution cooled down to 0 °C. Three portions of N-bromsuccinimid (2.25 g, 15 mmol) were slowly added over a time period of 1 h. The ice bath was removed and the mixture was stirred at room temperature for 4 h. The solvent was removed under reduced pressure. The crude product was purified by chromatography on silica gel using petroleum ether /ethyl acetate (v/v = 10/1) as the eluent. The fractions containing the product were united and the solvent removed under reduced pressure. The compound was dried under high vacuum. Yield: 88%. ^1^H-NMR (300 MHz, CDCl_3_), *δ* 7.26 – 7.15 (dd, *J* = 31.5, 4H), 7.05 (ddd, *J* = 30.2, 21.2, 8.6 Hz, 9H), 3.61 (t, *J* = 6.2 Hz, 2H), 2.61 (t, *J* = 7.7 Hz, 2H), 1.82 – 1.73 (m, 2H), 0.85 (d, *J* = 9.9 Hz, 9H), 0.00 (s, 6H) ppm. The obtained analytic data were found to be in agreement with the previous literature^72^.

### Synthesis of 4-(3-((tert-butyldimethylsilyl)oxy)propyl)-*N*-phenyl-*N*-(4-(4,4,5,5-tetramethyl- 1,3,2-dioxaborolan-2-yl)phenyl)aniline (7)

Bis(triphenylphosphine)palladium(II) dichloride(129 mg, 0.175 mmol), potassium acetate (413 mg, 4.21 mmol), bis(pinacolate)diboron (535 mg, 2.11 mmol) and 4-bromo-N-(4-(3-((tert-butyldimethylsilyl)oxy)propyl)phenyl)-N-phenylaniline (129 mg, 0.175 mmol) were dissolved in dioxane (20 mL) under an argon atmosphere. The reaction mixture was heated at 80 °C for 12 h. After this time, deionized water (40 mL) was added and the organic phase was extracted three times with ethyl acetate (3×50 mL). The organic phase was dried over anhydrous magnesium sulfate and the solvent was removed under reduced pressure. The crude product was purified by chromatography on silica gel using petroleum ether /ethyl acetate (v/v = 10/1) as the eluent. The fractions containing the product were united and the solvent removed under reduced pressure. The compound was dried under high vacuum. Yield: 60%. ^1^H-NMR (400 MHz, CDCl_3_), *δ* 7.61(d, *J* = 8.3 Hz, 2H), 7.19 – 7.17 (m, 2H), 7.04 – 7.02 (d, *J* = 7.1 Hz, 4H), 6.98 – 6.94 (m, 4H), 3.61-3.58 (m, 2H), 2.59 – 2.57 (m, 2H), 1.82 – 1.75 (d, *J* = 27.1 Hz, 2H), 1.27 (s, 12H), 0.86 (s, 9H), 0.00 (s, 6H) ppm. The obtained analytic data was found to be in agreement with the previous literature^72^.

### Synthesis of 4,4’-(6,7-bis(4-hexylphenyl)-[1,2,5]thiadiazolo[3,4-g]quinoxaline-4,9-diyl)bis(N-(4-(3-((tert-butyldimethylsilyl)oxy)propyl)phenyl)-N-phenylaniline) (9)

4-(3-((tert-butyldimethylsilyl)oxy)propyl)-N-phenyl-N-(4-(4,4,5,5-tetramethyl- 1,3,2-dioxaborolan-2-yl)phenyl)aniline (407.5 mg, 0.75 mmol), 4, 9-dibromo-6, 7-bis (4-hexylphenyl)-[1,2,5] thiadiazole and [3,4-g] quinoxaline (compound 8, 150.0 mg, 0.25 mmol), and tetrakis-(triphenylphosphin)-palladium (462.2 mg, 0.40 mmol) were dissolved in toluene (20 mL) under an argon atmosphere. An aqueous solution of potassium carbonate (1 M, 3 mL) was added and the reaction mixture was heated at 95 °C for 12 h. After this time, the solution was cooled down and diluted with water (40 mL). The organic phase was extracted with ethyl acetate (3×50 mL). The combined organic layers were washed with water (2×20 mL), dried over anhydrous magnesium sulfate, and the solvent removed under reduced pressure. The product was isolated by column chromatography on silica gel using petroleum ether:ethyl acetate (v/v = 20/1). The fractions containing the product were united and the solvent was removed under reduced pressure. The compound was dried under high vacuum. Yield: 50%. ^1^H-NMR (300 MHz, CDCl_3_): δ 7.98-7.95 (d, *J* = 9.0 Hz, 4H), 7.58-7.55 (d, *J* = 8.2 Hz, 4H),7.29-7.24 (d, *J* = 14.5, 8H), 7.22-7.07 (m, 18H), 3.71-3.66 (t, *J* = 12.8,4H), 2.69-2.60 (m, 8H), 1.88-1.81 (m, 4H), 1.65- 1.58 (m, 4H), 1.31 (s, 12H), 0.92 (s, 18H), 0.89 (s, 6 H), 0.09 (s, 12H) ppm; ^13^C-NMR (100 MHz, CDCl_3_): δ 162.8, 147.0, 143.7, 128.4, 127.2, 124.5, 123.7, 121.9, 120.2, 61.4, 30.7, 28.7, 27.8, 25.0, 21.5, 17.3, 13.1, -6.3 ppm; MALDI-TOF-MS: [M + H]^+^ calcd. for C_86_H_103_N_6_O_2_SSi_2_^+^: 1339.7, found: 1339.2.; HR-ESI-MS: calcd. for [M + H]^+^ C_86_H_103_N_6_O_2_SSi_2_^+^: 1339.7402, found: 1339.7403.

### Synthesis of 4,4’-(6,7-bis(4-hexylphenyl)-[1,2,5]thiadiazolo[3,4-g]quinoxaline-4,9-diyl)bis(N,N-diphenylaniline) (M1)

4,4’-(6,7-Bis(4-hexylphenyl)-[1,2,5]thiadiazolo[3,4-g]quinoxaline-4,9-diyl)bis(N-(4-(3-((tert-butyldimethylsilyl)oxy)propyl)phenyl)-N-phenylaniline) (30 mg, 0.27 mmol) and Amberlyst 15 (100 mg) were suspended in dichloromethane (15 mL) and the mixture was stirred at room temperature for 3 h. After this time, the solid was removed by filtration. The solvent of the filtrate was removed under reduced pressure. The product was isolated by column chromatography on silica gel using dichloromethane: methanol (v/v = 20/1). The fractions containing the product were united and the solvent was removed under reduced pressure. The compound was dried under high vacuum. Yield: 40%. ^1^H-NMR (300 MHz, CDCl_3_): δ 7.98-7.96 (d, *J* = 8.4 Hz, 4H), 7.60-7.57 (d, *J* = 8.0 Hz, 4H), 7.30 (s, 8H), 7.18-7.12 (t, *J* = 18.3 Hz, 16H), 3.76-3.72 (t, *J* = 12.5 Hz, 4H), 2.66-2.61 (t, *J* = 15.5 Hz, 4H), 1.99-1.90 (m, 4H), 1.63- 1.61 (d, *J* = 14.4 Hz,4H), 1.31-1.26 (d, *J* = 18.8 Hz, 16H), 0.89-0.87 (t, *J* = 14.0 Hz, 6H); ^13^C-NMR (75 MHz, CDCl_3_):153.0, 143.4, 134.2, 134.1, 130.0, 128.3, 104.3, 62.4, 34.3, 31.5, 29.7, 29.0, 22.7, 14.2; MALDI-TOF-MS: [M + H]^+^ calcd. for C_74_H_75_N_6_O_2_S^+^:1112.5, found: 1112.2. HR-ESI-MS: calcd. for [M + H]^+^ C_74_H_75_N_6_O_2_S^+^: 1111.5672, found: 1111.5667.

### Synthesis of 5,5-difluoro-1,3,7,9-tetramethyl-10-phenyl-5*H*–4*λ*^4^,5*λ*^4^-dipyrrolo[1,2-*c*:2’,1’-*f*][1,3,2]diazaborinine (12)

Benzaldehyde (0.32 g, 3.0 mmol), 2,4-dimethylpyrrole (0.63 g, 6.6 mmol), and several drops of trifluoroacetic acid were dissolved in tetrahydrofuran (90 mL). The mixture was stirred at room temperature overnight. After this time, a solution of 2,3-dichloro-5,6-dicyano-p-benzoquinone (0.68 g, 3.0 mmol) in tetrahydrofuran (120 mL) was added and the mixture was stirred for another 4 h. After this time, a solution of bortrifluoriddiethyletherat (18 mL, 0.15 mol) in diisopropylethylamine (22 mL, 0.13 mol) was added dropwise to the mixture. The mixture was stirred at room temperature overnight. After this time, undissolved solids were removed upon filtration over celite. The solvent was removed under reduced pressure. The residue was redissolved in dichloromethane (100 mL) and the solution was washed with 5% aqueous sodium bicarbonate solution (100 mL) and water (2 × 100 mL). The organic phase was dried over anhydrous magnesium sulfate. The crude product was purified by chromatography on silica gel using dichloromethane as the eluent. The fractions containing the product were united and the solvent removed under reduced pressure. The compound was dried under high vacuum. Yield: 43%. ^1^H-NMR (300 MHz, DMSO-*d*_*6*_): δ 7.57 (s, 3H), 7.38 (s, 2H), 6.17 (s, 2H), 2.45 (s, 6H), 1.34 (s, 6H). The obtained analytic data was found to be in agreement with the previous literature^73^.

### Synthesis of 5,5-difluoro-2,8-diiodo-1,3,7,9-tetramethyl-10-phenyl-5*H*-4*λ*^4^,5*λ*^4^-dipyrrolo[1,2-*c*:2’,1’-*f*][1,3,2]diazaborinine (13)

5,5-Difluoro-1,3,7,9-tetramethyl-10-phenyl-5*H*--4*λ*^4^,5*λ*^4^-dipyrrolo[1,2-*c*:2’,1’*f*][1,3,2]diazaborinine (0.32 g, 1.0 mmol) and iodine (0.63 g, 2.5 mmol) were dissolved in ethanol (25 mL). A solution of iodic acid (0.35 g, 2 mmol) in water (5 mL) was added dropwise. The mixture was heated at 60 °C for 2 h. After this time, undissolved solids were removed upon filtration over celite. The solvent was removed under reduced pressure. The crude product was purified by chromatography on silica gel using dichloromethane/ethyl acetate (v/v = 2/1) as the eluent. The fractions containing the product were united and the solvent removed under reduced pressure. The compound was dried under high vacuum. Yield: 88%. ^1^H-NMR (300 MHz, DMSO-*d*_*6*_): δ 7.62 (s, 3H), 7.42 (s, 2H), 2.56 (s, 6H), 1.34 (s, 6H). The obtained analytic data was found to be in agreement with the previous literature^54^.

### Synthesis of 2,2’-((((1E,1’E)-(5,5-difluoro-2,8-diiodo-1,9-dimethyl-10-phenyl-5*H*-4*λ*^4^,5*λ*^4^-dipyrrolo[1,2-*c*:2’,1’-*f*][1,3,2]diazaborinine-3,7-diyl)bis(ethene-2,1-diyl))bis(4,1-phenylene))bis(oxy))bis(ethan-1-ol) (M2)

5,5-Difluoro-2,8-diiodo-1,3,7,9-tetramethyl-10-phenyl-5*H*-4*λ*^4^,5*λ*^4^-dipyrrolo[1,2-*c*:2’,1’-*f*][1,3,2]diazaborinine (**13**) (0.17 g, 0.3 mmol), glacial acetic acid (0.6 mL, 10.5 mmol), 4-(2-hydroxyethoxy)benzaldehyde (0.1 g, 0.6 mmol), and piperidine (0.8 mL, 8.0 mmol) were dissolved in toluene (40.0 mL). The mixture was heated at reflux for 2 h. During the reaction the generated water was azeotropically removed using a Dean-Stark apparatus. After this time, the solvent was removed under reduced pressure. The crude product was purified by chromatography on silica gel using chloroform/methanol (v/v = 50/1) as the eluent. The fractions containing the product were united and the solvent removed under reduced pressure. The compound was dried under high vacuum. Yield: 30%. ^1^H-NMR (300 MHz, DMSO-*d*_*6*_): δ 8.11-8.05(d, 2H), 7.61-7.43(m, 7H), 7.46-7.41(d, 4H), 7.08-7.06(d, 4H), 4.93-4.89(t, 2H), 4.06-4.03(t, 4H), 3.77-3.72(q, 4H), 1.41(s, 6H). The obtained analytic data was found to be in agreement with the previous literature^54^.

### Synthesis of P1

M1 (10 mg, 0.009 mmol), L-lysine diisocyanate (33.52 mg, 0.171 mmol), and 2,2´-(propane-2,2-diylbis(sulfanediyl))bis(ethan-1-ol) (40.7 mg, 0.18 mmol) were suspended in anhydrous *N*,*N*-dimethylformamide (5 mL) for 24 h. After this time, mPEG_5000_ (100 mg, 0.02 mmol) was added to the reaction mixture. After this time, the reaction mixture was dialyzed against *N*,*N*-dimethylformamide for 24 h (MWCO: 7000 Da) to remove the unreacted monomers and oligomers. The obtained solution was freeze-dried under reduced pressure to obtained polymer as a powder P1.

### Synthesis of P2

M2 (7.85 mg, 0.009 mmol), L-lysine diisocyanate (33.52 mg, 0.171 mmol), and 2,2´-(propane-2,2-diylbis(sulfanediyl))bis(ethan-1-ol) (40.7 mg, 0.18 mmol) were suspended in anhydrous *N*,*N*-dimethylformamide (5 mL) for 24 h. After this time, mPEG_5000_ (100 mg, 0.02 mmol) was added to the reaction mixture. After this time, the reaction mixture was dialyzed against *N*,*N*-dimethylformamide for 24 h (MWCO: 7000 Da) to remove the unreacted monomers and oligomers. The obtained solution was freeze-dried under reduced pressure to obtained polymer as a powder P2.

### Preparation of NP1

P1 (10.0 mg) was dissolved in *N*,*N*-dimethylformamide (2 mL) and the solution was added dropwise into deionized water (5 mL) under continuous stirring. After 10 min, the reaction mixture was dialyzed against water for 48 h (MWCO: 7000 Da) to remove the organic solvent. The nanoparticle solution was concentrated by centrifugation at 3000 rpm for 15 min and afterwards filtrated through a 0.22 μm PES syringe driven filter (Sartorius). The nanoparticle solution was stored at 4 °C.

### Preparation of NP2

P2 (10.0 mg) was dissolved in *N*, *N*-dimethylformamide (2 mL) and the solution was added dropwise into deionized water (5 mL) under continuous stirring. After 10 min, the reaction mixture was dialyzed against water for 48 h (MWCO: 7000 Da) to remove the organic solvent. The nanoparticle solution was concentrated by centrifugation at 3000 rpm for 15 min and afterwards filtrated through a 0.22 μm PES syringe driven filter (Sartorius). The nanoparticle solution was stored at 4 °C.

### Preparation of Comp-NPs

NP1 and NP2 were mixed in a 1:1 (P1 and P2 polymer mass concentrations) to form the nanoparticle formulation Comp-NPs.

### Singlet oxygen generation measured by absorption spectroscopy

Comp-NPs (2 μg/mL BODIPY) and 1,3-diphenylisobenzofuran (0.02 mM) were dissolved in water. The solutions were aerated and irradiated (650 nm, 0.1 W cm^−2^) using different time intervals. The absorbance of the samples at 410 nm was measured during these time intervals with a TU-1901 absorption spectrometer.

### Identity of ROS species

Comp-NPs (2 μg/mL BODIPY) was incubated in phosphate-buffered saline containing 20 mM 2,2,6,6–tetramethylpiperidine or 20 mM 5,5-dimethyl-1-pyrroline *N*-oxide. Capillary tubes were filled with the solution and sintered by fire. The samples were kept in the dark or exposed to irradiation with a 650 nm laser (0.1 W cm^–2^) for 10 min. The electron spin resonance spectrum was recorded with an EPR spectrometer (A3000, BRUKER).

### Fluorescence quantum yield of Comp-NPs

The fluorescence quantum yield of Comp-NPs was determined using the fluorophore IR dye 26 as a reference. The optical absorbance was measured for both a Comp-NPs suspension and an IR dye 26 solution at 808 nm. Then their fluorescence emission intensities were measured under the same 808 nm excitation. Photofluorescence (PL) spectra were recorded on a fluorescence spectrometer (FLS980) in the 900–1700 nm region. Using the measured optical density (*OD*) and spectrally integrated fluorescence intensity (*F*), one can calculate the quantum yield of Comp-NPs according to the following formula:

$${\Phi }_{x}\left({{{{{\rm{\lambda }}}}}}\right)=	{\Phi }_{{{{{{{\rm{st}}}}}}}}\left(\lambda \right)\cdot \frac{{F}_{x}}{{F}_{{{{{{{\rm{st}}}}}}}}}\cdot \frac{{A}_{{{{{{{\rm{st}}}}}}}}\left(\lambda \right)}{{A}_{x}\left(\lambda \right)}={\Phi }_{{st}}\left(\lambda \right)\cdot \frac{{F}_{x}}{{F}_{{st}}}\cdot \frac{1-{10}^{{-{{{{{{\rm{OD}}}}}}}}_{{st}}\left(\lambda \right)}}{1-{10}^{{-{{{{{{\rm{OD}}}}}}}}_{x}\left(\lambda \right)}} \\=	0.5\%\cdot \frac{678713.5\,{{{{{{\rm{cps}}}}}}}}{5690769.5\,{{{{{{\rm{cps}}}}}}}}\cdot \frac{1-{10}^{-2.5355}}{1-{10}^{-0.1477}}=2.0\%$$

The fluorescence quantum yield of Comp-NPs was determined to be 2.0% upon excitation at 808 nm.

### H_2_O_2_ sensitivity of the polymers measured by GPC

P1 and P2 (7 mg/mL) were dissolved in *N*,*N*-dimethylformamide. Hydrogen peroxide (10 mM) was added to the solution and the polymers were analyzed by gel permeation chromatography.

### Stability of the nanoparticles observed by phosphorescence spectroscopy

The dye Nile Red (20 μg/mL) was encapsuled with Comp-NPs (2 μg/mL BODIPY) and the nanoparticles dissolved in water. The solution was exposed upon irradiation (650 nm, 0.1 W cm^−2^) and the phosphorescence spectra (600–750 nm) time-dependently (0–60 min) monitored with a SpectraMax microplate reader.

### H_2_O_2_ sensitivity of the nanoparticles measured by GPC

NP1 (10 mg/mL P1), NP2 (10 mg/mL P2) and Comp-NPs (5 mg/mL P1 and 5 mg/mL P2) were dissolved in water. H_2_O_2_ (10 mM) was added to the solution and the nanoparticles were analyzed by TEM.

### Cell culture

The mouse breast cancer (4T1), human ovarian cancer (SKOV3), cisplatin sensitive ovarian cancer (A2780) and cisplatin-resistant ovarian cancer (A2780DDP) cells were purchased from the American Type Culture Collection (Manassas, VA, USA). The 4T1, A2780, A2780DDP cells were cultivated in RPMI-1640 media. The SKOV3 cells were cultivated in DMEM media. The JHH7 cells were cultivated in DMEM media. The cell lines were complemented with 10% of fetal calf serum and a100 U/mL penicillin-streptomycin mixture. The cells were cultivated in a humidified atmosphere at 37 °C with 5% of CO_2_ and 21% O_2_. All cell lines were verified to be free of mycoplasma.

### Cellular uptake measured by confocal laser scanning microscopy

4T1 cells were incubated with the nanoparticles (1 μg/mL BODIPY) for varying time points (1, 4, 7 h) at 37 °C. The cells were incubated with the fluorescent probe Alexa Fluor® 488 phalloidin (Thermo Fisher Scientific) as a stain for cell actin cytoskeleton for 30 min at 37 °C in the dark. The cells were washed three times with PBS. Confocal images were taken with a CLSM (LSM-800, ZEISS, Germany). Comp-NPs: *λ*_ex_ = 650 nm, *λ*_em_ = 745 nm, Alexa Fluor 488: *λ*_ex_ = 495 nm, *λ*_em_ = 519 nm.

### Cellular uptake measured by flow cytometry

4T1 cells were incubated with the nanoparticles (1 μg/mL BODIPY) for varying time points (1, 4, 7 h) at 37 °C. The cells were washed three times with PBS. The cellular uptake was measured with a flow cytometry (Cytomics FC500, Beckman). Comp-NPs: *λ*_ex_ = 650 nm, *λ*_em_ = 745 nm, Alexa Fluor 488: *λ*_ex_ = 495 nm, *λ*_em_ = 519 nm.

### Generation of multicellular tumor spheroids

A suspension of 0.75% agarose in PBS was heated inside a high-pressure autoclave. The hot emulsion was transferred into a 96 well plate (50 μL per well). The plates were exposed for 3 h to UV irradiation and allowed to cool down. After this time, a cell suspension of 3^.^10^3^ cells was seeded on top of the agarose ground layer. Within two-three days MCTS were formed from the cell suspension. The multicellular tumor spheroids were cultivated and maintained at 37 °C in a cell culture incubator at 37 °C with 5% CO_2_ atmosphere. The culture medium with serum was replaced every two days. The formation, integrity, and diameter of the multicellular tumor spheroids was monitored with a CLSM.

### Penetration of multicellular tumor spheroids measured by z-stack confocal laser scanning microscopy

MCTS with a diameter of ~700 μm were treated with Comp-NPs (1 μg/mL BODIPY) by replacing 50% of the media with drug supplemented media in the dark. The MCTS was incubated with the nanoparticles for 7 h at 37 °C in the dark. After this time, the MCTS was washed three times with media. Z-stack CLSM images were taken with a microscopy (LSM-800, ZEISS, Germany). Comp-NPs: *λ*_ex_ = 650 nm, *λ*_em_ = 745 nm.

### Light induced cellular singlet oxygen generation in multicellular tumor spheroids by confocal laser scanning microscopy

MCTS with a diameter of ~700 μm were treated with Comp-NPs (1 μg/mL BODIPY) by replacing 50% of the media with drug supplemented media in the dark. The MCTS was incubated with the nanoparticles for 7 h at 37 °C in the dark. The MCTS was further incubated with the ROS specific probe 2´,7´-dichlorofluorescein diacetate for 30 min. After this time, the MCTS was washed three times with media. CLSM images were taken with a microscopy (LSM-800, ZEISS, Germany). Comp-NPs: *λ*_ex_ = 650 nm, *λ*_em_ = 745 nm, 2´,7´-dichlorofluorescein diacetate: *λ*_ex_ = 488 nm, *λ*_em_ = 520 nm.

### Light induced cellular singlet oxygen generation in 4T1 cells by flow cytometry

4T1 cells were treated with Comp-NPs (0.5 μg/mL BODIPY) by replacing 50% of the media with drug supplemented media in the dark. The cells was incubated with the nanoparticles for 7 h at 37 °C in the dark. The cells were further incubated with the ROS specific probe 2´,7´-dichlorofluorescein diacetate for 30 min. After this time, the cells were washed three times with media. The ROS production was measured with a flow cytometry (Cytomics FC500, Beckman). Comp-NPs: *λ*_ex_ = 650 nm, *λ*_em_ = 745 nm, 2´,7´-dichlorofluorescein diacetate: *λ*_ex_ = 488 nm, *λ*_em_ = 520 nm.

### Cytotoxicity

8 × 10^3^ cells were seeded on 96 well plates and allowed to adhere overnight. The cells were treated with increasing concentrations of the sample diluted in cell media achieving a total volume of 200 μL. The cells were incubated with increasing concentrations of NP1, NP2, Comp-NPs or cisplatin for 24 h and after this time the medium with serum refreshed. To study the phototoxic effect, the cells were exposed to irradiation (650 nm, 0.1 W cm^−2^, 60 J cm^−2^, 10 min). To study the dark cytotoxicity effect, the cells were not irradiated and after the incubation time the medium with serum exchanged. The cells were grown for an additional 48 h at 37 °C. After this time, the medium with serum was replaced with fresh medium containing (3-(4,5-dimethylthiazol-2-yl)-2,5-diphenyltetrazolium bromide) (MTT, 10 µL of a 5 mg/mL solution in PBS buffer) and the cells further incubated for 4 h. Acidified SDS solution was then added (100 µL/well) and the plates kept in the dark for an additional 12 h. Absorption measurements were performed on a Bio-Rad plate reader at 570 nm (peak absorbance) and at 650 nm (background absorbance).

### Apoptotic cell death

4T1 cells were incubated with the nanoparticles NP1, NP2, or Comp-NPs (0.5 μg/mL BODIPY) for 12 h at 37 °C and after this time the medium with serum refreshed. To study the phototoxic effect, the cells were exposed to irradiation (650 nm, 0.1 W cm^−2^, 60 J cm^−2^, 10 min). To study the dark cytotoxicity effect, the cells were not irradiated and after the incubation time the medium with serum exchanged. The cells were grown for an additional 12 h at 37 °C. After this time, FITC-Annexin V/Propidium Iodide as apoptosis markers were incubated for 15 min. The cell death was assessed with a flow cytometer.

### Detection of ICD hallmarks – translocation of CRT by confocal laser scanning microscopy

The cells were seeded on 24-well plates (Thermo Scientific, USA) at a density of 1 × 10^4^ cells per well. After 24 h, the medium with serum was removed and the cells were incubated with the nanoparticles (0.5 μg/mL BODIPY) diluted in cell media for 6 h. The cells were washed with phosphate-buffered saline and the cells further incubated with anti-CRT antibody (Abcam, ab92516, 1:50) for 2 h at 4 °C. Subsequently, the cells were incubated with Alexa Fluor 488-conjugated secondary antibody (Abcam, ab150077, 1:500) for 1 h. The cells were exposed to irradiation (650 nm, 0.1 W cm^−2^, 2 min). The translocation of CRT inside the cells was assessed using CLSM (LSM-800, ZEISS, Germany). Nanoparticles: *λ*_ex_ = 638 nm, *λ*_em_ = 747 nm, DAPI: *λ*_ex_ = 410 nm, *λ*_em_ = 506 nm, Alexa Fluor 488-conjugated CRT specific antibody: *λ*_ex_ = 488 nm, *λ*_em_ = 525 nm.

### Detection of ICD hallmarks—translocation of CRT by flow cytometry

The cells were seeded on 24-well plates (Thermo Scientific, USA) at a density of 1 × 10^4^ cells per well. After 24 h, the medium with serum was removed and the cells were incubated with the nanoparticles (0.5 μg/mL BODIPY) diluted in cell media for 6 h. The cells were washed with phosphate-buffered saline and the cells further incubated with anti-CRT antibody (Abcam, ab92516, 1:50) for 2 h at 4 °C. Subsequently, the cells were incubated with Alexa Fluor 488-conjugated secondary antibody (Abcam, ab150077, 1:500) for 1 h. The cells were exposed to irradiation (650 nm, 0.1 W cm^−2^, 2 min). The translocation of CRT inside the cells was assessed by flow cytometry. Nanoparticles: *λ*_ex_ = 638 nm, *λ*_em_ = 747 nm, DAPI: *λ*_ex_ = 410 nm, *λ*_em_ = 506 nm, Alexa Fluor 488-conjugated CRT specific antibody: *λ*_ex_ = 488 nm, *λ*_em_ = 525 nm.

### Detection of ICD hallmarks – secretion of adenosine triphosphate

The cells were seeded on 24-well plates (Thermo Scientific, USA) at a density of 1 × 10^4^ cells per well. After 24 h, the medium with serum was removed and the cells were incubated with the nanoparticles (0.5 μg/mL BODIPY) diluted in cell media for 6 h. The cells were washed with phosphate-buffered saline. The cells were exposed to irradiation (650 nm, 0.1 W cm^−2^, 2 min). The release of adenosine triphosphate in the cell supernatant was detected by an adenosine triphosphate assay kit following the manufacturer’s protocols.

### Detection of ICD hallmarks—secretion of nuclear HMGB1 protein

The cells were seeded on 24-well plates (Thermo Scientific, USA) at a density of 1 × 10^4^ cells per well. After 24 h, the medium with serum was removed and the cells were incubated with the nanoparticles (0.5 μg/mL BODIPY) diluted in cell media for 6 h. The cells were washed with phosphate-buffered saline and the cells further incubated with anti- nuclear HMGB1protein antibody (Abcam, ab18256, 1:50) for 2 h at 4 °C. Subsequently, the cells were incubated with Alexa Fluor 488-conjugated secondary antibody (Abcam, ab150077, 1:500) for 1 h. The cells were exposed to irradiation (650 nm, 0.1 W cm^−2^, 2 min). The secretion of nuclear HMGB1protein inside the cells was assessed using CLSM (LSM-800, ZEISS, Germany). DAPI: *λ*_ex_ = 410 nm, *λ*_em_ = 506 nm, nuclear HMGB1protein specific antibody: *λ*_ex_ = 488 nm, *λ*_em_ = 525 nm.

### Cell migration wound healing assay

4T1 cells were seeded into 35 mm dishes at a density of 6 × 10^5^ cells per well. After 24 h, the cells were incubated with the nanoparticles (0.5 μg/mL BODIPY) diluted in cell media with serum for 6 h. To study the phototoxic effect, the cells were exposed to irradiation (650 nm, 0.1 W cm^−2^, 60 J cm^−2^, 10 min). After 12 h, the medium was removed, the cells were washed twice with phosphate-buffered saline, and the cells were seeded on a new cell culture plate. Upon reaching confluence a linear wound was generated in the middle of the plate with a 200 μL micropipette tip. After 12 h, light microscopy images were recorded.

### Western blot assay

4T1 cells were incubated with the nanoparticles NP1, NP2, or Comp-NPs (0.5 μg/mL BODIPY) for 12 h at 37 °C and after this time the medium with serum refreshed. To study the phototoxic effect, the cells were exposed to irradiation (650 nm, 0.1 W cm^−2^, 60 J cm^−2^, 10 min). To study the dark cytotoxicity effect, the cells were not irradiated and after the incubation time the medium with serum exchanged. The cells were grown for an additional 12 h at 37 °C. After this time, The cells were then lysed in RIPA lysis buffer (50 mM Tris-HCl pH 7.4, 150 nM NaCl, 1%NP-40, 0.1% SDS) including 1 mM of phenylmethanesulfonyl fluoride (PMSF). The protein lysate was obtained by centrifugation at 12000 g for 15 min at 4 °C. Then, the lysates were denatured at 100 °C and resolved by SDS-polyacrylamide gel electrophoresis (SDS-PAGE). The gels were blotted onto PVDF membrane (Merck Millipore) and blocked by 5% BSA buffer for 2 h at room temperature. After incubated with the indicated primary antibodies (anti-caspase-3 antibody, ab 184787, 1:1000; anti-β-tubulin antibody, ab78078, 1:1000, anti-β-actin antibody, ab8226, 1:1000; anti-P70 S6 Kinase (phosphor T389) antibody, ab2571, 1:1000; anti-mTOR antibody, ab32028, 1:1000) at 4 °C overnight, the membrane was washed several times with TBST buffer, incubated with corresponding secondary antibody (Peroxidase-Conjugated Goat Anti-Rabbit IgG (H + L), CAT:33101ES60, 1:5000 and Peroxidase-Conjugated Goat Anti-Mouse IgG (H + L), CAT:33201ES60, 1:5000) in 5% BSA solution for 1 h at room temperature, washed several times with TBST buffer, and then imaged withBio-Rad Chemi Doc XRS imaging system.

### Establishment of 4T1 breast mouse model

1 × 10^6^ 4T1 cells were dispersed in 150 µL of PBS and subcutaneously implanted into BALB/c female mice (6 weeks) to establish a tumor-bearing model. After a week, the tumor volumes of the mice reached approximately 100 mm^3^.

### Biodistribution in living 4T1 breast mouse model determined by phosphorescence imaging

The female Balb/c mice mice were intravenously injected in the tail vein with Comp-NPs (6 mg BODIPY kg^−1^). The phosphorescence was recorded on an In Vivo Imaging System (IVIS, Perkin Elmer). after 0.1 h, 1 h, 12 h, 24 h, 48 h. *λ*_ex_ = 650 nm, *λ*_em_ = 735 nm; *λ*_ex_ = 808 nm, *λ*_em_ = 950 nm.

### Biodistribution in scarified 4T1 breast mouse model determined by phosphorescence imaging

The female Balb/c nude mice were intravenously injected in the tail vein with Comp-NPs (6 mg BODIPY kg^−1^). 48 h after injection the animal model were scarified and the biodistribution determined by recording of the phosphorescence of the respective tissues with an IVIS spectrum imaging system (Spectrum CT, PerkinElmer). *λ*_ex_ = 650 nm, *λ*_em_ = 735 nm.

### Tumor growth inhibition in 4T1 breast mouse model

Twenty tumor-bearing female Balb/c mice (6 weeks) were randomly separated into 4 groups, resulting in five mice for each group. The mice were intravenously injected in the tail vein with Comp-NPs (6 mg BODIPY kg^−1^). After 24 h, the mice were anaesthetized and fixed in a warm three-axes holder. Before irradiation, the tumor site was disinfected with ethanol. The tumor was exposed to irradiation (650 nm, 0.1 W cm^−2^, 60 J cm^−2^, 10 min). Following irradiation, the operative area was disinfection by iodophor. The tumor volume and body weight were measured and recorded every two days. Tumor volume was calculated by the following formula: Volume = (Length * Width^2^)/2.

### Quantification of biochemical markers upon treatment of 4T1 breast mouse model

Following the previously described treatment, the levels of the biochemical markers ALT, AST, BUN and CR inside the animal models were analyzed.

### Histological examination of 4T1 breast mouse model

The major organs and tumors were collected and a slice of each one was fixated with 4% paraformaldehyde. The obtained slices were stained with hematoxylin and eosin. The histological images were taken using a CLSM (LSM-800, ZEISS, Germany).

### Detection of ICD hallmarks in tumor slices—translocation of calreticulin (CRT)

Following the previously described treatment, tumor slices from the respective animals were collected. The tissue was incubated with anti-CRT antibody (Abcam, ab92516, 1:50) for 2 h. Subsequently, the tissue was incubated with Alexa Fluor 594-conjugated secondary antibody (Abcam, ab150080, 1:500) for 1 h. The translocation of CRT inside the cells was assessed using CLSM (LSM-800, ZEISS, Germany). DAPI: *λ*_ex_ = 410 nm, *λ*_em_ = 506 nm, Alexa Fluor 488-conjugated CRT specific antibody: *λ*_*ex*_ = 590 nm, *λ*_em_ = 617 nm.

### Detection of ICD hallmarks in tumor slices – secretion of nuclear high-mobility group box 1 (HMGB1) protein

Following the previously described treatment, tumor slices from the respective animals were collected. The tissue was incubated with anti-nuclear HMGB1 protein antibody (Abcam, ab18256, 1:50) for 2 h. Subsequently, the tissue was incubated with Alexa Fluor 594-conjugated secondary antibody (Abcam, ab150080, 1:500) for 1 h. The secretion of nuclear HMGB1 protein inside the cells was assessed using CLSM (LSM-800, ZEISS, Germany). DAPI: *λ*_ex_ = 410 nm, *λ*_em_ = 506 nm, nuclear HMGB1 protein specific antibody: *λ*_ex_ = 590 nm, *λ*_em_ = 617 nm.

### Detection of ICD hallmarks in tumor slices—detection of CD8^+^ T-cells

Following the previously described treatment, tumor slices from the respective animals were collected. The tissue was incubated with CD8^+^ T cells specific fluorescent probe. The presence of CD8^+^ T cells were assessed using CLSM (LSM-800, ZEISS, Germany). DAPI: *λ*_ex_ = 410 nm, *λ*_em_ = 506 nm, CD8^+^ T-cells fluorescent probe: *λ*_ex_ = 488 nm, *λ*_em_ = 525 nm.

### Detection CD and DC cells in tumor slices

Fresh tumors and draining lymph node tissue were collected for antitumor immune response analysis via FACS. Briefly, samples were dissociated into single-cell suspensions, and then red blood cells were removed with red blood cell lysing buffer (Beyotime). After that, samples were blocked with 0.1% BSA in PBS followed by incubation with relevant antibodies for 1 h at room temperature. For characterizing T cells and DC cells in tumor, cells were stained by anti-CD3-PE (elabscience, E-AB-F1013D, 1:100), anti-CD4-PC5.5 (elabscience, E-AB-F1097J, 1:100), anti-CD8-FITC (elabscience, E-AB-F1104C, 1:100), anti-mouse IFN-γ (biolegend, 505810.1:00), anti-CD11c-PE (elabscience, E-AB-F0991D, 1:100), anti-CD80-FITC (elabscience, E-AB-F0992C, 1:100) and anti-CD86-APC (elabscience, E-AB-F0994C, 1:100). For analyzing DCs in tumor and lymph nodes, cells were stained by anti-CD11c-PE, anti-CD80-FITC, and anti-CD86-APC. All the antibodies used above were all purchased from elabscience. Flow cytometric data acquisition was performed with CytExpert software, and the data were processed using FlowJo software. Data were expressed as mean ± SD (*n* = 3).

### Establishment of a patient-derived mouse xenograft model

For patient-derived xenograft models, fresh carcinoma blocks about 2 mm^3^ were subcutaneously transplanted into the right flank of the BALB/c nude mice. The mice were used for experiments when the tumor volume reached approximately 100 mm^3^.

### Tumor growth inhibition in patient-derived mouse xenograft model

20 nude tumor-bearing nude mice were randomly separated into 4 groups, resulting in five mice for each group. The mice were intravenously injected in the tail vein with Comp-NPs (6 mg BODIPY kg^−1^). After 24 h, the mice were anaesthetized and fixed in a warm three-axes holder. Before irradiation, the tumor site was disinfected with ethanol. The tumor was exposed to irradiation (650 nm, 0.1 W cm^−2^, 60 J cm^−2^, 10 min). Following irradiation, the operative area was disinfection by iodophor. The tumor volume and body weight were measured and recorded every two days. Tumor volume was calculated by the following formula: Volume = (Length * Width^2^)/2.

### Histological examination of patient-derived mouse xenograft model

The major organs and tumors were collected and a slice of each one was fixated with 4% paraformaldehyde. The obtained slices were stained with hematoxylin and eosin stain or as terminal deoxynucleotidyl transferase dUTP nick end labeling stain. The histological images were taken using a CLSM (LSM-800, ZEISS, Germany).

### Proteomics analysis

Nine nude hepatocellular carcinoma patient-derived tumor-bearing mice were randomly separated into 3 groups, resulting in 3 mice for each group. The mice were intravenously injected in the tail vein with Comp-NPs (6 mg BODIPY kg^−1^). After 24 h, the tumor was exposed to irradiation (650 nm, 0.1 W cm^−2^, 60 J cm^−2^, 10 min). The tumors were collected and incubated in the absence and the presence of 10 μM PDS, respectively, at 37 °C for 24 h. The cells were then individually harvested, lysed on ice and extracted whole cell proteins by total protein extraction kit (BestBio). The concentration of raw protein extracts was measured by BCA Kit (Beyotime). The extracted proteins were digested and stable isotopic labeled. The labeled peptide mixtures were pre-fractionated by HPLC (Agilent Technologies 1260 infinity) with Agilent ZORBAX 300 Extend-C18 column. Mass spectrometric quantification was performed on an Orbitrap Fusion Lumos mass spectrometer coupled with an EASY-nLC 1200 nanoUPLC system equipped with an Acclaim™ PepMap™ 100 pre-column (20 mm × 75 μm, 3 μm) and an Acclaim™ PepMap™ RSLC C18 analytical column (150 mm × 75 μm, 2 μm). Raw MS/MS data were searched in Proteome Discoverer (Thermo Scientific, version 2.3) database for peptide and protein identification.

### Statistics and reproducibility

All statistical analyses were performed using GraphPad Prism. Data were analyzed using unpaired two-tailed *t* test, one-way ANOVA with Tukey’s multiple comparisons test and two-way ANOVA with Šídák’s multiple comparisons test for the calculation of *P* values. The number of replicates performed is indicated in each figure legend, where applicable.

### Reporting summary

Further information on research design is available in the Nature Portfolio Reporting Summary linked to this article.

### Supplementary information

Supplementary Information

Reporting Summary

### Source data

Source Data

## Data Availability

Proteomics data that support the findings of this study have been deposited in the Proteomics Identifications Database with accession code PXD041112. The source data underlying Figs. 2, 3, 4, 5, 6, 7, 8, 9, 10 and Supplementary Figs. 10, 12, 18, 20, 21, 22, 23, 24, 25, 27, 31, 32, 33, 34, 36, 39 and related western blot are provided with this paper. The authors declare that the remaining data supporting the findings of this study are available within the Article, its Supplementary Information or Source Data. Source data are provided with this paper.
