# Supplementary material for: Theranostic imaging and multimodal photodynamic therapy and immunotherapy using the mTOR signaling pathway
Source: Nat Commun. 2023 Sep 2;14:5350. doi: 10.1038/s41467-023-40826-5 (PMC10475087; doi:10.1038/s41467-023-40826-5)
Supplement: Supplementary file 1 — Supplementary Information [file 41467_2023_40826_MOESM1_ESM.pdf]

Supplementary Information:

Theranostic Imaging and Multimodal Photodynamic Therapy and Immunotherapy using the mTOR Signaling Pathway

TABLE OF CONTENTS

Supplementary Figure 1. Synthesis of the monomer (M1) 5

Supplementary Figure 2. Synthesis of the monomer (M2) 6

Supplementary Figure 3. ^{1}H-NMR spectrum of ethyl (*E*)-3-(4-(diphenylamino)phenyl)acrylate 2 in $CDCl_3$ 7

Supplementary Figure 4. ^{1}H-NMR spectra of ethyl 3-(4-(diphenylamino)phenyl)propanoate 3 in $CDCl_3$ 8

Supplementary Figure 5. ^{1}H NMR spectra of 3-(4-(diphenylamino)phenyl)propan-1-ol 4 in $CDCl_3$ 9

Supplementary Figure 6. ^{1}H NMR spectra of 4-(3-((tert-butyldimethylsilyl)oxy)propyl)-*N*,*N*-diphenylaniline 5 in $CDCl_3$ 10

Supplementary Figure 7. ^{1}H NMR spectra of 4-bromo-*N*-(4-(3-((tert-butyldimethylsilyl)oxy)propyl)phenyl)-*N*-phenylaniline 6 in $CDCl_3$ 11

Supplementary Figure 8. ^{1}H NMR spectra of 4-(3-((tert-butyldimethylsilyl)oxy)propyl)-N-phenyl-N-(4-(4,4,5,5-tetramethyl-1,3,2-dioxaborolan-2-yl)phenyl)aniline 7 in $CDCl_3$ 12

Supplementary Figure 9. Characterization of 4,4'-(6,7-bis(4-hexylphenyl)-[1,2,5]thiadiazolo[3,4-*g*]quinoxaline-4,9-diyl)bis(*N*-(4-(3-((tert-butyldimethylsilyl)oxy)propyl)phenyl)-*N*-phenylaniline) (compound 9) by NMR in $CDCl_3$ 13

Supplementary Figure 10. MALDI-TOF spectra of 9. Matrix: alpha-cyanocinnamic acid 14

Supplementary Figure 11. Characterization of 3,3'-((((6,7-bis(4-hexylphenyl)-[1,2,5]thiadiazolo[3,4-*g*]quinoxaline-4,9-diyl)bis(4,1-phenylene))bis(phenylazanediyl))bis(4,1-phenylene))bis(propan-1-ol) M1 by NMR in $CDCl_3$ 15

Supplementary Figure 12. MALDI-TOF spectra of M1. Matrix: alpha-cyanocinnamic acid 16

Supplementary Figure 13. ^{1}H-NMR spectra of 5,5-difluoro-1,3,7,9-tetramethyl-10-phenyl-5H--4λ4,5λ4-dipyrrolo[1,2-c:2',1'-f][1,3,2]diazaborinine (12) in DMSO-d_6. 17

Supplementary Figure 14. ^{1}H-NMR spectra of 5,5-difluoro-2,8-diiodo-1,3,7,9-tetramethyl-10-phenyl-5H-4λ4,5λ4-dipyrrolo[1,2-c:2',1'-f][1,3,2]diazaborinine (13) in DMSO-d_6. 18

Supplementary Figure 15. Characterization of 2,2'-((((1*E*,1'*E*)-(5,5-difluoro-2,8-diiodo-1,9-dimethyl-10-phenyl-5*H*-4λ^4,5λ^4-dipyrrolo[1,2-*c*:2',1'-*f*][1,3,2]diazaborinine-3,7-diyl)bis(ethene-2,1-diyl))bis(4,1-phenylene))bis(oxy))bis(ethan-1-ol) M2 by NMR in DMSO-d_6 19

Supplementary Figure 16. Synthesis and characterization of P1 20

Supplementary Figure 17. Synthesis and characterization of P2 21

Supplementary Figure 18. UV-vis absorption spectra of monomers, polymers and nanoparticles 22

Supplementary Figure 19. Representative transmission electron microscope images of NP1 (P1: 0.5 mg/mL) and NP2 (P2: 0.5 mg/mL) upon addition of H_2O_2 (10 mM) 23

Supplementary Figure 20. Change in absorption at 410 nm corresponding to the ROS specific probe 1,3-diphenylisobenzofuran (DPBF) upon incubation of Comp-NPs and irradiation (L, 650 nm, 0.1 W cm^{-2}, 60 J cm^{-2} , 10 min) 24

Supplementary Figure 21. Phosphorescence spectra of Comp-NPs encapsulated with the dye Nile Red (λex = 550 nm) upon irradiation (650 nm, 0.1 W cm^{-2}) 25

Supplementary Table 1. Determination of the average size and polydispersity of NP1, NP2, and Comp-NPs in water 26

Supplementary Figure 22.The cellular uptake of Comp-NPs by 4T1 cells and 4T1 multicellular tumor spheroids 27

Supplementary Figure 23. ROS generation of Comp-NPs (0.5 μg/mL BODIPY) in 4T1 cells after various incubation times by flow cytometry 28

Supplementary Figure 24. *In vitro* anticancer activity test of nanoparticles and cisplatin 29

Supplementary Table 2. IC_{50} values of BODIPY of NP1, NP2, Comp-NPs in μg/mL, and cisplatin (CisPt) μM in the dark or upon irradiation (650 nm, 0.1 W cm^{-2}, 60 J cm-^{2}, 10 min) in 4T1, SKOV3, A2780 and A2780DDP cells 30

Supplementary Figure 25. In vitro anticancer activity test of nanoparticles with various ratios of NP1 and NP2. Relative cell viability of 4T1 cells upon treatment with NP1, NP2, Comp-NPs with ratio of NP1 to NP2 is (a) 1:2, (b) 2:1, (c) 1:3, (d) 3:1 in the dark or upon irradiation (L, 650 nm, 0.1 W cm^{-2}, 60 J cm^{-2}, 10 min). 31

Supplementary Table 3. IC_{50} values of Comp-NPs with different ratios of NP1 and NP2 in the dark or upon irradiation (650 nm, 0.1 W cm^{-2}, 60 J cm^{-2}, 10 min) in 4T1 cells. 32

Supplementary Figure 26. Analysis of the cell death of 4T1 cells upon treatment with NP1, NP2, and Comp-NPs, in the dark or upon irradiation (L, 650 nm, 0.1 W cm^{-2}, 30 J cm^{-2}, 5 min). 33

Supplementary Figure 27. Western Blot of the caspase-3 protein expression levels in 4T1 cells after treatment with NP1, NP2, and Comp-NPs, in the dark or upon irradiation (L, 650 nm, 0.1 W cm^{-2}, 30 J cm^{-2}, 5 min). 34

Supplementary Figure 28. Light microscopy images of 4T1 cancer cells upon incubation with phosphate-buffered saline or Comp-NPs upon irradiation (L, 650 nm, 0.1 W cm^{-2}, 30 J cm^{-2}, 5 min) 35

Supplementary Figure 29. CLSM images of 4T1 incubated with HMGB1 protein fluorescent probe (green) and DAPI (blue) upon various treatments 36

Supplementary Figure 30. Cell migration wound healing assay of 4T1 cells upon various treatments in the light (650 nm, 0.1 W cm-2, 30 J cm-2, 5 min). 37

Supplementary Figure 31. Flow cytometry examination of DCs maturation upon various treatments in the dark. 38

Supplementary Figure 32. *In vivo* systemic toxicities study 39

Supplementary Figure 33. Semi-quantifications of mean fluorescence intensity of Figure 4B 40

Supplementary Figure 34. In vivo anticancer efficacy on 4T1 tumor-bearing mice. 41

Supplementary Figure 35. Photographs of the animals after various treatments. 43

Supplementary Figure 36. *In vitro* anticancer activity test and ICD effect of Comp-NPs on JHH7 cells. 44

Supplementary Figure 37. H&E staining of organs upon various treatments of HCC tumor bearing mice. 45

Supplementary Figure 38. Proteomic KEGG pathway enrichment analysis upon different treatments 46

Supplementary Figure 39. Western Blot of the mTOR and P70S6K protein expression levels in 4T1 cells after treatment with NP1, NP2, and Comp-NPs upon irradiation (L, 650 nm, 0.1 W cm^{-2}, 30 J cm^{-2}, 5 min). 47

Supplementary Figure 40. The gating strategies of flow cytometry analyses for CRT positive cells, Matured DCs in vitro, Matured DCs in vivo, $CD3^+CD4^+CD8^+$ T cells, IFN-γ^+ $CD8^+$ T cells. 48

Supplementary Figure 41. The gating strategies of flow cytometry analyses for $CD3^+CD8^+$ T cells, Cellular uptake, ROS generation, Apoptosis cells. 49

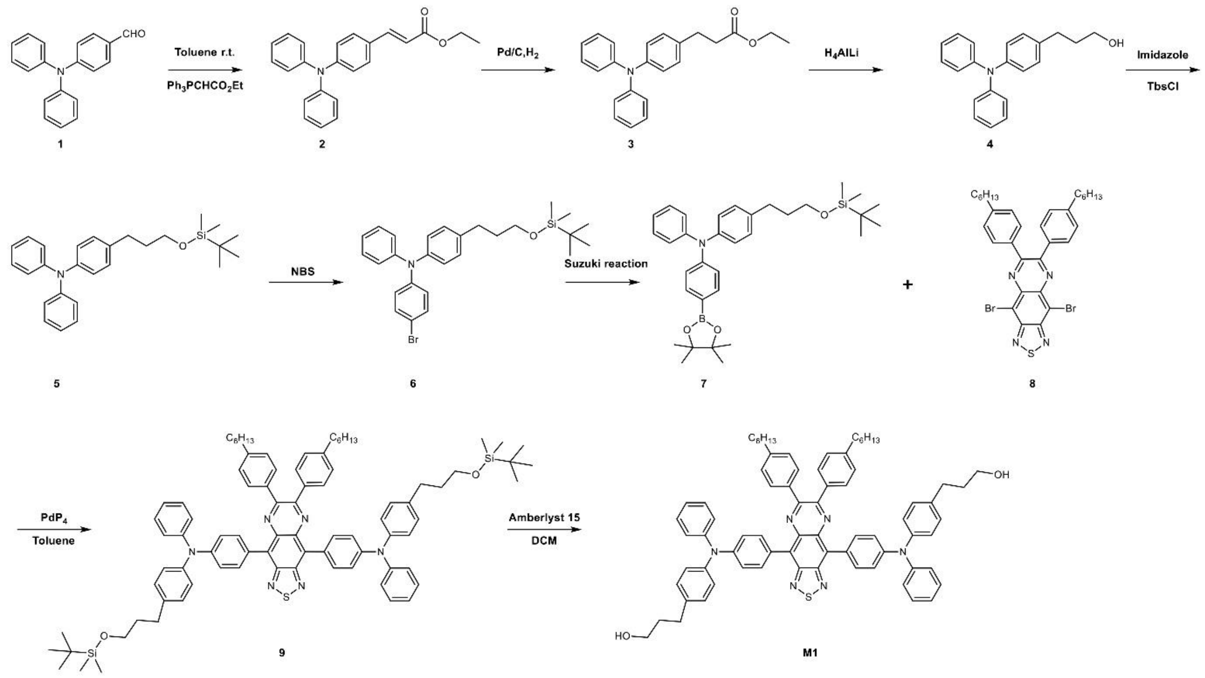

Supplementary Figure 1. Synthesis of the monomer (M1).

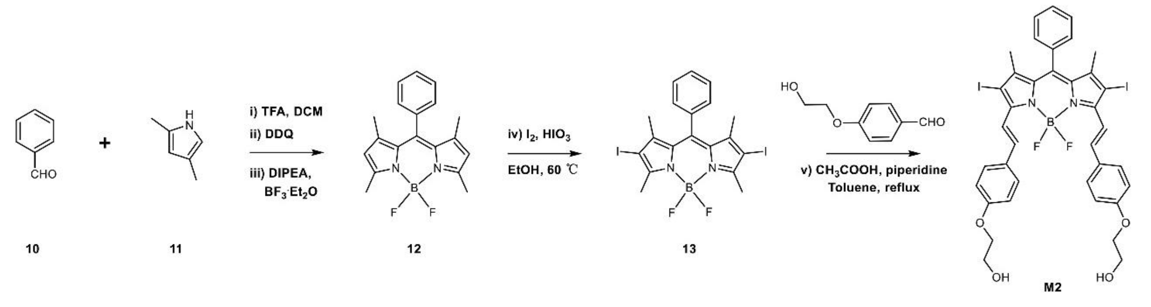

Supplementary Figure 2. Synthesis of the monomer (M2).

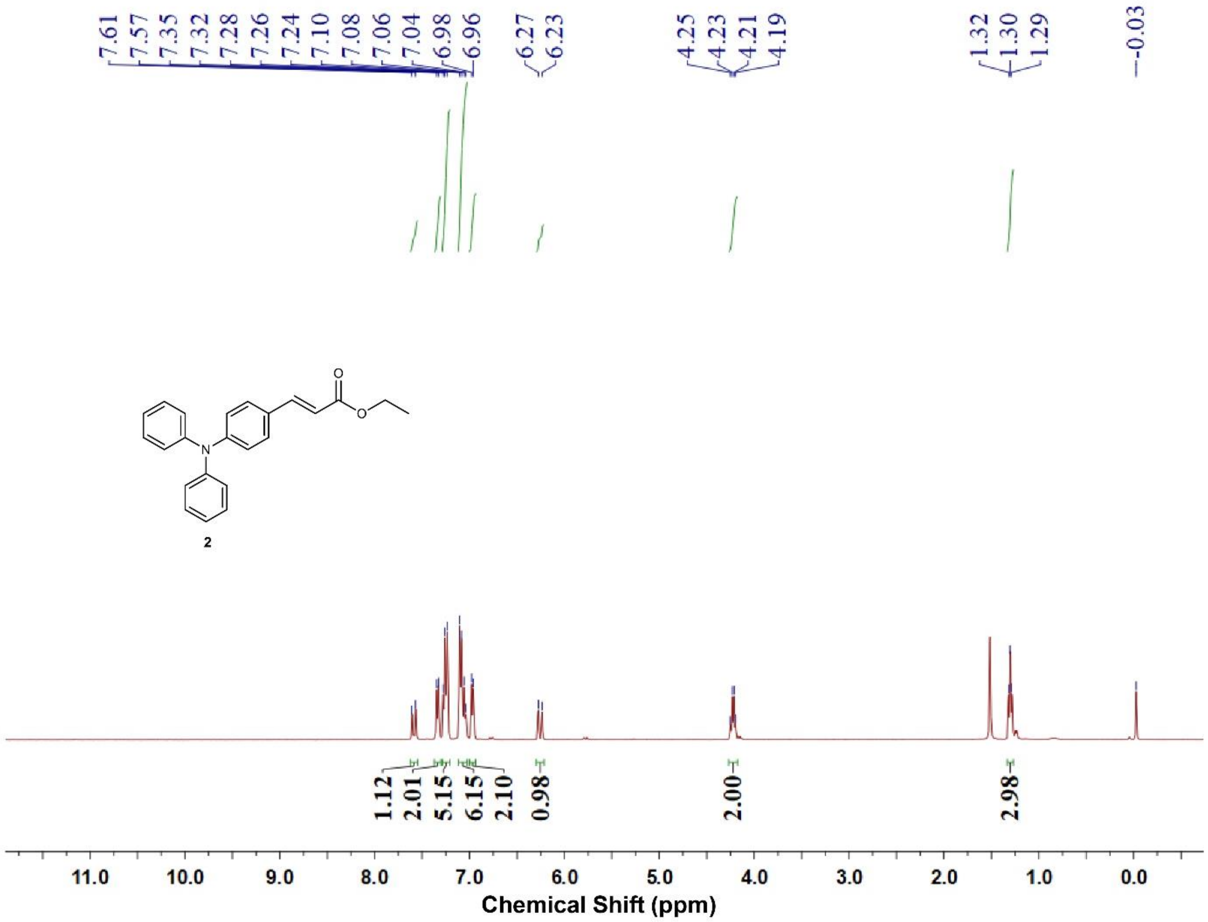

Supplementary Figure 3. ^{1}H-NMR spectrum of ethyl (*E*)-3-(4-(diphenylamino)phenyl)acrylate 2 in $CDCl_3$.

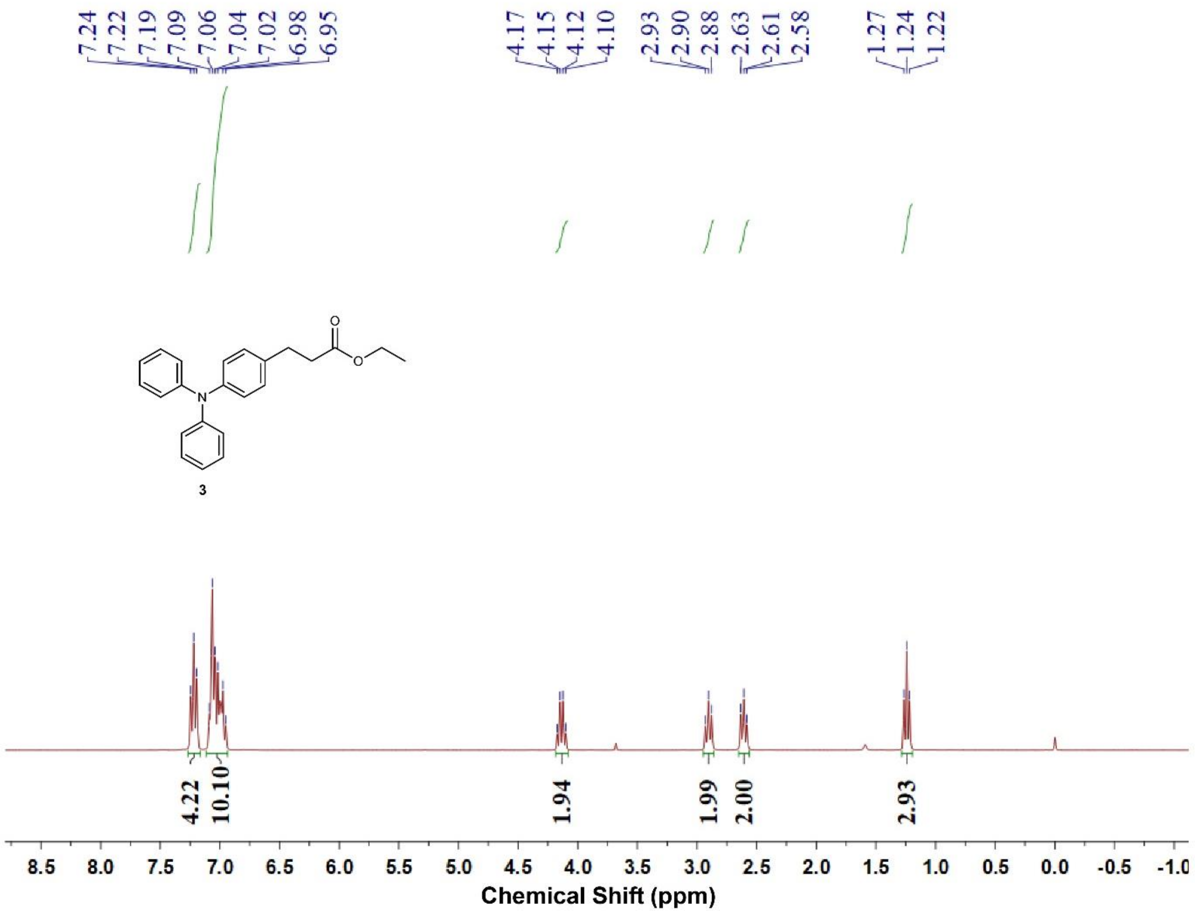

Supplementary Figure 4. ^{1}H-NMR spectra of ethyl 3-(4-(diphenylamino)phenyl)propanoate 3 in $CDCl_3$.

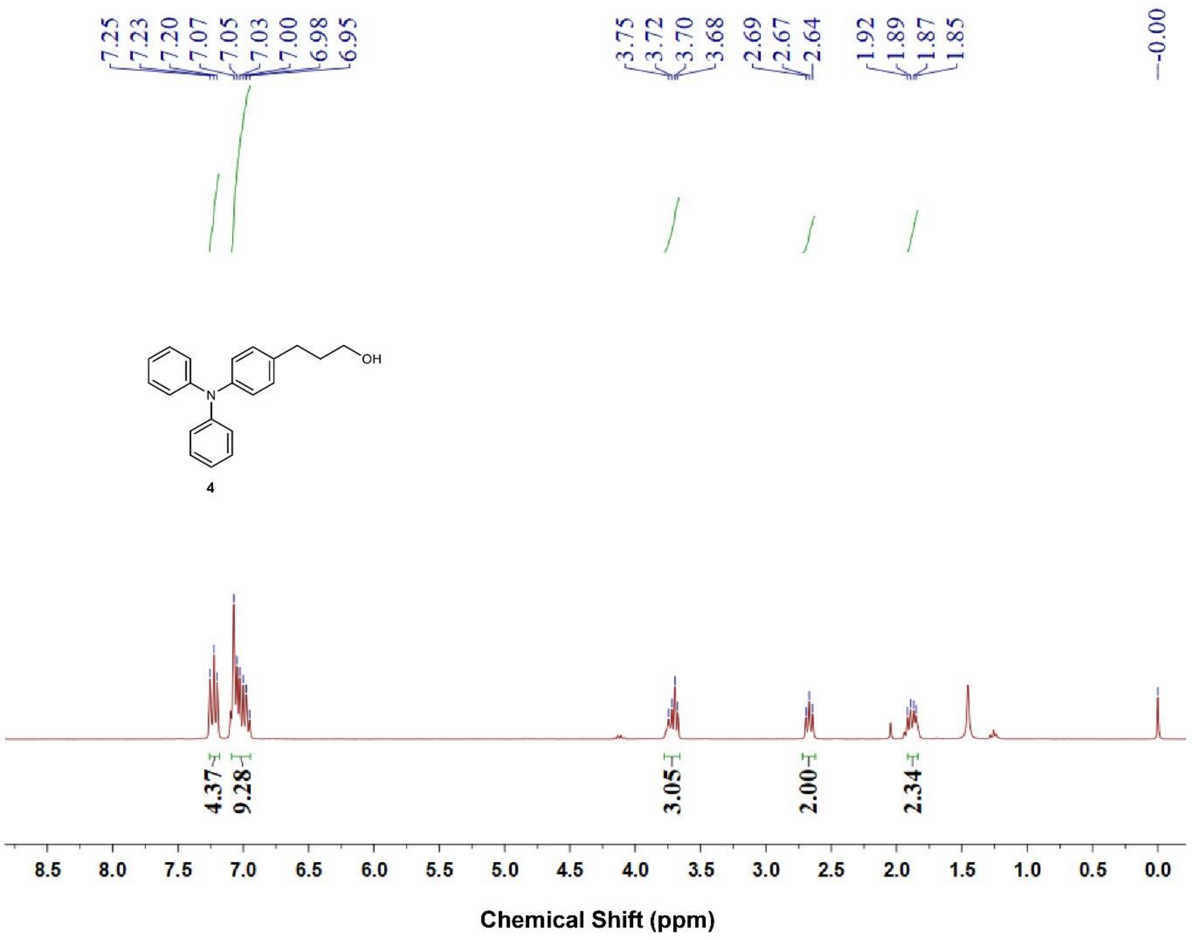

Supplementary Figure 5. ^{1}H-NMR spectra of 3-(4-(diphenylamino)phenyl)propan-1-ol 4 in $CDCl_3$.

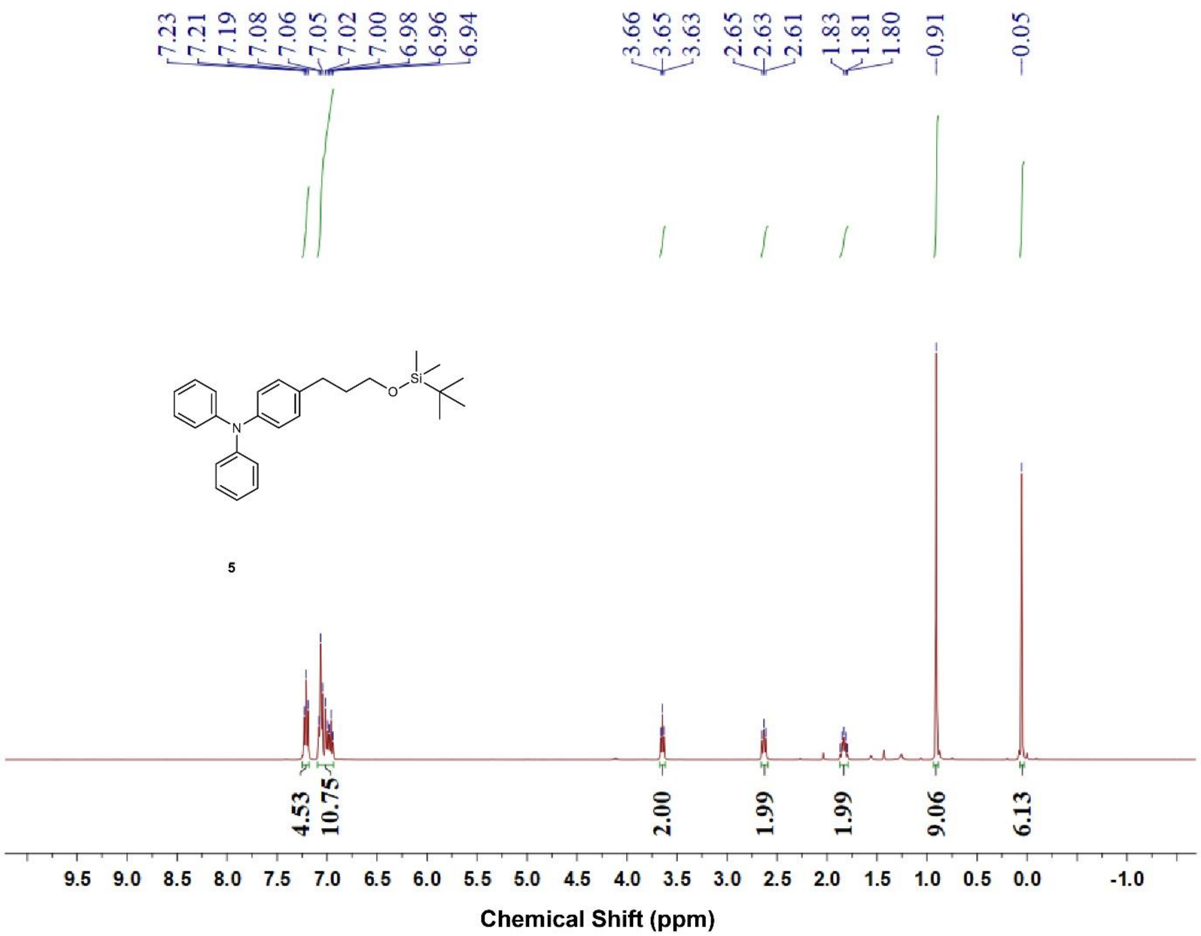

Supplementary Figure 6. ^{1}H-NMR spectra of 4-(3-((tert-butyldimethylsilyl)oxy)propyl)-*N*,*N*-diphenylaniline 5 in $CDCl_3$.

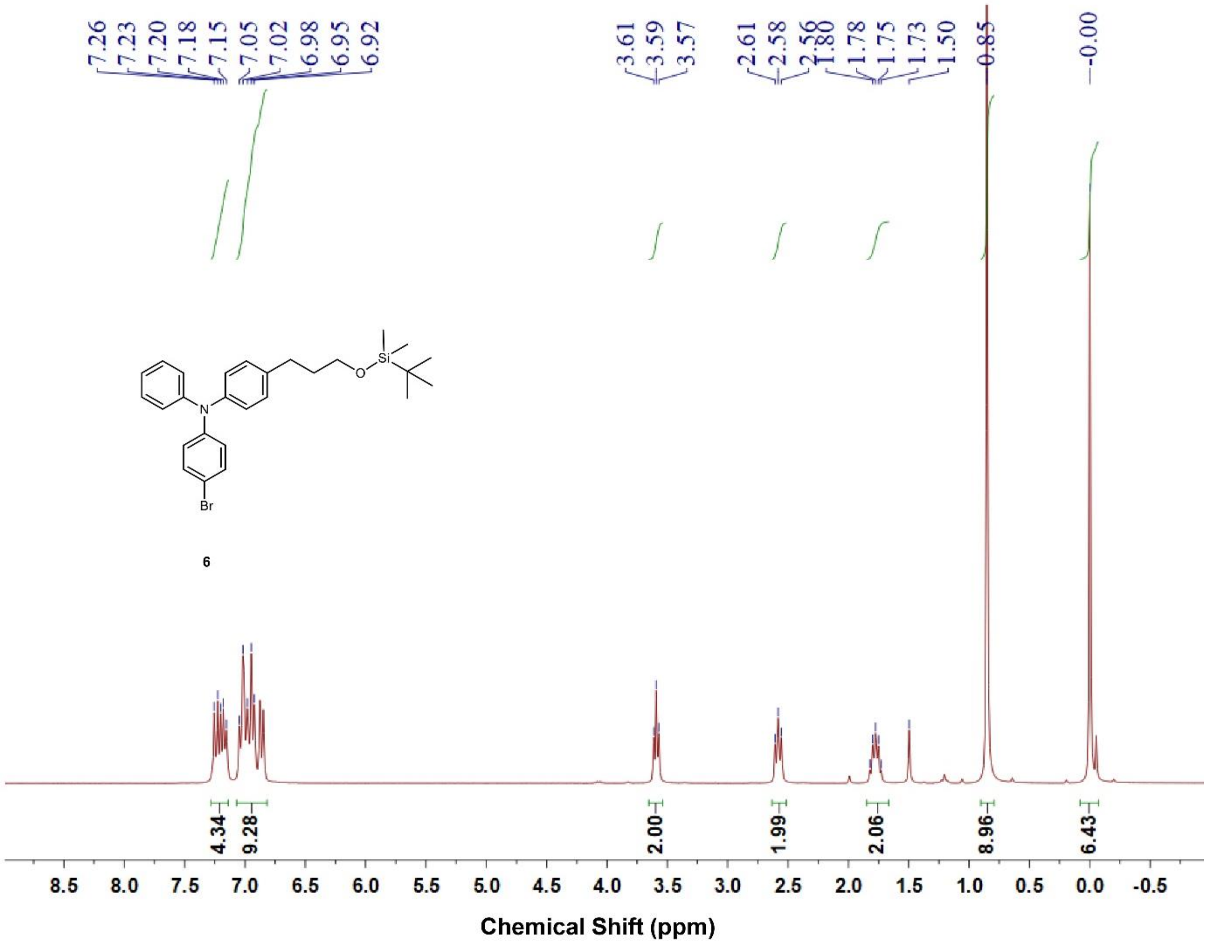

Supplementary Figure 7. ^{1}H-NMR spectra of 4-bromo-*N*-(4-(3-((tert-butyldimethylsilyl)oxy)propyl)phenyl)-*N*-phenylaniline 6 in $CDCl_3$.

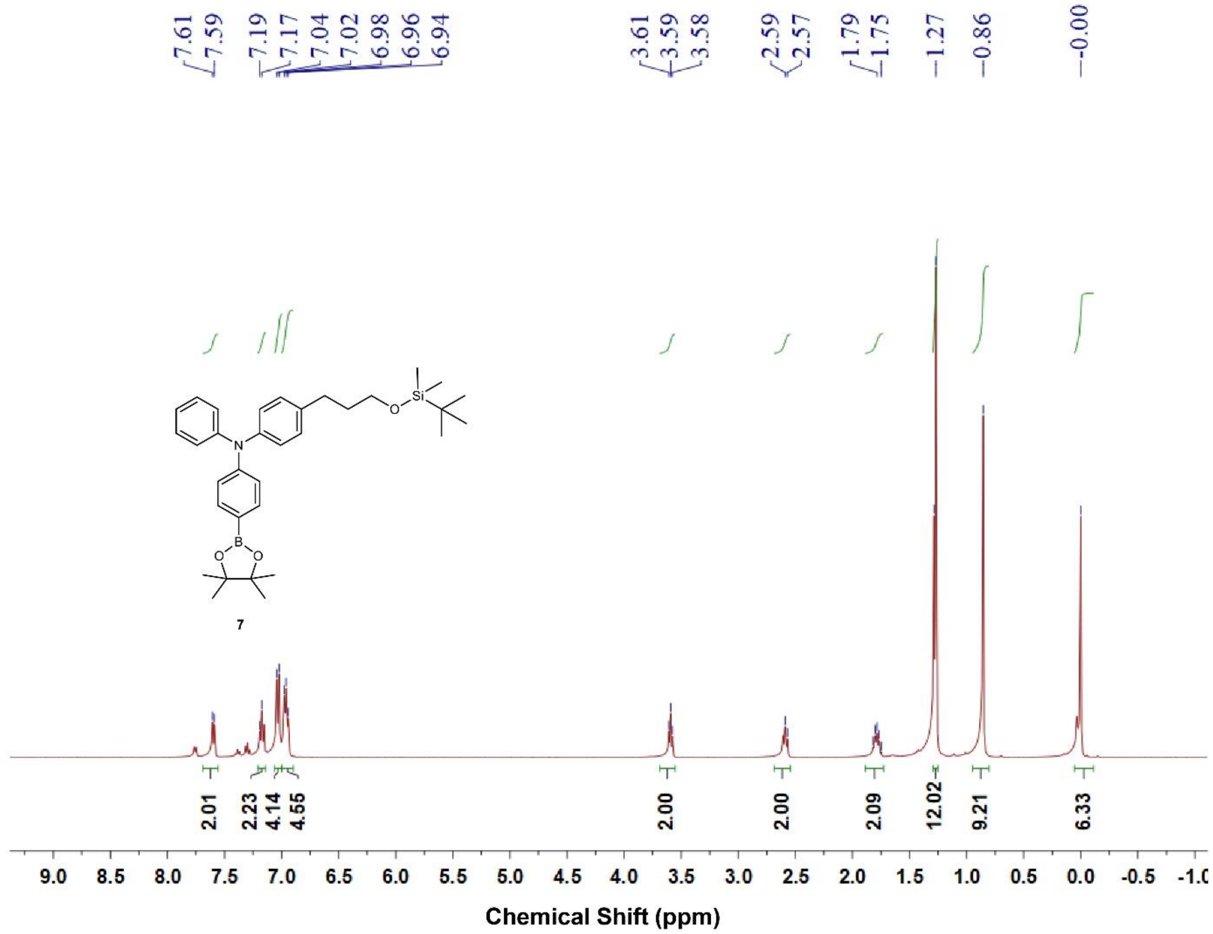

Supplementary Figure 8. ^{1}H-NMR spectra of 4-(3-((tert-butyldimethylsilyl)oxy)propyl)-*N*-phenyl-N-(4-(4,4,5,5-tetramethyl-1,3,2-dioxaborolan-2-yl)phenyl)aniline 7 in $CDCl_3$.

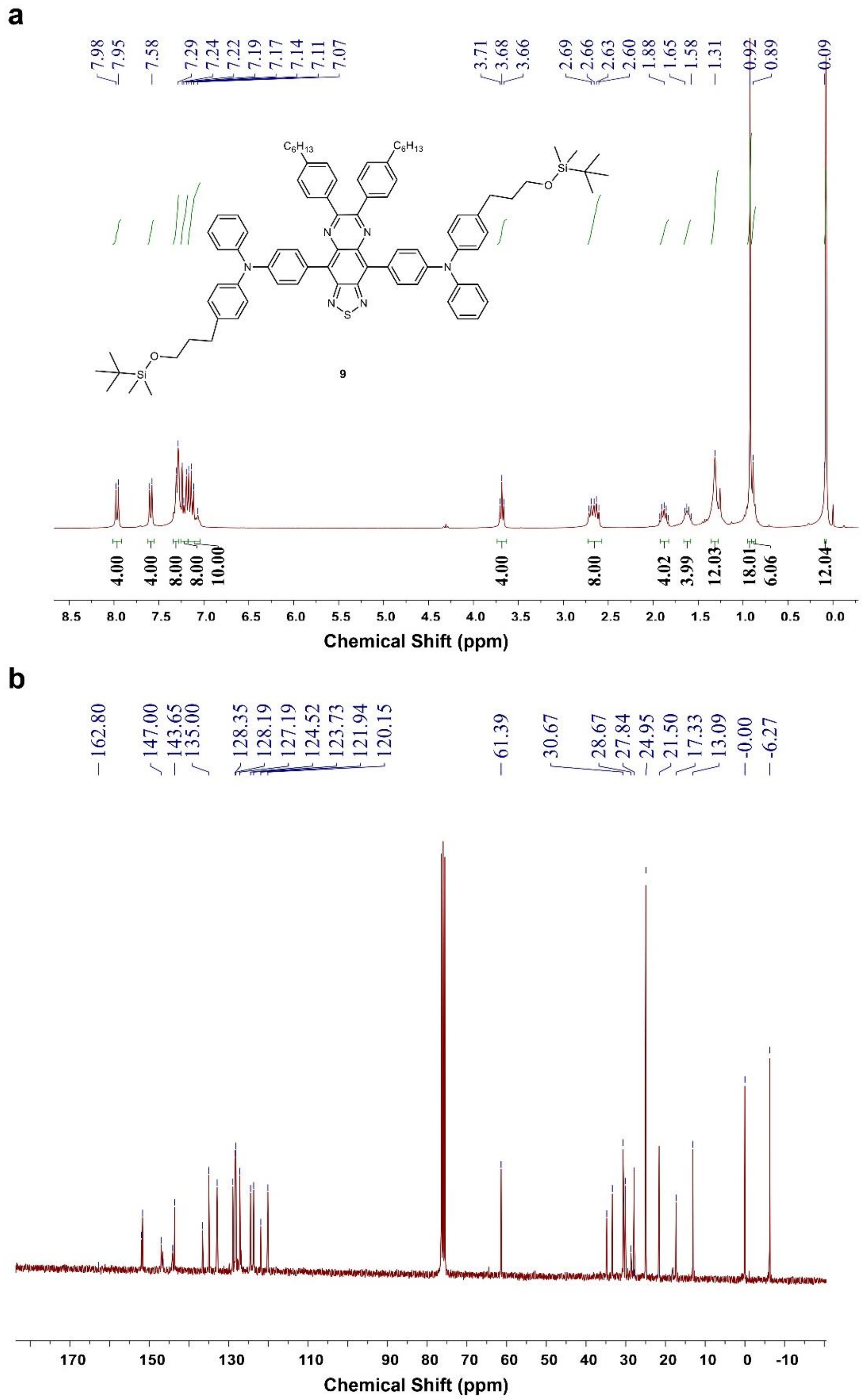

Supplementary Figure 9. Characterization of 4,4'-(6,7-bis(4-hexylphenyl)-[1,2,5]thiadiazolo[3,4-*g*]quinoxaline-4,9-diyl)bis(*N*-(4-(3-((tert-butyldimethylsilyl)oxy)propyl)phenyl)-*N*-phenylaniline) 9 by NMR in $CDCl_3$. (a) ^{1}H-NMR and (b) ^{13}C-NMR of compound 9.

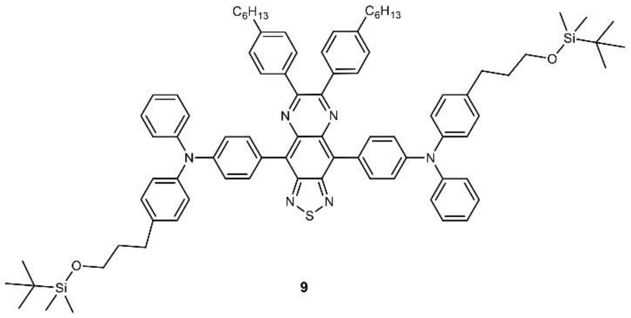

9

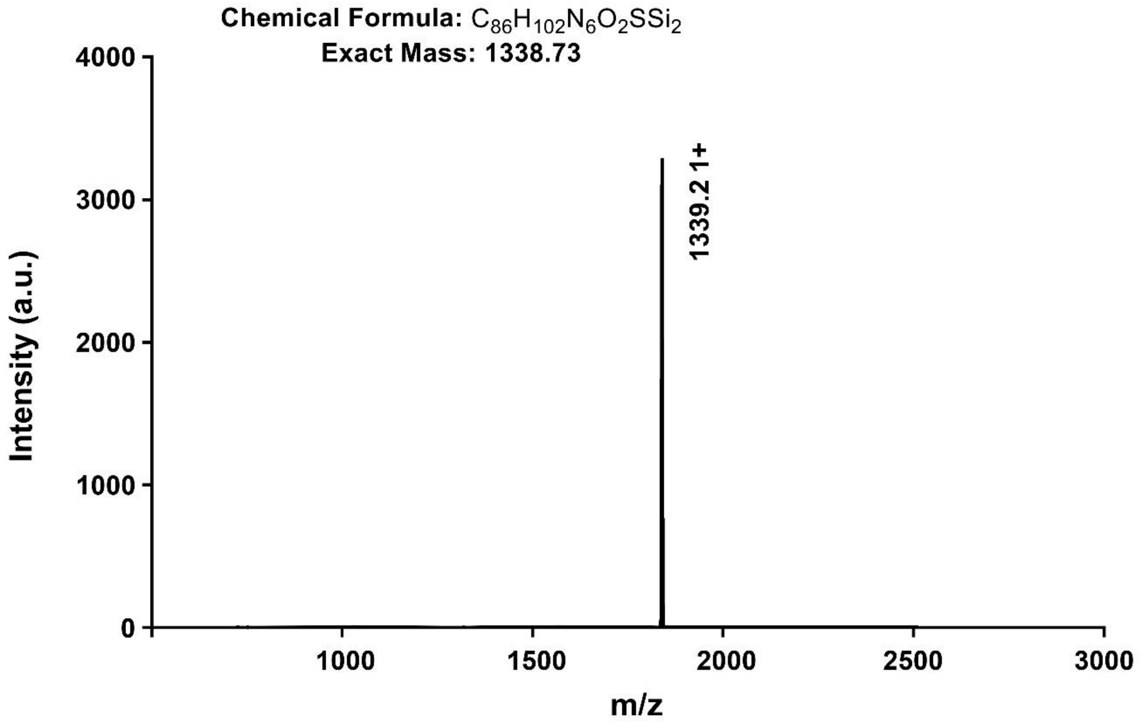

Supplementary Figure 10. MALDI-TOF spectra of 9. Matrix: alpha-cyanocinnamic acid.

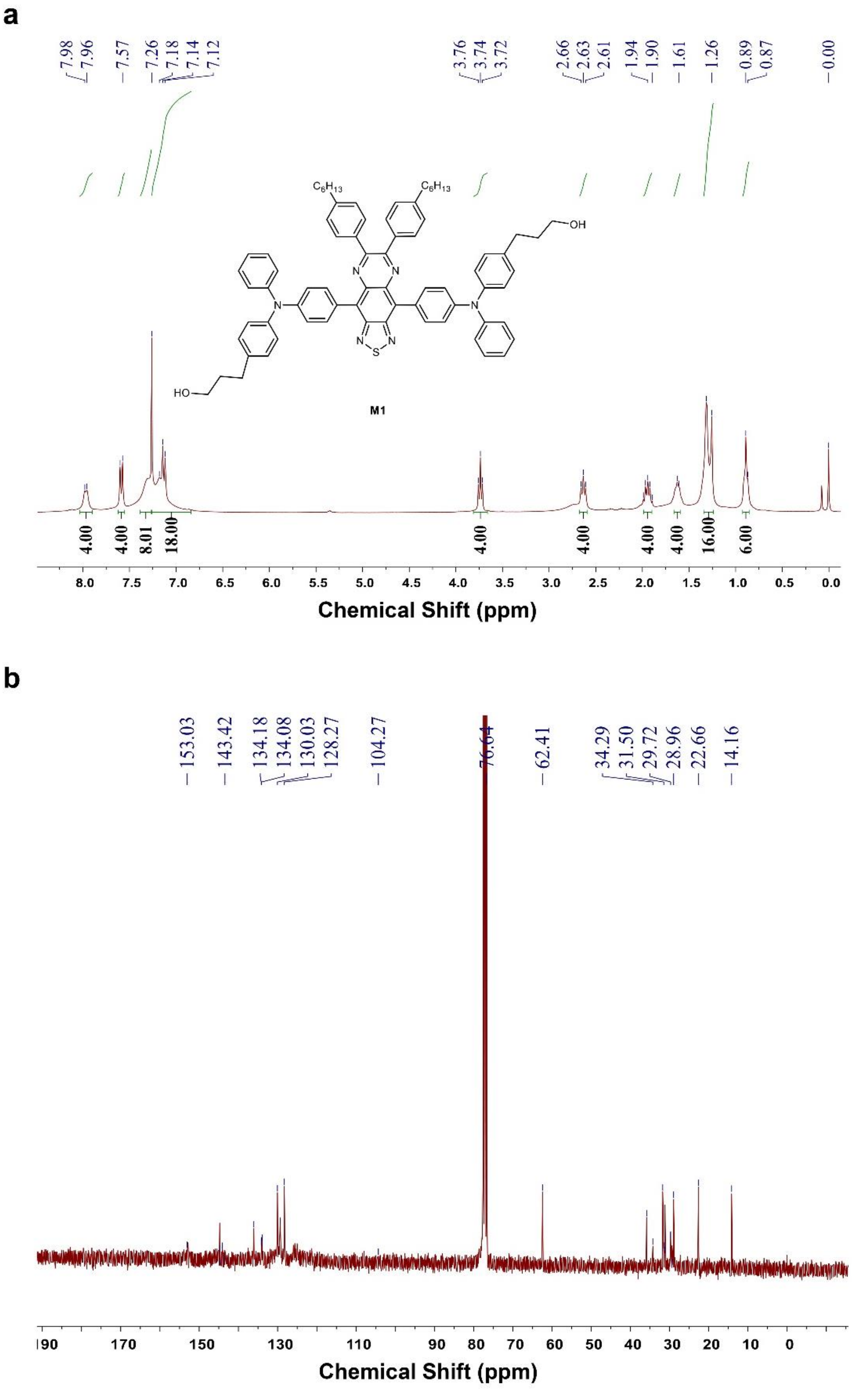

Supplementary Figure 11. Characterization of 3,3'-((((6,7-bis(4-hexylphenyl)-[1,2,5]thiadiazolo[3,4-*g*]quinoxaline-4,9-diyl)bis(4,1-phenylene))bis(phenylazanediyl))bis(4,1-phenylene))bis(propan-1-ol) M1 by NMR in $CDCl_3$. (a) ^{1}H-NMR and (b) ^{13}C-NMR of M1.

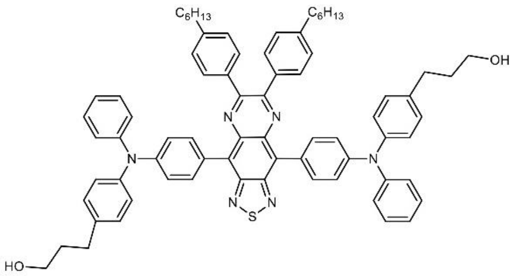

M1

Chemical Formula: $C_{74}H_{74}N_6O_2S$
Exact Mass: 1110.56
Molecular Weight: 1111.51

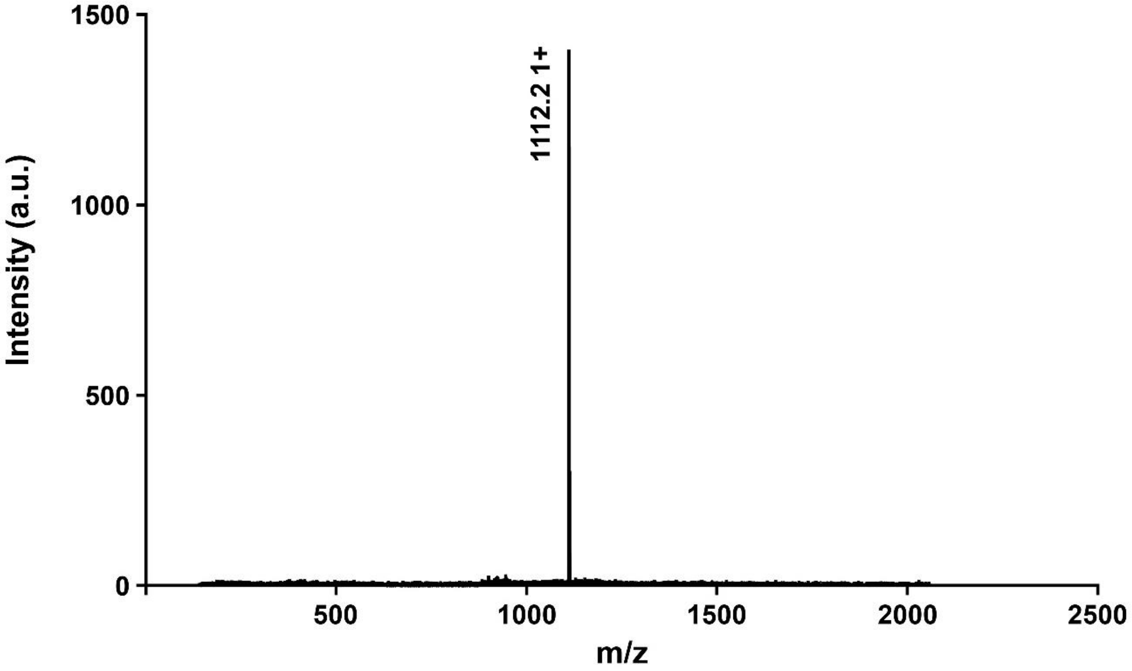

Supplementary Figure 12. MALDI-TOF spectra of M1. Matrix: alpha-cyanocinnamic acid.

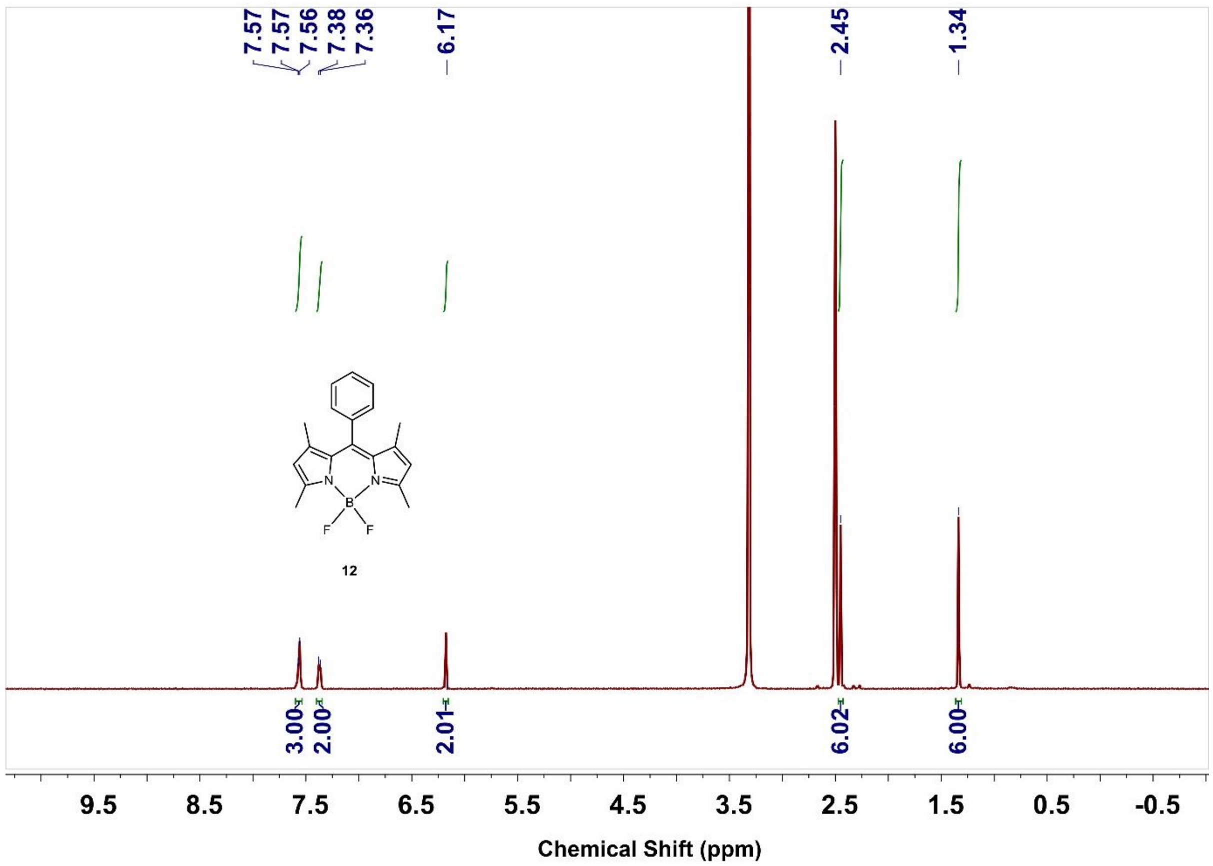

Supplementary Figure 13. ^{1}H-NMR spectra of 5,5-difluoro-1,3,7,9-tetramethyl-10-phenyl-5*H*--4λ^{4},5λ^{4}-dipyrrolo[1,2-*c*:2',1'-*f*][1,3,2]diazaborinine 12 in DMSO-d_6.

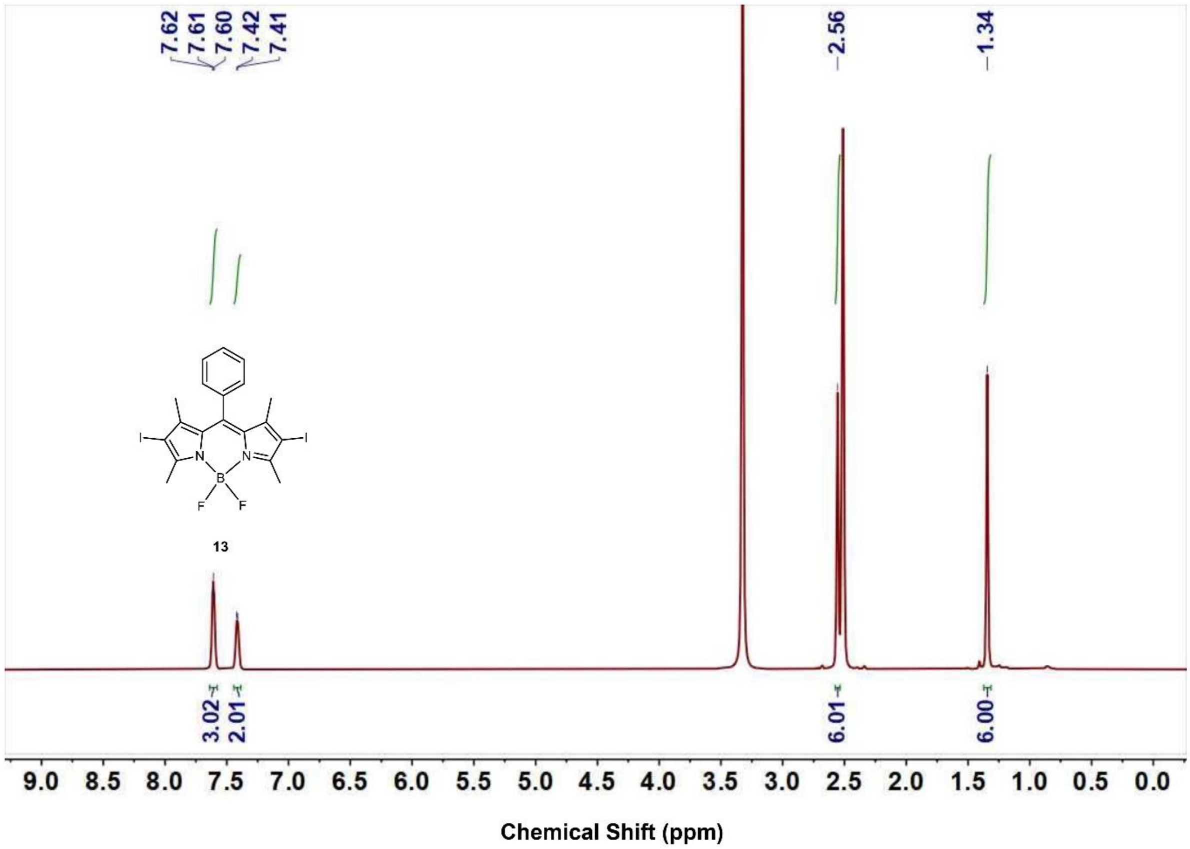

Supplementary Figure 14. ^{1}H-NMR spectra of 5,5-difluoro-2,8-diiodo-1,3,7,9-tetramethyl-10-phenyl-5*H*-4λ^{4},5λ^{4}-dipyrrolo[1,2-*c*:2',1'-*f*][1,3,2]diazaborinine 13 in DMSO-d_6.

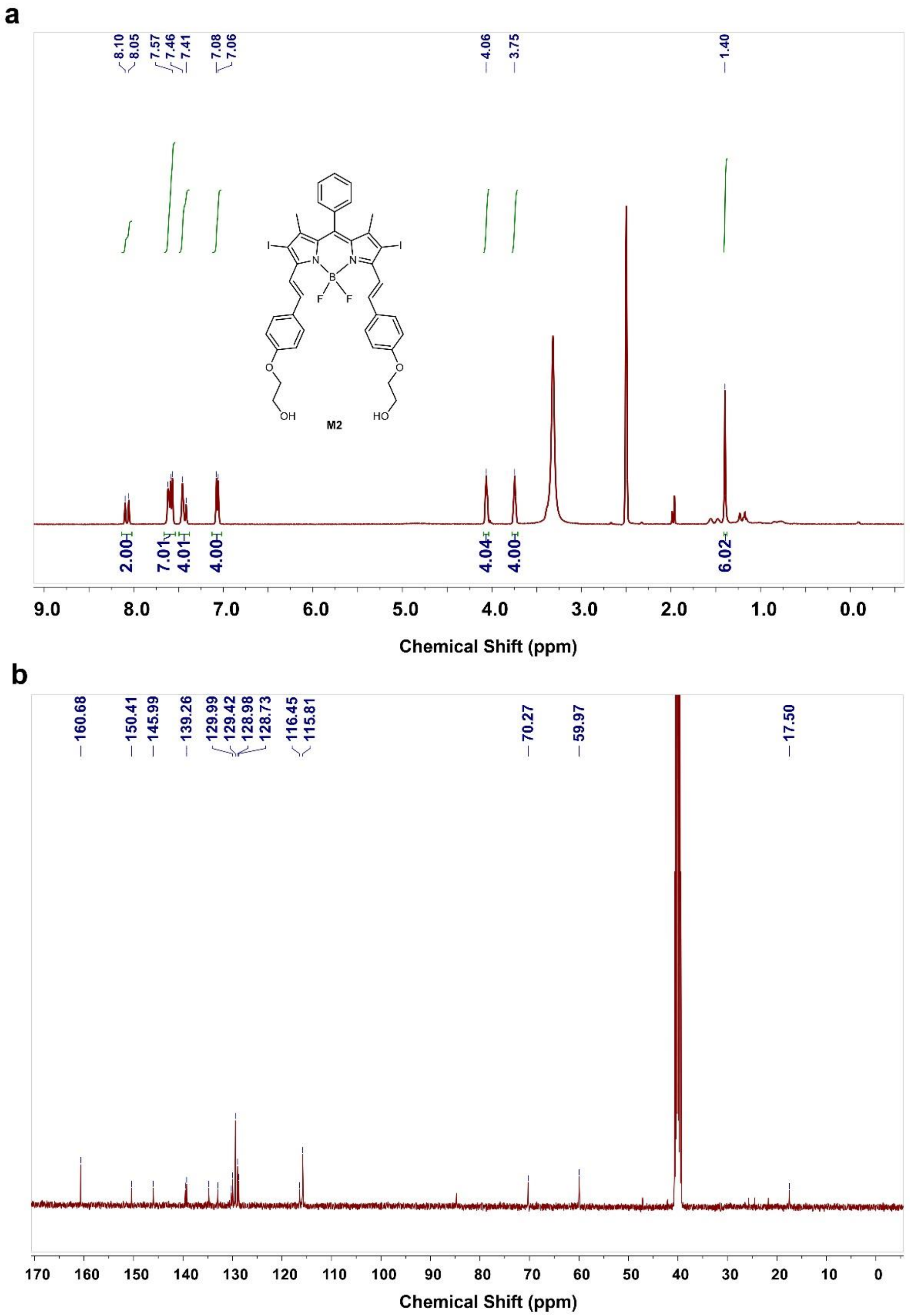

Supplementary Figure 15. Characterization of 2,2'-((((1*E*,1'*E*)-(5,5-difluoro-2,8-diiodo-1,9-dimethyl-10-phenyl-5*H*-4λ^4,5λ^4-dipyrrolo[1,2-*c*:2',1'-*f*][1,3,2]diazaborinine-3,7-diyl)bis(ethene-2,1-diyl))bis(4,1-phenylene))bis(oxy))bis(ethan-1-ol) M2 by NMR in DMSO-d_6. (a) ^{1}H-NMR and (b) ^{13}C-NMR of M2.

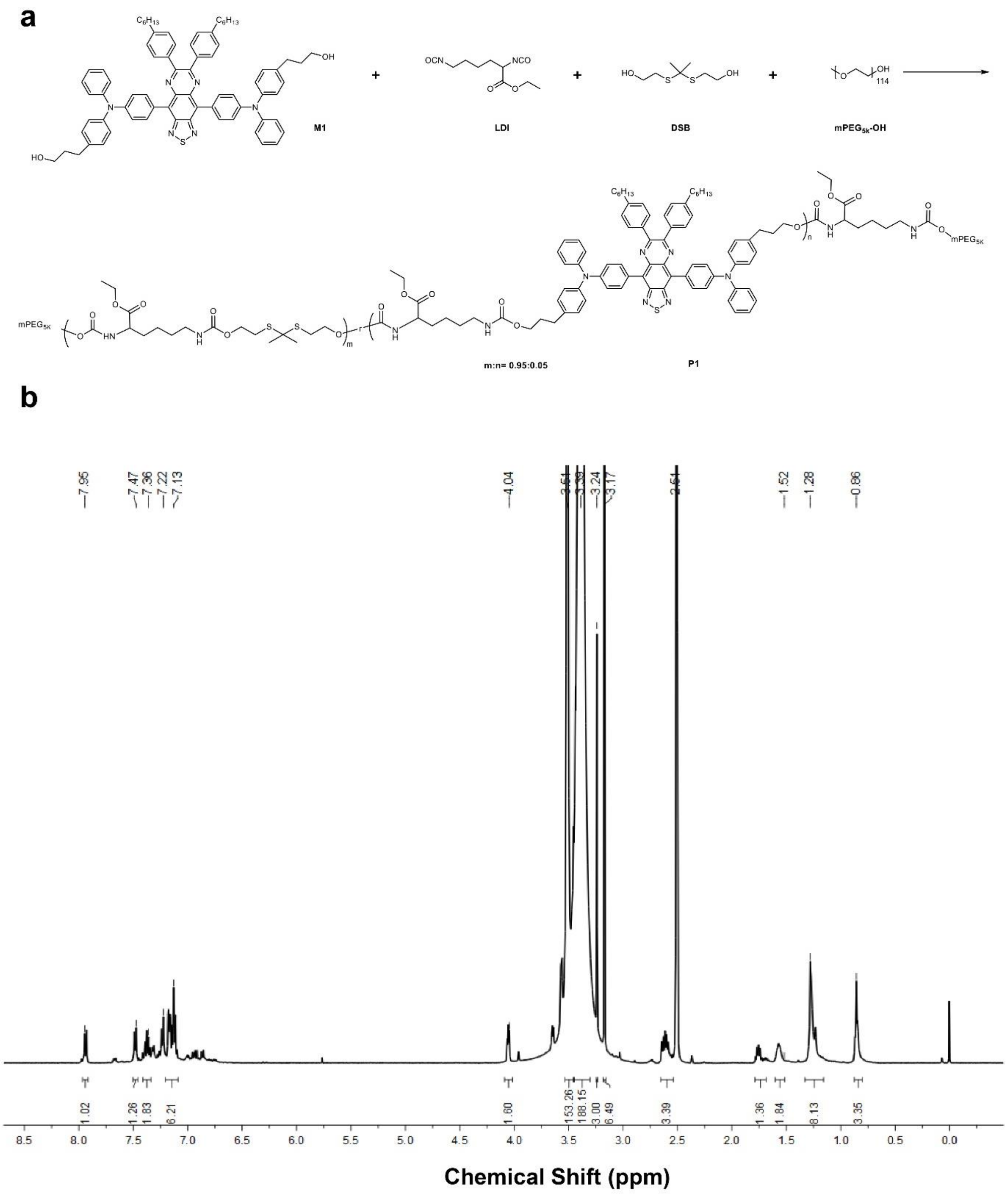

Supplementary Figure 16. Synthesis and characterization of P1. (a) Synthetic scheme for P1. (b) ^{1}H-NMR of P1 in DMSO-d_6.

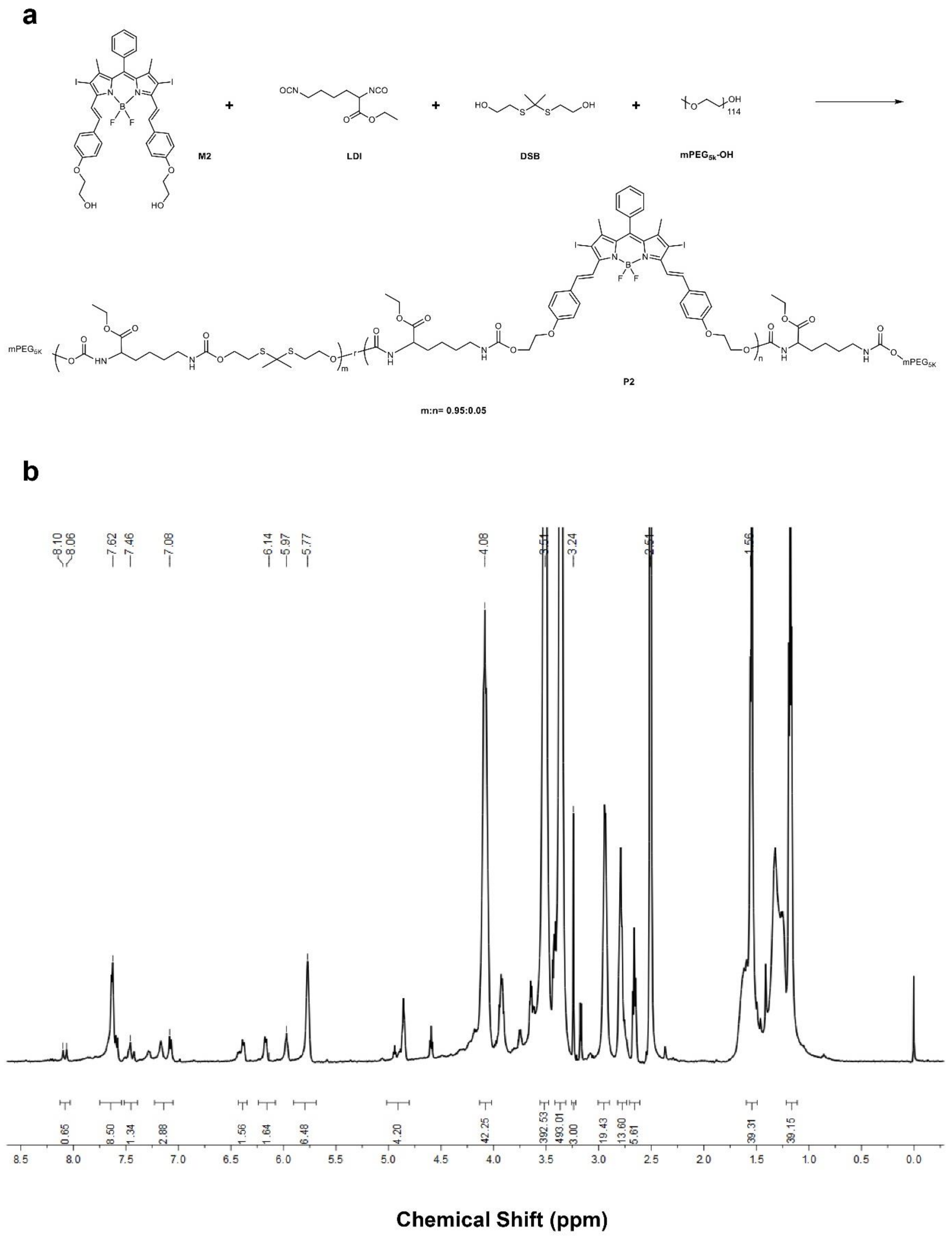

Supplementary Figure 17. Synthesis and characterization of P2. (a) Synthetic scheme for P2. (b) ^{1}H-NMR of P2 in DMSO-d_6.

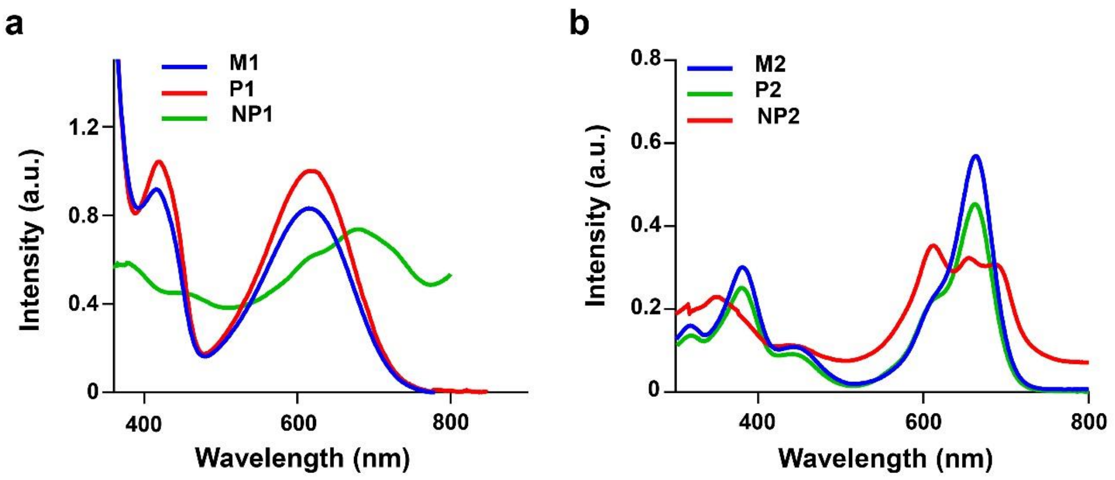

Supplementary Figure 18. UV-vis absorption spectra of monomers, polymers and nanoparticles. (a) UV-vis absorption spectra of M1 in DCM (370 μg/mL), P1 in DCM (4 mg/mL), and NP1 in water. (b) UV-vis absorption spectra of M2 in DCM (15 μg/mL), P2 in DCM (75 μg/mL), and NP2 in water.

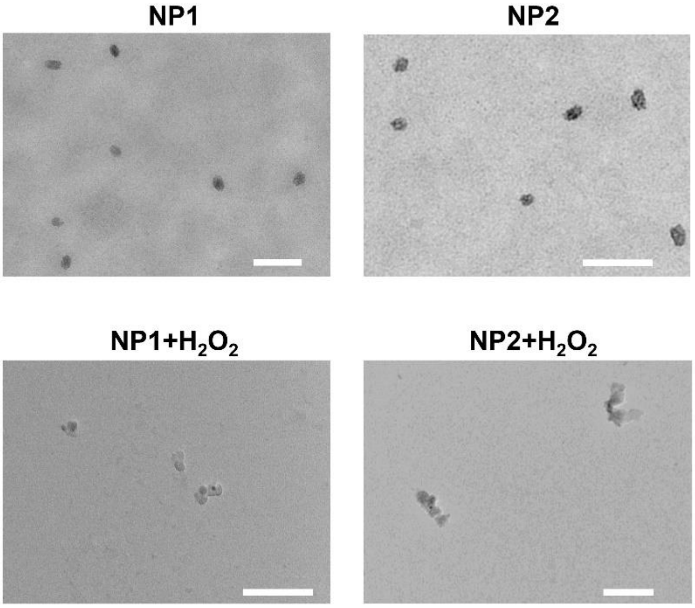

Supplementary Figure 19. Representative transmission electron microscope images of NP1 (P1: 0.5 mg/mL) and NP2 (P2: 0.5 mg/mL) upon addition of H_2O_2 (10 mM). The experiment was repeated independently 3 times with similar results. Scale bar = 200 nm.

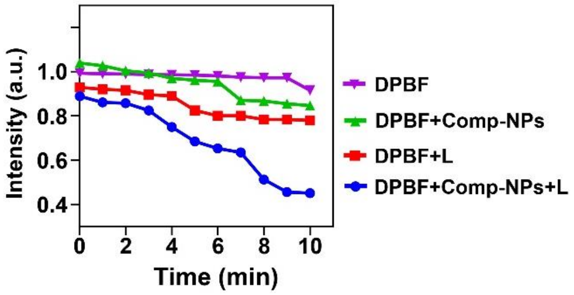

Supplementary Figure 20. Change in absorption at 410 nm corresponding to the ROS specific probe 1,3-diphenylisobenzofuran (DPBF) upon incubation of Comp-NPs and irradiation (L, 650 nm, 0.1 W cm^{-2}, 60 J cm^{-2} ,10 min). (n = 3 independent samples)

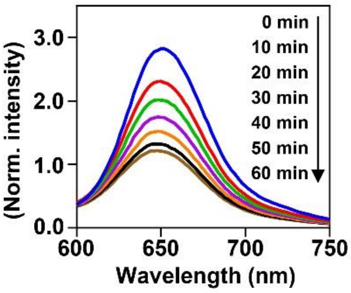

Supplementary Figure 21. Phosphorescence spectra of Comp-NPs encapsulated with the dye Nile Red (λ_{ex} = 550 nm) upon irradiation (650 nm, 0.1 W cm^{-2}). (n = 3 independent samples)

Supplementary Table 1. Determination of the average size and polydispersity of NP1, NP2, and Comp-NPs in water.

Samples	Z-Average size (nm)	Polydispersity
NP1	94.3	0.218
NP2	91.8	0.251
Comp-NPs	102.9	0.212

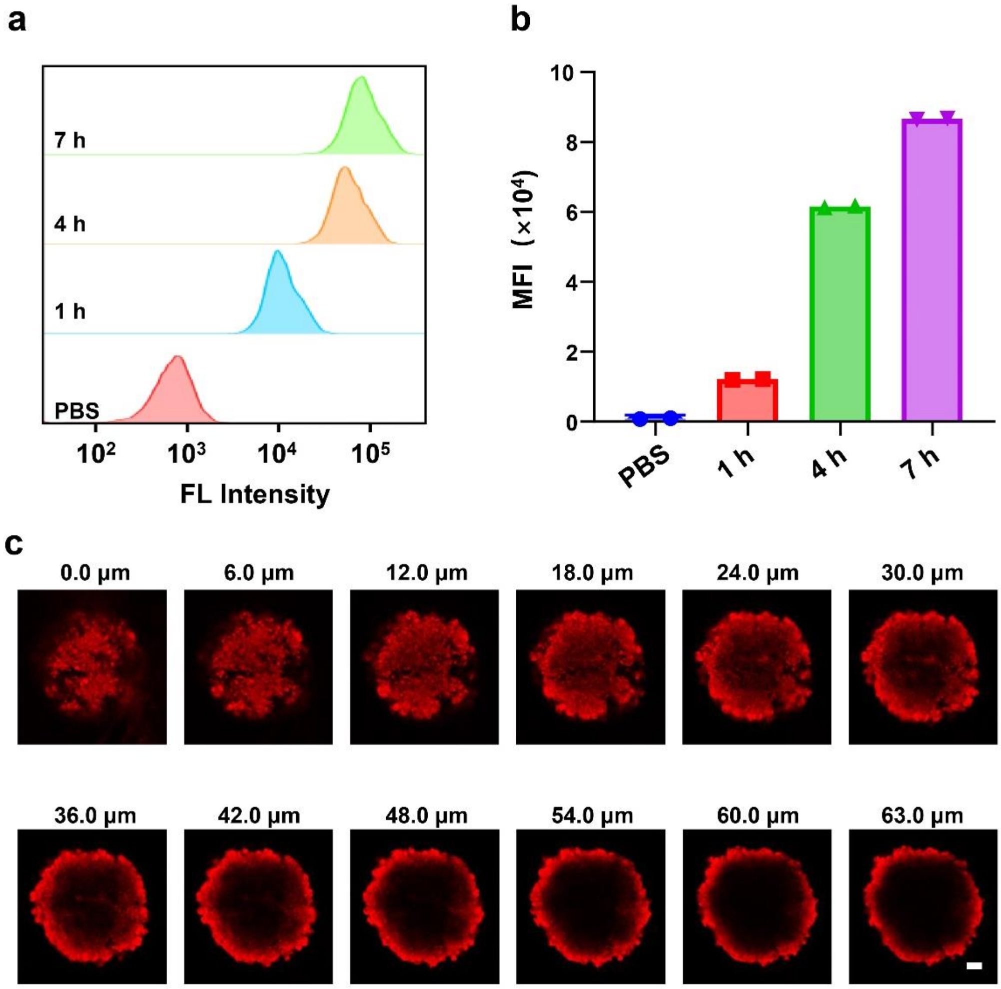

Supplementary Figure 22. The cellular uptake of Comp-NPs by 4T1 cells and 4T1 multicellular tumor spheroids. (a) The cellular uptake of Comp-NPs (1 μg/mL BODIPY) in 4T1 cells after various incubation times by flow cytometry. (b) Quantification of the cellular uptake from (a) (n = 2 biologically independent samples). (c) Z-stack CLSM images of Comp-NPs (1 μg/mL BODIPY) in 4T1 multicellular tumor spheroids after 7 h. The experiment was repeated independently 3 times with similar results. Comp-NPs: λ_{ex} = 650 nm, λ_{em} = 745 nm. Scale bar = 100 μm.

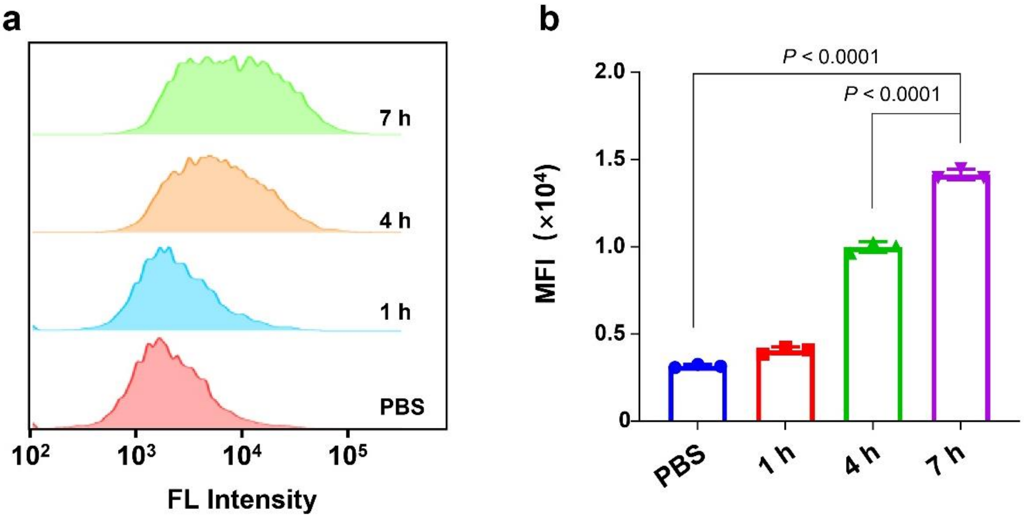

Supplementary Figure 23. ROS generation of Comp-NPs (0.5 μg/mL BODIPY) in 4T1 cells after various incubation times by flow cytometry. (a) Histogram of ROS generation of Comp-NPs by flow cytometry. (b) Semi-quantification of ROS generation of Comp-NPs by flow cytometry (*n* = 3 biologically independent samples). Error bars represent mean ± SD. Statistical analysis was performed by two-tailed unpaired *t*-test.

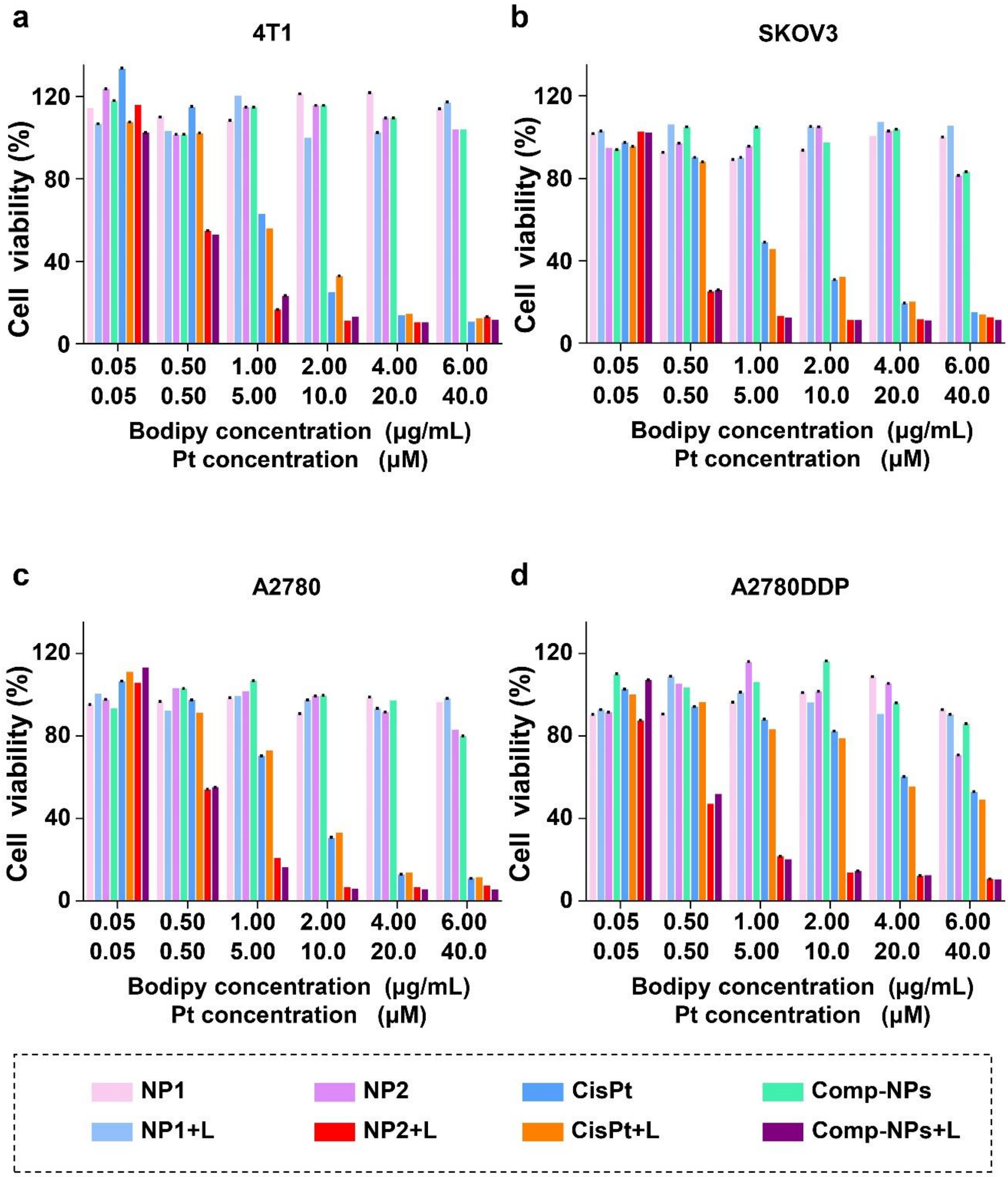

Supplementary Figure 24. *In vitro* anticancer activity test of the nanoparticles and cisplatin. Relative cell viability of (a) 4T1, (b) SKOV3, (c) A2780 and (d) A2780DDP cells upon treatment with NP1, NP2, Comp-NPs, and cisplatin (CisPt) in the dark or upon irradiation (L, 650 nm, 0.1 W cm^{-2}, 60 J cm^{-2}, 10 min). (n = 3 biologically independent samples)

Supplementary Table 2. IC_{50} values of BODIPY of NP1, NP2, Comp-NPs in μg/mL, and cisplatin (CisPt) μM in the dark or upon irradiation (650 nm, 0.1 W cm^{-2}, 60 J cm^{-2}, 10 min) in 4T1, SKOV3, A2780 and A2780DDP cells.

Cell line	4T1		SKOV3		A2780		A2780DDP	
Sample	Dark	Light	Dark	Light	Dark	Light	Dark	Light
NP1	> 6	> 6	> 6	> 6	> 6	> 6	> 6	> 6
NP2	> 6	0.46 ± 0.13	> 6	0.30 ± 0.01	> 6	0.49 ± 0.04	> 6	0.47 ± 0.01
Comp-NPs	> 6	0.47 ± 0.03	> 6	0.32 ± 0.12	> 6	0.47 ± 0.04	> 6	0.45 ± 0.12
CisPt	4.01 ± 0.01	4.91 ± 0.02	4.13 ± 0.14	4.01 ± 0.02	6.34 ± 0.13	6.68 ± 0.03	13.56 ± 0.10	13.8 ± 0.01

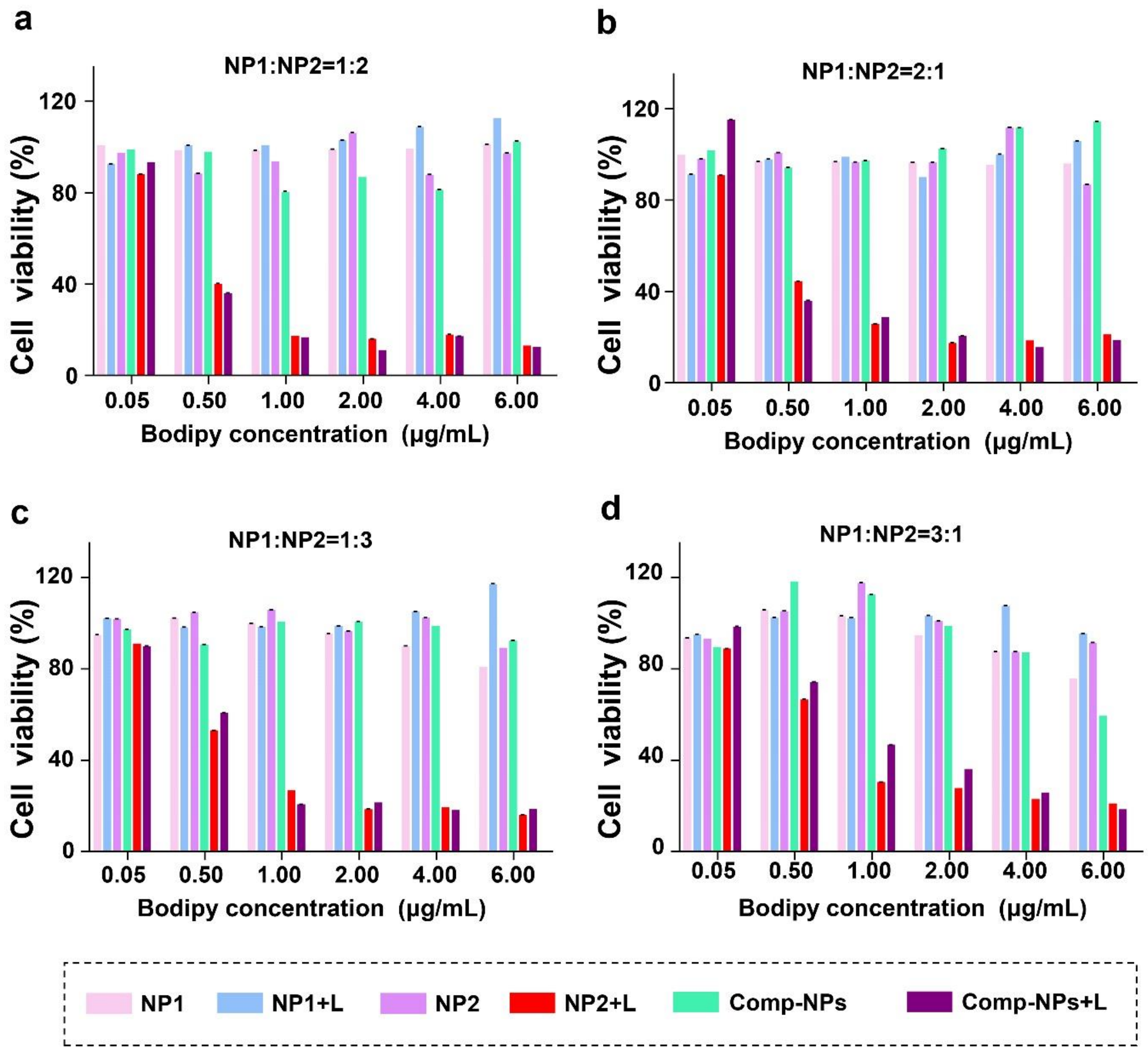

Supplementary Figure 25. In vitro anticancer activity test of Comp-NPs that have been produced with various ratios of NP1 to NP2 (a) 1:2, (b) 2:1, (c) 1:3, (d) 3:1 against 4T1 cells in the dark or upon irradiation (L, 650 nm, 0.1 W cm^{-2}, 60 J cm-^{2}, 10 min). (n = 3 biologically independent samples)

Supplementary Table 3. IC_{50} values of Comp-NPs that have been produced with various ratios of NP1 to NP2 (a) 1:2, (b) 2:1, (c) 1:3, (d) 3:1 against 4T1 cells in the dark or upon irradiation (L, 650 nm, 0.1 W cm^{-2}, 60 J cm-^{2}, 10 min). (*n* = 3)

Ratio of NP1:NP2	Dark	Light
1:2	>6	0.38 ± 0.05
2:1	>6	0.40 ± 0.02
1:3	>6	0.53 ± 0.02
3:1	>6	0.76 ± 0.10

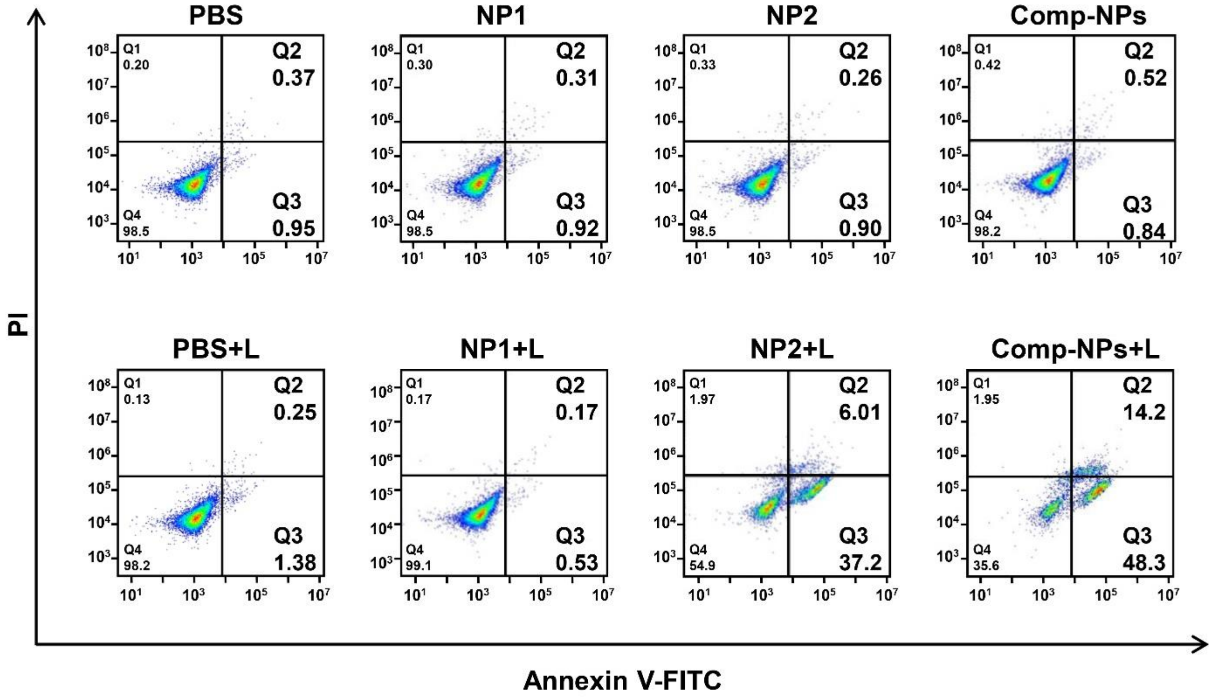

Supplementary Figure 26. Analysis of the cell death of 4T1 cells upon treatment with NP1, NP2, and Comp-NPs, in the dark or upon irradiation (L, 650 nm, 0.1 W cm^{-2}, 30 J cm^{-2}, 5 min). (*n* = 3 biologically independent samples)

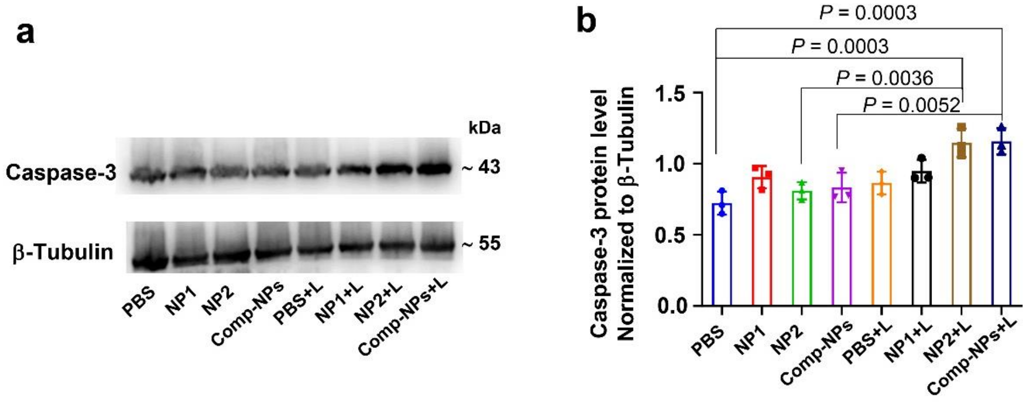

Supplementary Figure 27. (a) Western Blot of the caspase-3 protein expression levels in 4T1 cells after treatment with NP1, NP2, and Comp-NPs, in the dark or upon irradiation (L, 650 nm, 0.1 W cm^{-2}, 30 J cm^{-2}, 5 min). (b) Caspase protein expression levels through semiquantitative analysis from the Western Blot in (a) (n = 3 biologically independent cells). β-Tubulin was used as a reference. Error bars represent mean ± SD. Statistical analysis was performed by one-way ANOVA with a Tukey's multiple comparisons test.

Full-scale pictures:

Caspase-3 (left, anti-caspase-3 antibody, ab184787, 1:1000; Peroxidase-Conjugated Goat Anti-Rabbit IgG (H + L), CAT:33101ES60, 1:5000). β-Tubulin (right, anti-β-tubulin antibody, ab78078, 1:1000; Peroxidase-Conjugated Goat Anti-Mouse IgG (H + L), CAT:33201ES60, 1:5000)

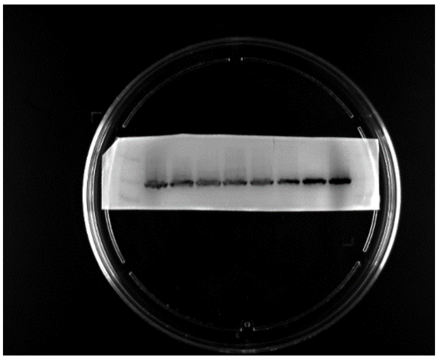

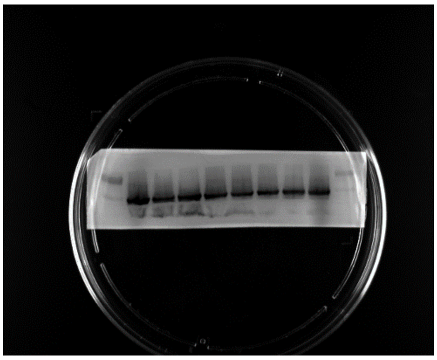

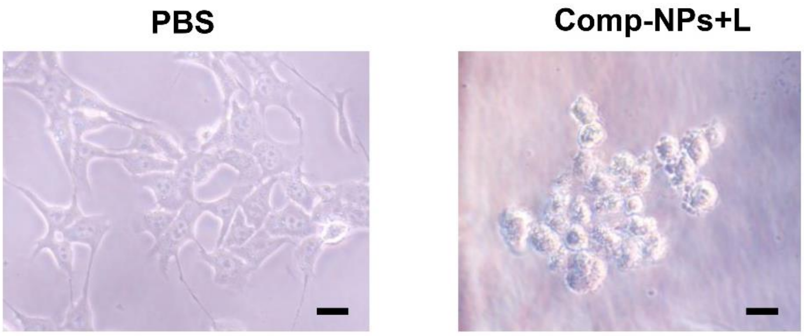

Supplementary Figure 28. Light microscopy images of 4T1 cancer cells upon incubation with phosphate-buffered saline (left) or Comp-NPs upon irradiation (L, 650 nm, 0.1 W cm^{-2}, 30 J cm^{-2}, 5 min). The experiment was repeated independently 3 times with similar results. scale bar = 20 μm.

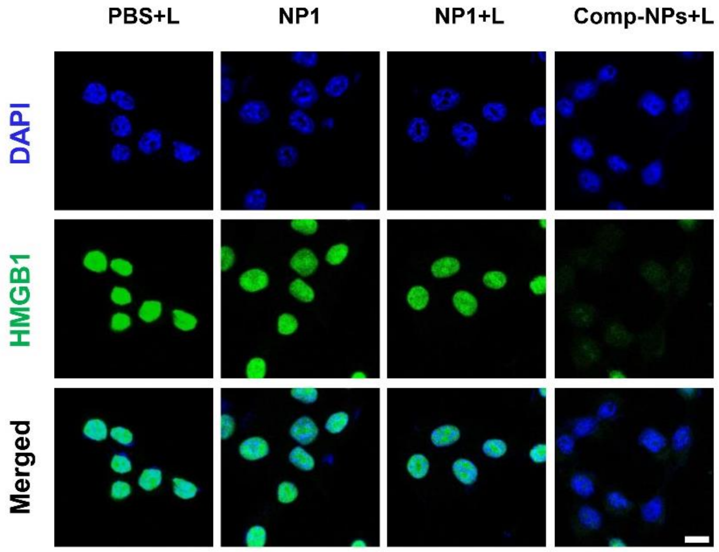

Supplementary Figure 29. CLSM images of 4T1 incubated with HMGB1 protein fluorescent probe (green) and DAPI (blue) upon various treatments. The experiment was repeated independently 3 times with similar results. scale bar = 10 μm.

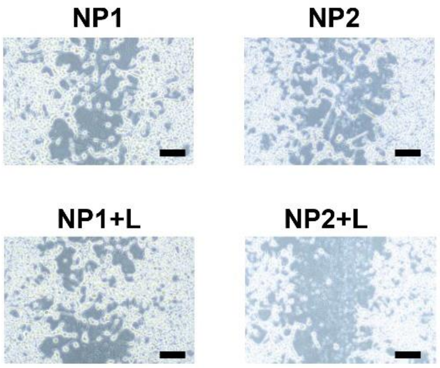

Supplementary Figure 30. Cell migration wound healing assay of 4T1 cells upon various treatments in the light (650 nm, 0.1 W cm^{-2}, 30 J cm^{-2}, 5 min). The experiment was repeated independently 3 times with similar results. scale bar = 50 μm.

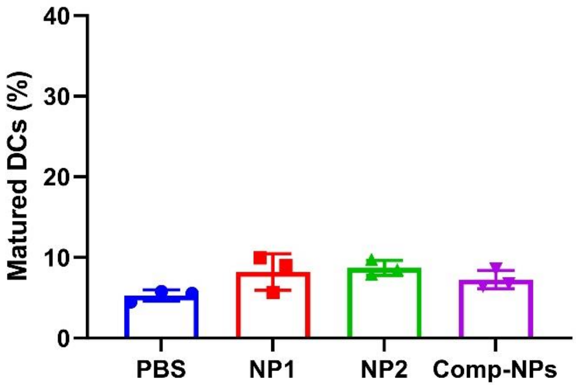

Supplementary Figure 31. Flow cytometry examination of DCs maturation upon various treatments in the dark (*n* = 3 biologically independent samples). Error bars represent mean ± SD.

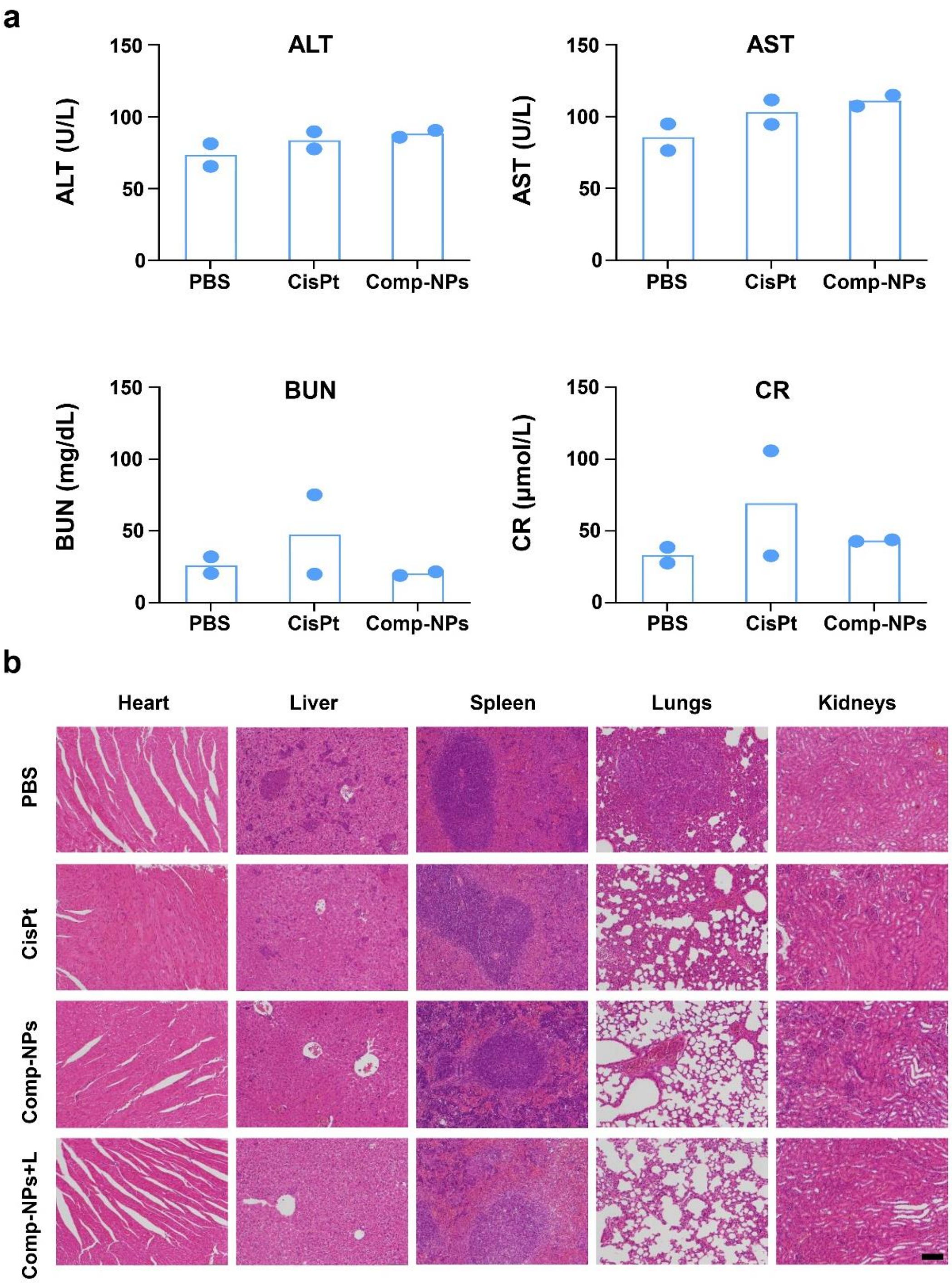

Supplementary Figure 32. *In vivo* systemic toxicities study. (a) Quantification of the levels of various serum indicators upon various treatments of 4T1 tumor bearing mice (n = 2 mice). (b) H&E stain of organs upon various treatments of 4T1 tumor bearing mice. The experiment was repeated independently 3 times with similar results. scale bar = 100 µm. (The images are representative of 5 mice per group).

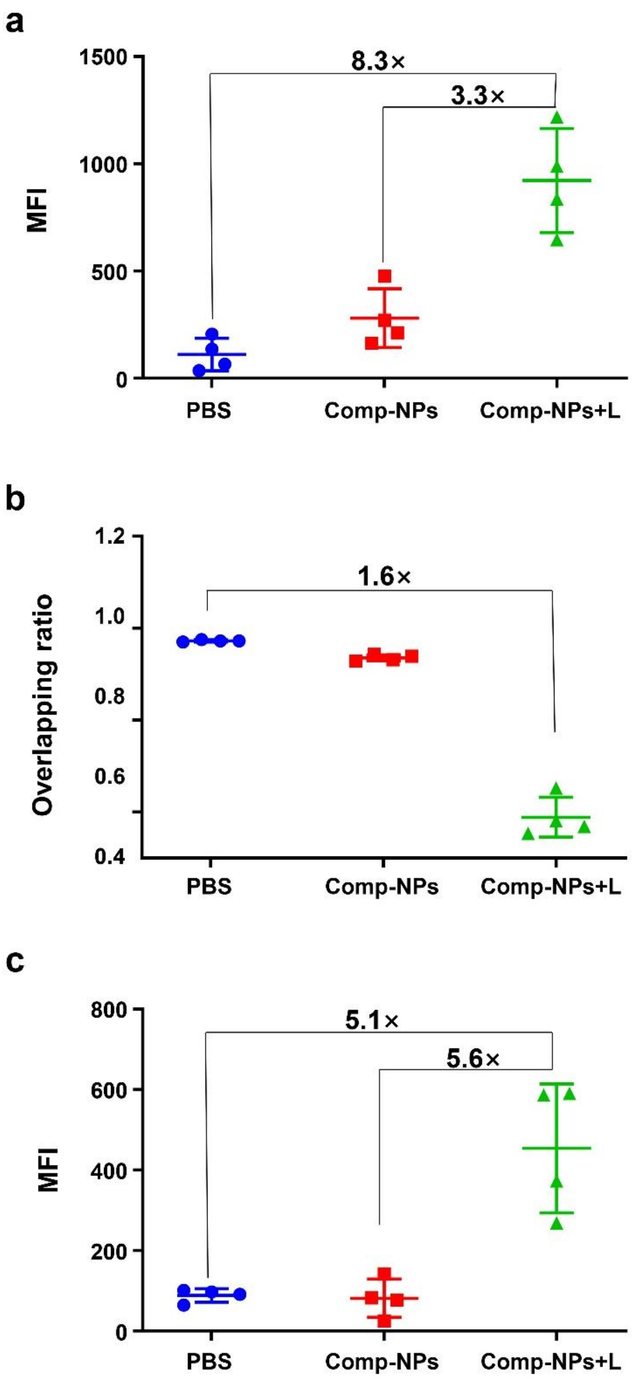

Supplementary Figure 33. Semi-quantifications of mean fluorescence intensity of Figure 4B. (a) Quantifications of mean fluorescence intensity of the CRT specific probe from Figure 4B top panel (n = 4 biologically independent samples). Error bars represent mean ± SD. (b) Quantifications of the ratio of the HMGB1 protein specific probe and the nucleus stain DAPI from Figure 4B middle panel (n = 4 biologically independent samples). Error bars represent mean ± SD. (c) Quantifications of mean fluorescence intensity of the $CD8^+$ T cells specific probe from Figure 4B bottom panel (n = 4 biologically independent samples). Error bars represent mean ± SD.

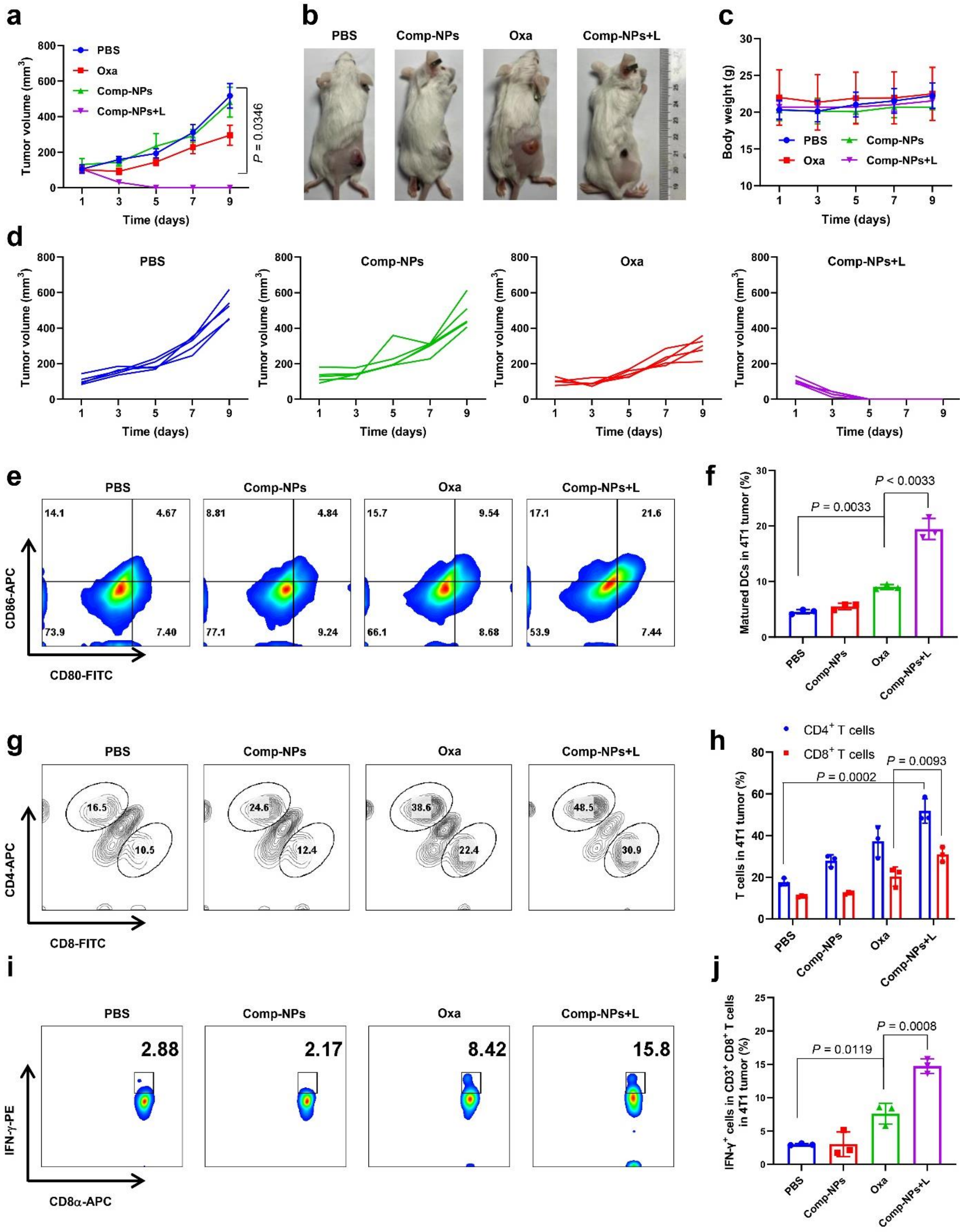

Supplementary Figure 34. In vivo anticancer efficacy on 4T1 tumor-bearing mice. (a) Tumor growth curves of 4T1 tumor-bearing mice upon different treatments with Comp-NPs (6 mg BODIPY kg^{-1}) or Oxa (0.5 mg Pt kg^{-1}) in the dark or upon irradiation (650 nm, 0.1 W cm^{-2}, 60 J cm^{-2}, 10 min) (n = 5 mice). Error bars represent mean ± SD. Statistical analysis was performed by one-way ANOVA with a Tukey's multiple comparisons test. (b) Photographs of the tumor

after the treatment. (c) Monitoring of the weight of the animal model. (d) Individual tumor growth inhibition curves. (n = 5 mice) Error bars represent mean ± SD. (e, f) Flow cytometry plot and quantification of $CD80^{+}CD86^{+}$ dendritic cells gated on $CD11c^{+}$ cells in the tumorous tissue. (n = 3 mice). Error bars represent mean ± SD. Statistical analysis was performed by one-way ANOVA with a Tukey's multiple comparisons test. (g, h) Flow cytometry plot and quantification of $CD8^{+}$ T cells and $CD4^{+}$ T cells gated on $CD3^{+}$ cells in the tumorous tissue. (n = 3 mice) Error bars represent mean ± SD. Statistical analysis was performed by one-way ANOVA with a Tukey's multiple comparisons test. (i, j) Flow cytometry plot and quantification of IFN-$\gamma^{+}CD8^{+}$ T cells gated on $CD3^{+}$ $CD8^{+}$ T cells in the tumorous tissue. (n = 3 mice). Error bars represent mean ± SD. Statistical analysis was performed by one-way ANOVA with a Tukey's multiple comparisons test.

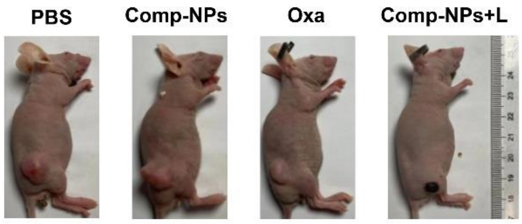

Supplementary Figure 35. Photographs of the animals after various treatments.

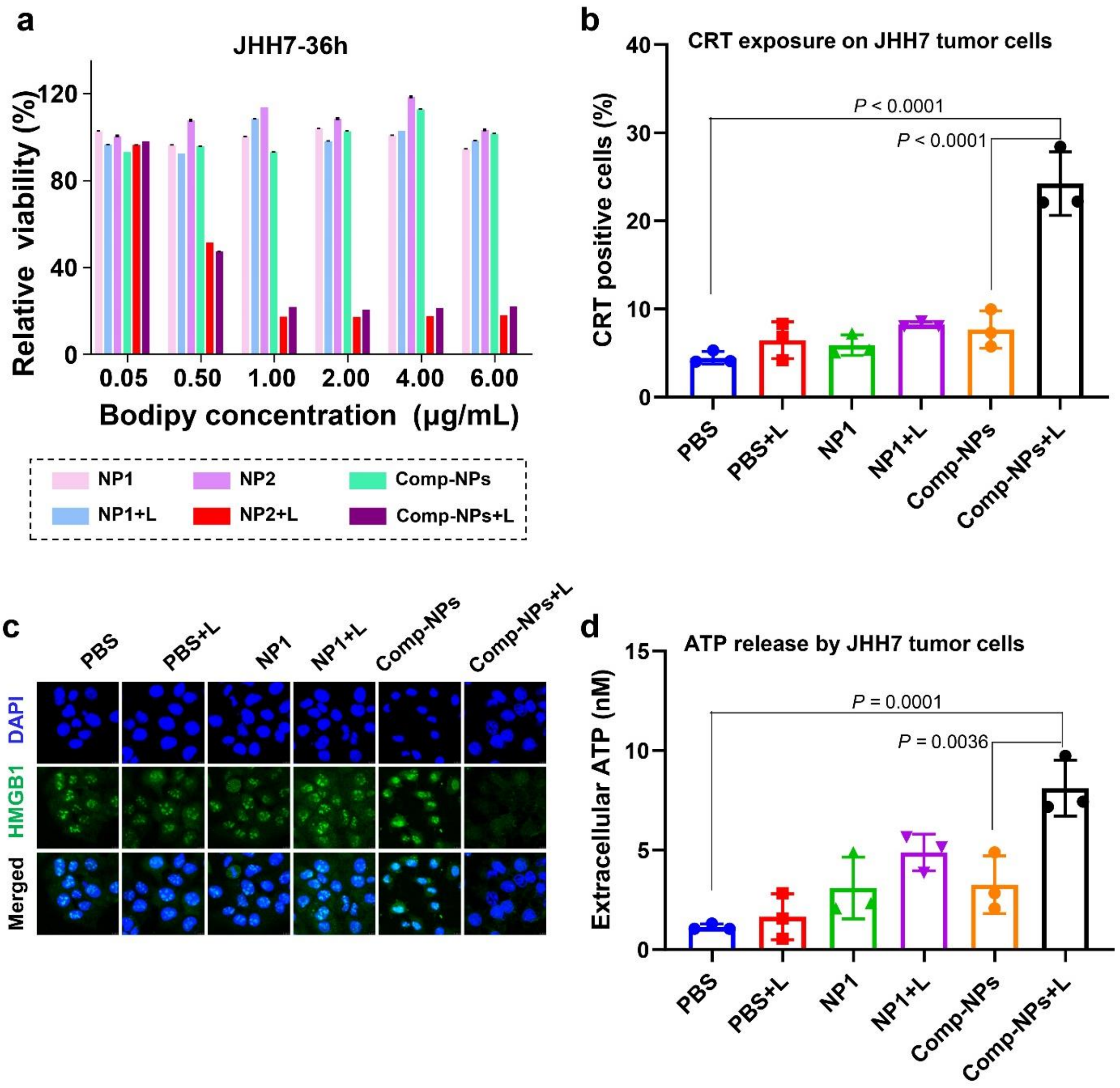

Supplementary Figure 36. *In vitro* anticancer activity test and ICD effect of Comp-NPs on JHH7 cells. (a) Relative cell viability of JHH7 cells. (b) Quantification of the translocation of CRT to the cell surface of JHH7 cells upon various treatments in the light (650 nm, 0.1 W cm^{-2}, 2 min) by flow cytometry (n = 3 biologically independent samples). Error bars represent mean ± SD. Statistical analysis was performed by one-way ANOVA with a Tukey's multiple comparisons test. (c) CLSM images of JHH7 cells incubated with human specific HMGB1 protein fluorescent probe (green) and DAPI (blue) upon various treatments. (n = 3 biologically independent samples) (d) Quantification of extracellular adenosine triphosphate (ATP) in JHH7 cells upon treatment with PBS, NP1, Comp-NPs in the dark or upon irradiation (n = 3 biologically independent samples). Error bars represent mean ± SD. Statistical analysis was performed by one-way ANOVA with a Tukey's multiple comparisons test.

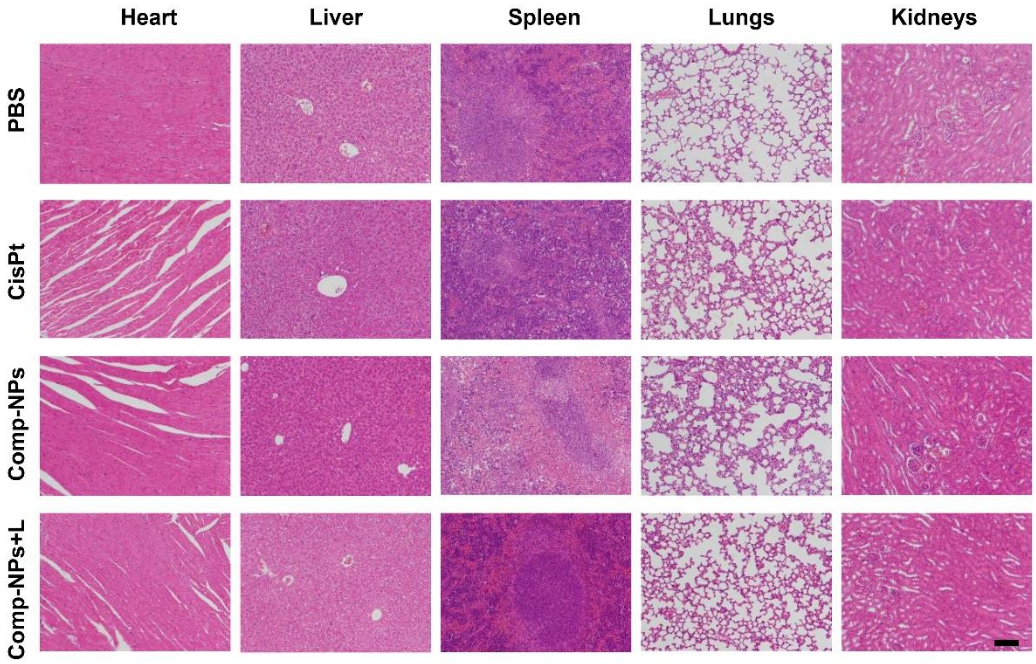

Supplementary Figure 37. H&E staining of organs upon various treatments of HCC tumor bearing mice. The experiment was repeated independently 3 times with similar results. scale bar = 100 μm.

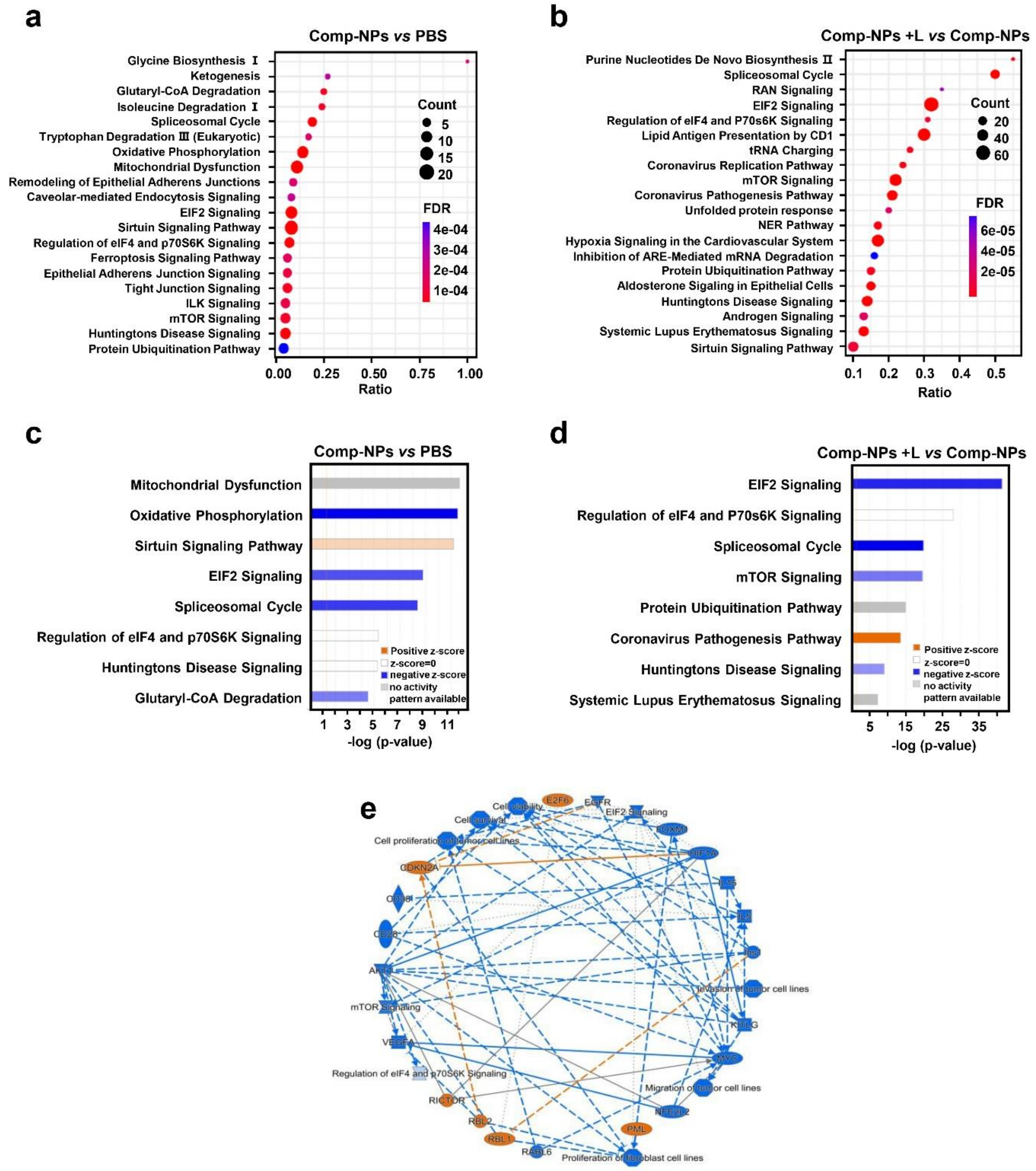

Supplementary Figure 38. Proteomic KEGG pathway enrichment analysis upon different treatments. KEGG pathway enrichment analysis upon treatment with (a) PBS and Comp-NPs; (b) Comp-NPs and Comp-NPs exposure to irradiation. Positive and negative correlation of differential pathway analysis upon treatment with (c) PBS and Comp-NPs; (d) Comp-NPs and Comp-NPs exposure to irradiation. (e) Expression of mTOR pathway-related pathways. (n = 3 biologically independent samples)

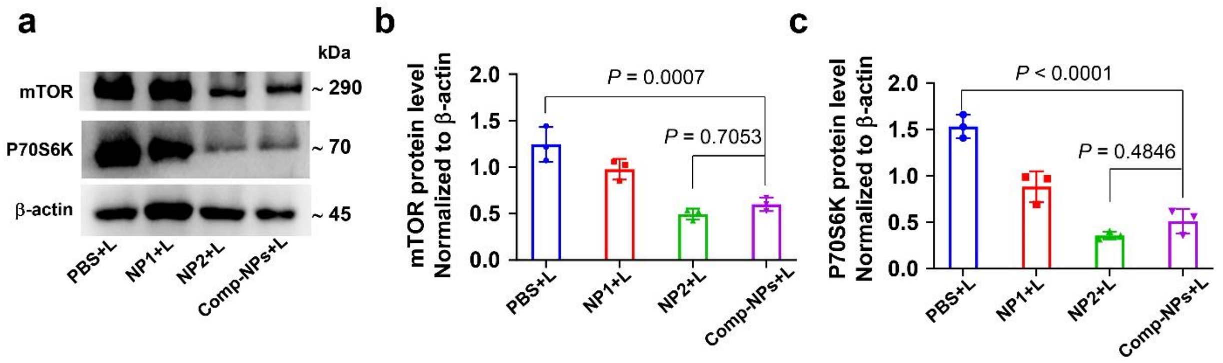

Supplementary Figure 39. (a) Western Blot of the mTOR and P70S6K protein expression levels in 4T1 cells after treatment with NP1, NP2, and Comp-NPs upon irradiation (L, 650 nm, 0.1 W cm^{-2}, 30 J cm^{-2}, 5 min). (b) mTOR protein expression levels through semiquantitative analysis from the Western Blot in (a) (n = 3 biologically independent cells). Error bars represent mean ± SD. Statistical analysis was performed by one-way ANOVA with a Tukey's multiple comparisons test. (c) P70S6K protein expression levels through semiquantitative analysis from the Western Blot in (a) (n = 3 biologically independent cells). Error bars represent mean ± SD. Statistical analysis was performed by one-way ANOVA with a Tukey's multiple comparisons test. β-actin was used as a reference.

Full-scale pictures:

β-actin (left, anti-β-actin antibody, ab8226, 1:1000, Peroxidase-Conjugated Goat Anti-Mouse IgG (H + L), CAT:33201ES60, 1:5000;) P70S6K (middle, anti-P70 S6 Kinase (phosphor T389) antibody, ab2571, 1:1000; Peroxidase-Conjugated Goat Anti-Rabbit IgG (H + L), CAT:33101ES60, 1:5000), mTOR (right, anti-mTOR antibody, ab32028, 1:1000; Peroxidase-Conjugated Goat Anti-Rabbit IgG (H + L), CAT:33101ES60, 1:5000).

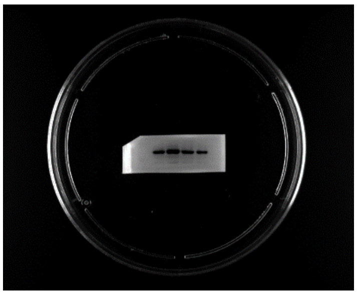
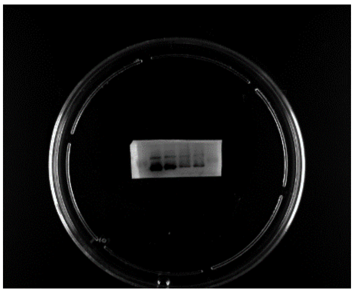
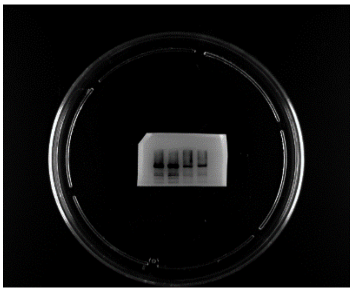

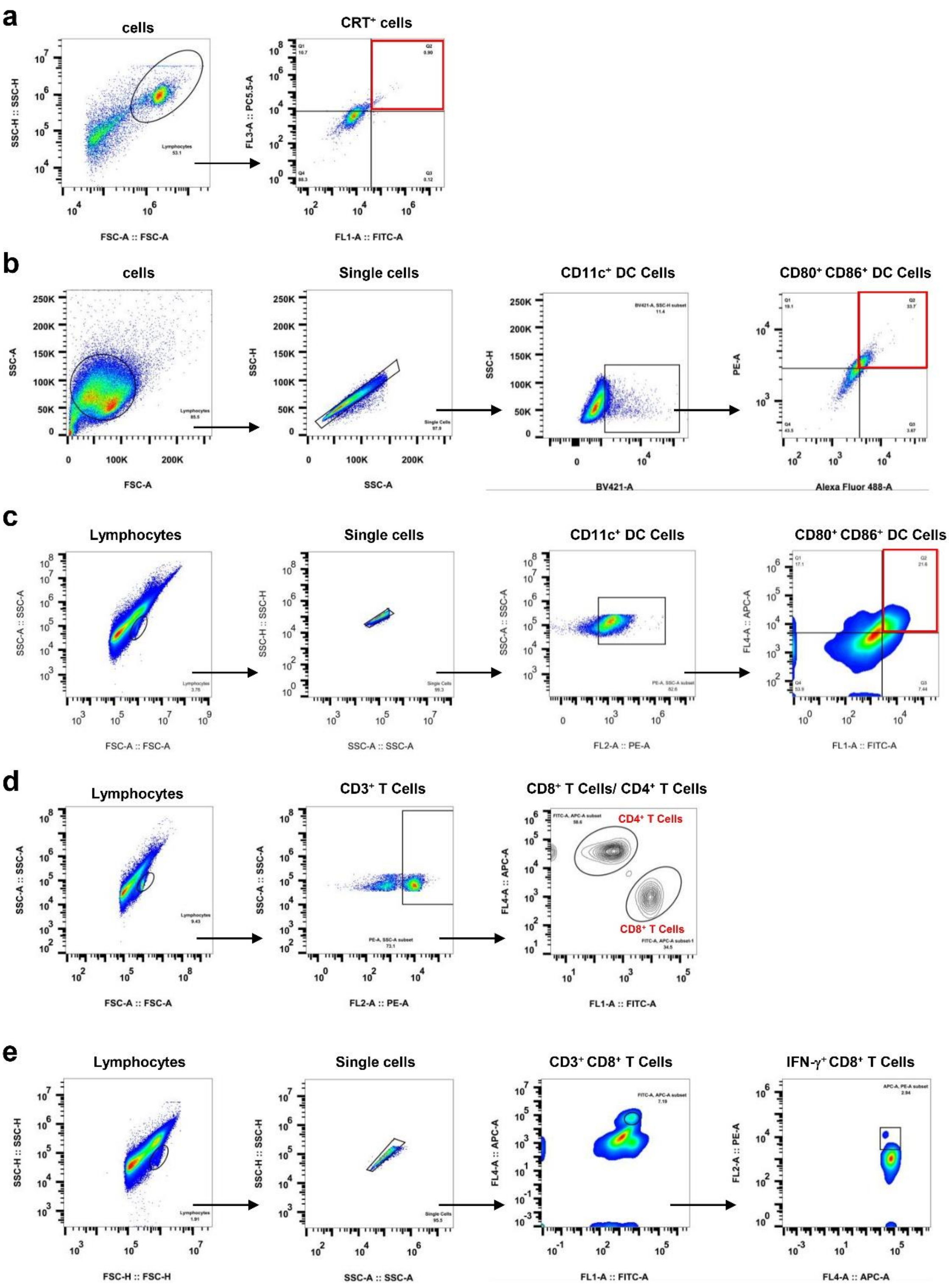

Supplementary Figure 40. The gating strategies of flow cytometry analyses for (a) CRT positive cells (related to Figure 3d, Supplementary Figure 36b). (b) Matured DCs in vitro (related to Figure 3i, j, Supplementary Figure 31). (c) Matured DCs in vivo (related to Figure 5c-f, Supplementary Figure 34e, f). (d) $CD3^+CD4^+CD8^+$ T cells (related to Figure 5g, h, Supplementary Figure 34g, h). (e) IFN-γ^+ $CD8^+$ T cells (related to Supplementary Figure 34i, j).

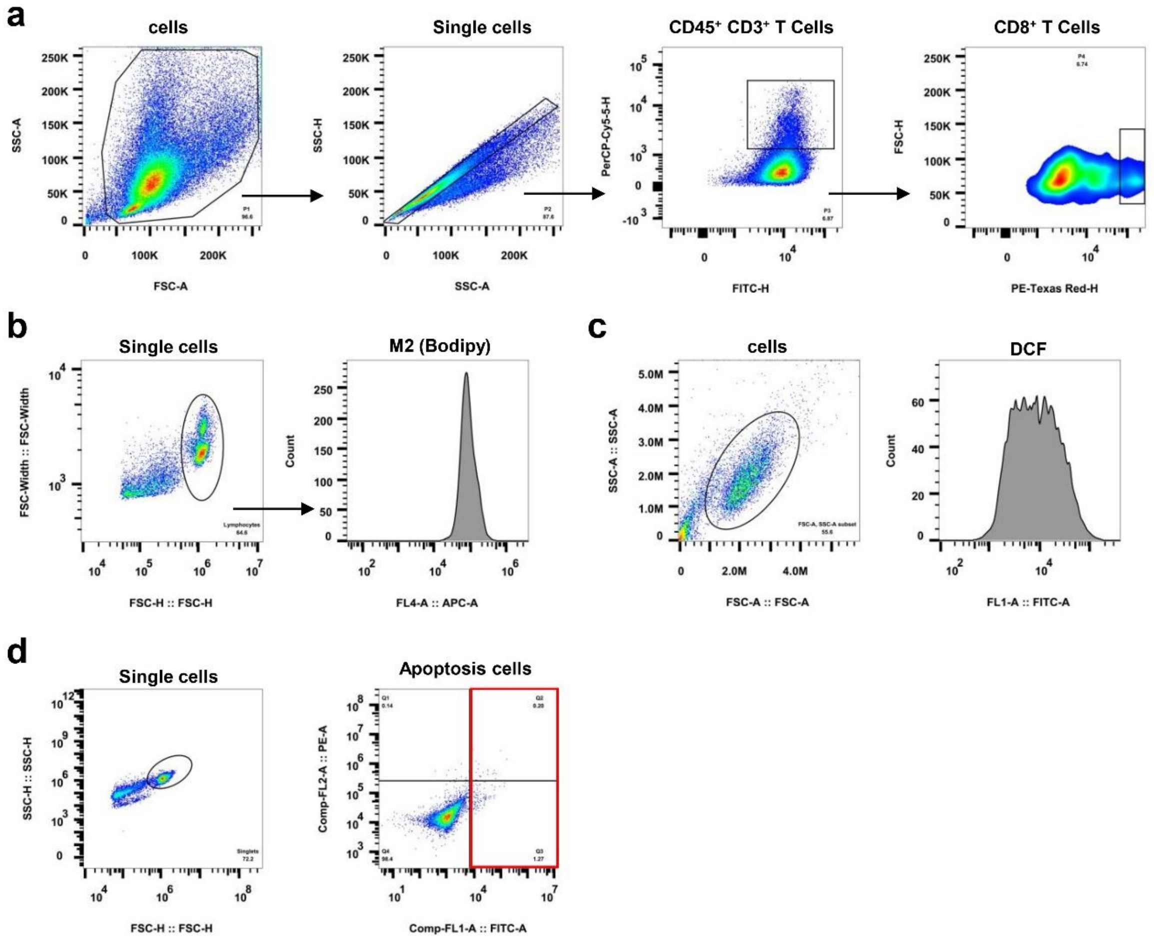

Supplementary Figure 41. The gating strategies of flow cytometry analyses for (a) $CD3^{+}CD8^{+}$ T cells (related to Figure 10d). (b) cellular uptake (related to Supplementary Figure 22a, b). (c) ROS generation (related to Supplementary Figure 23a, b). (d) Apoptosis cells (Supplementary Figure 26).
