# Supplementary material for: Theranostic imaging and multimodal photodynamic therapy and immunotherapy using the mTOR signaling pathway
Source: Nat Commun. 2023 Sep 2;14:5350. doi: 10.1038/s41467-023-40826-5 (PMC10475087; doi:10.1038/s41467-023-40826-5)
Supplement: Supplementary file 2 — Reporting Summary [file 41467_2023_40826_MOESM2_ESM.pdf]

nature portfolio

Corresponding author(s): Karges & Xiao

Last updated by author(s): Mar 19, 2023

Reporting Summary

Nature Portfolio wishes to improve the reproducibility of the work that we publish. This form provides structure for consistency and transparency in reporting. For further information on Nature Portfolio policies, see our Editorial Policies and the Editorial Policy Checklist.

Statistics

For all statistical analyses, confirm that the following items are present in the figure legend, table legend, main text, or Methods section.

n/a	Confirmed	
☐	☒	The exact sample size (*n*) for each experimental group/condition, given as a discrete number and unit of measurement
☐	☒	A statement on whether measurements were taken from distinct samples or whether the same sample was measured repeatedly
☐	☒	The statistical test(s) used AND whether they are one- or two-sided<br>*Only common tests should be described solely by name; describe more complex techniques in the Methods section.*
☒	☐	A description of all covariates tested
☒	☐	A description of any assumptions or corrections, such as tests of normality and adjustment for multiple comparisons
☐	☒	A full description of the statistical parameters including central tendency (e.g. means) or other basic estimates (e.g. regression coefficient) AND variation (e.g. standard deviation) or associated estimates of uncertainty (e.g. confidence intervals)
☐	☒	For null hypothesis testing, the test statistic (e.g. *F*, *t*, *r*) with confidence intervals, effect sizes, degrees of freedom and *P* value noted<br>*Give P values as exact values whenever suitable.*
☒	☐	For Bayesian analysis, information on the choice of priors and Markov chain Monte Carlo settings
☒	☐	For hierarchical and complex designs, identification of the appropriate level for tests and full reporting of outcomes
☒	☐	Estimates of effect sizes (e.g. Cohen's *d*, Pearson's *r*), indicating how they were calculated

Our web collection on statistics for biologists contains articles on many of the points above.

Software and code

Policy information about availability of computer code

Data collection	NMR data were collected using an AVANCE III HD (Bruker);<br>MALDI-TOF-MS data were collected using an Autoflex III (Bruker);<br>Photofluorescence (PL) spectra of M1, NP1 and NP3were recorded on a fluorescence spectrometer (FLS980);<br>Cell uptake, ROS generation, CD8+Tcells, CRT, HMGB1 protein inside the cells was assessed using CLSM (LSM-800, ZEISS, Germany);<br>Cell uptake, ROS production, CRT positive cells, CD80+CD86+ cells, CD3+CD4+CD8+ T cells and IFN+ CD8+ T cells were measured with a flow cytometry (FACSAria II ,BD &Cytomics FC500, Beckman);<br>The phosphorescence was recorded on an In Vivo Imaging System (IVIS, Perkin Elmer);
Data analysis	1H-NMR and 13C-NMR data was analyzed by MestReNova;<br>Statistical analyses were performed on Graphpad prism 9.0;<br>Flow cytometry data were analyzed on FlowJo software package (Flowjo V10);<br>Images were processed with Image-J;

For manuscripts utilizing custom algorithms or software that are central to the research but not yet described in published literature, software must be made available to editors and reviewers. We strongly encourage code deposition in a community repository (e.g. GitHub). See the Nature Portfolio guidelines for submitting code & software for further information.

Data

Policy information about availability of data

All manuscripts must include a data availability statement. This statement should provide the following information, where applicable:

- Accession codes, unique identifiers, or web links for publicly available datasets
- A description of any restrictions on data availability
- For clinical datasets or third party data, please ensure that the statement adheres to our policy

The authors declare that all data supporting the findings of this study are available within the paper and its supplementary information files. Proteomics data that support the findings of this study have been deposited in the Proteomics Identifications Database (PRIDE).

Research involving human participants, their data, or biological material

Policy information about studies with human participants or human data. See also policy information about sex, gender (identity/presentation), and sexual orientation and race, ethnicity and racism.

Reporting on sex and gender	A human hepatocellular carcinoma (grade Ⅲ, 47-vear-old man) was used for PDX model construction. A human breast carcinoma (ER-PR-HER2-,grade ⅢC) was used for PDX model construction.
Reporting on race, ethnicity, or other socially relevant groupings	-
Population characteristics	-
Recruitment	The human hepatocellular carcinoma (grade Ⅲ, 47-vear-old man) from the Fifth Medical Center of Chinese PLA General Hospital. The human breast carcinoma (ER-PR-HER2-,grade ⅢC) from Cancer Hospital Chinese Academy of Medical Sicences.
Ethics oversight	A human hepatocellular carcinoma (grade Ⅲ, 47-vear-old man) was obtained from The Fifth Medical Center of Chinese PLA General Hospital, with the approval of the ethical review board at Chinese PLA General Hospital and informed consent from the patient.<br>A human breast carcinoma (ER-PR-HER2-,grade ⅢC) was obtained from Cancer Hospital Chinese Academy of Medical Sicences, with the approval of the ethical review board at Cancer Hospital Chinese Academy of Medical Sicences and informed consent from the patient.

Note that full information on the approval of the study protocol must also be provided in the manuscript.

Field-specific reporting

Please select the one below that is the best fit for your research. If you are not sure, read the appropriate sections before making your selection.

☒ Life sciences ☐ Behavioural & social sciences ☐ Ecological, evolutionary & environmental sciences

For a reference copy of the document with all sections, see nature.com/documents/nr-reporting-summary-flat.pdf

Life sciences study design

All studies must disclose on these points even when the disclosure is negative.

Sample size	sample size were provided in the figure legends for each experiment.
Data exclusions	no data was excluded
Replication	data was fully replicable
Randomization	All experimental samples or models including in vitro cells and in vivo mice were randomly allocated to each group
Blinding	No blinding was performed in this study. The investigators should keep careful track of protocols because that most of the experiments needed multiple treatments (including formulation, cells or mouse tumor treatment, sample collection, and so on)

Reporting for specific materials, systems and methods

We require information from authors about some types of materials, experimental systems and methods used in many studies. Here, indicate whether each material, system or method listed is relevant to your study. If you are not sure if a list item applies to your research, read the appropriate section before selecting a response.

Materials & experimental systems

n/a	Involved in the study
	[x] Antibodies
	[x] Eukaryotic cell lines
[x]	Palaeontology and archaeology
	[x] Animals and other organisms
[x]	Clinical data
[x]	Dual use research of concern
[x]	Plants

Methods

n/a	Involved in the study
[x]	ChIP-seq
	[x] Flow cytometry
[x]	MRI-based neuroimaging

Antibodies

Antibodies used

anti-CRT antibody (Abcam, ab92516, 1:50)
HMGB1 protein antibody (Abcam, ab18256, 1:50)
Alexa Fluor 594-conjugated secondary antibody (Abcam, ab150080, 1:500)
Alexa Fluor 488-conjugated secondary antibody (Abcam, ab150077, 1:500)
anti-caspase-3 antibody, ab 184787, 1:1000;
anti-β-tubulin antibody, ab78078, 1:1000,
anti-β-actin antibody, ab8226, 1:1000;
anti-P70 S6 Kinase (phosphor T389) antibody, ab2571, 1:1000;
anti-mTOR antibody, ab32028, 1:1000)
Peroxidase-Conjugated Goat Anti-Rabbit IgG (H + L), CAT:33101ES60, 1:5000
Peroxidase-Conjugated Goat Anti-Mouse IgG (H + L), CAT:33201ES60, 1:5000
anti-CD3-PE (elabscience, E-AB-F1013D, 1:100)
anti-CD4-PC5.5 (elabscience, E-AB-F1097J, 1:100)
anti-CD8-FITC (elabscience, E-AB-F1104C, 1:100)
anti-CD11c-PE (elabscience, E-AB-F0991D, 1:100)
anti-CD80-FITC (elabscience, E-AB-F0992C, 1:100)
anti-CD86-APC (elabscience, E-AB-F0994C, 1:100)
anti-mouse IFN-γ(biolegend, 505810,1:00)

Validation

Validation of each antibody was done under standard information offered by the supplier.

Eukaryotic cell lines

Policy information about cell lines and Sex and Gender in Research

Cell line source(s)

4T1 cells, SKOV3 cells, JHH7 cells, A2780, A2780 DDP cells were sourced from American Type Culture Collection (ATCC).

Authentication

Cells were used without modification once received from the supplier and therefore were not authenticated.

Mycoplasma contamination

all cell lines were tested and verified to be free of mycoplasma

Commonly misidentified lines
(See ICLAC register)

no commonly misidentified cell lines were used.

Animals and other research organisms

Policy information about studies involving animals; ARRIVE guidelines recommended for reporting animal research, and Sex and Gender in Research

Laboratory animals

Balb/c mice, Balb/c nude (4 weeks old, female) were purchased from SPF (Beijing) Biotechnology Co., Ltd. (China), and kept under specific pathogen free (SPF) condition for one week before the studies, with free access to standard food and water. All animal experiments were conducted in compliance with the ethical regulations for animal testing and received approval from the Peking University Institutional Animal Care and Use Committee (LA2021316). Animals were housed in groups of 4–5 mice per cage, maintained at a temperature of ~25 °C in a humidity-controlled environment with a 12 h light/dark cycle.

Wild animals

this study did not involve wild animals

Reporting on sex

female animals

Field-collected samples

The study did not involve samples collected from the field.

Ethics oversight

The experiments received approval from the People's Liberation Army (PLA) General Hospital and the Clinical Trial Registry (ChiCTR2100047481). All animal experiments were conducted in compliance with the ethical regulations for animal testing and

received approval from the Peking University Institutional Animal Care and Use Committee (LA2021316).

Note that full information on the approval of the study protocol must also be provided in the manuscript.

Flow Cytometry

Plots

Confirm that:

- [x] The axis labels state the marker and fluorochrome used (e.g. CD4-FITC).
- [x] The axis scales are clearly visible. Include numbers along axes only for bottom left plot of group (a 'group' is an analysis of identical markers).
- [x] All plots are contour plots with outliers or pseudocolor plots.
- [x] A numerical value for number of cells or percentage (with statistics) is provided.

Methodology

Sample preparation	Cells were trypsinized, harvested, and subjected to PBS washing prior to flow cytometric analysis. In some experiments, cells were stained with antibodies or probes following the manufacturer's protocols, and subsequently analyzed using flow cytometry.
Instrument	Automatic double laser high speed cell analysis sorting system（FACSAria II）
Software	FlowJo software package (Flowjo V10)
Cell population abundance	Cell sorting was not performed in the flow cytometry experiments.
Gating strategy	Typically, cells were initially gated based on FSC-A and SSC-A, and single cells were gated using SSC-A and SSC-H. Subsequently, the analysis of probes and biomarkers was performed on the single cell population.

- [x] Tick this box to confirm that a figure exemplifying the gating strategy is provided in the Supplementary Information.
